# Deregulation of the Protocadherin Gene *FAT1* Alters Muscle Shapes: Implications for the Pathogenesis of Facioscapulohumeral Dystrophy

**DOI:** 10.1371/journal.pgen.1003550

**Published:** 2013-06-13

**Authors:** Nathalie Caruso, Balàzs Herberth, Marc Bartoli, Francesca Puppo, Julie Dumonceaux, Angela Zimmermann, Simon Denadai, Marie Lebossé, Stephane Roche, Linda Geng, Frederique Magdinier, Shahram Attarian, Rafaelle Bernard, Flavio Maina, Nicolas Levy, Françoise Helmbacher

**Affiliations:** 1 Aix-Marseille Université, CNRS, IBDML UMR 7288, Parc Scientifique de Luminy, Case 907, Marseille, France; 2 Aix-Marseille Université, Faculté de Médecine de la Timone, INSERM UMR 910, Marseille, France; 3 INSERM U974, UMR 7215 CNRS, Institut de Myologie, UM 76 Université Pierre et Marie Curie, Paris, France; 4 Division of Human Biology, Fred Hutchinson Cancer Research Center, Seattle, Washington, United States of America; 5 AP-HM, Neurologie, maladies neuro-musculaires, Hôpital de la Timone, Marseille, France; 6 AP-HM, Département de Génétique Médicale, Hôpital d'enfants Timone, Marseille, France; The Jackson Laboratory, United States of America

## Abstract

Generation of skeletal muscles with forms adapted to their function is essential for normal movement. Muscle shape is patterned by the coordinated polarity of collectively migrating myoblasts. Constitutive inactivation of the protocadherin gene *Fat1* uncoupled individual myoblast polarity within chains, altering the shape of selective groups of muscles in the shoulder and face. These shape abnormalities were followed by early onset regionalised muscle defects in adult *Fat1*-deficient mice. Tissue-specific ablation of *Fat1* driven by *Pax3-cre* reproduced muscle shape defects in limb but not face muscles, indicating a cell-autonomous contribution of *Fat1* in migrating muscle precursors. Strikingly, the topography of muscle abnormalities caused by *Fat1* loss-of-function resembles that of human patients with facioscapulohumeral dystrophy (FSHD). *FAT1* lies near the critical locus involved in causing FSHD, and *Fat1* mutant mice also show retinal vasculopathy, mimicking another symptom of FSHD, and showed abnormal inner ear patterning, predictive of deafness, reminiscent of another burden of FSHD. Muscle-specific reduction of *FAT1* expression and promoter silencing was observed in foetal FSHD1 cases. CGH array-based studies identified deletion polymorphisms within a putative regulatory enhancer of *FAT1*, predictive of tissue-specific depletion of *FAT1* expression, which preferentially segregate with FSHD. Our study identifies *FAT1* as a critical determinant of muscle form, misregulation of which associates with FSHD.

## Author Summary

Facioscapulohumeral muscular dystrophy (FSHD) is a hereditary human myopathy affecting groups of skeletal muscles in the face and shoulders. Despite recent advances on the molecular cascade initiated by its main genetic cause, with identification of *DUX4* as the main pathogenic agent, how this leads to the specific clinical picture is still poorly understood. Here, we investigated the role of the *FAT1* protocadherin gene, located near the FSHD locus, which was repressed by *DUX4* in human muscle cells. Disruption of the mouse *Fat1* gene causes muscular and non-muscular phenotypes highly reminiscent of FSHD symptoms. We show that *Fat1* is required in migrating muscle precursors, and that the altered muscle shapes caused by *Fat1* mutations are predictive of early onset defects in muscle integrity in adult mutants, with a topography matching the map of muscles affected in FSHD. In humans, we observed *FAT1* lowering in muscle but not brain of foetal cases with canonical FSHD1, and identified deletions of conserved elements in the *FAT1* locus predictive of changes in *FAT1* expression, that were enriched among FSHD patients. Thus, deregulating *Fat1* in FSHD-related tissues provides a unique means to mimic FSHD symptoms in mice and learn about pathogenesis of this complex disease.

## Introduction

Developmental genetics has provided considerable insight into the regulatory networks controlling overall skeletal muscle development. Perturbation of these common mechanisms is associated with congenital abnormalities of the muscle lineage as well as with later-onset muscle pathologies [1]. In contrast, less is known about the mechanisms of functional diversification within the muscle lineage. Such diversification may be either metabolic - fast versus slow fibres, for example - or morphological, such as the position and shape of individual muscles. Genes controlling diversification too are likely to be of clinical significance [2]–[4], since several human muscular dystrophies do not affect all muscles evenly, but specifically target regionalized groups [5]. This is true for limb girdle muscular dystrophy (LGMD), oculopharyngeal muscular dystrophy (OPMD), myotonic dystrophies with oculomotor involvement, distal myopathies, scapuloperoneal dystrophy, and facioscapulohumeral dystrophy (FSHD) [5]–[6]. In no case, however, is the rationale for this geographic specificity currently understood.

One characteristic example of focal myopathies is FSHD, which affects subsets of muscles in the facial and shoulder areas [6]. The main form of FSHD - FSHD1 - is an autosomal dominant disorder associated with the contraction of an array of 3.3 Kb macrosatellite repeats (*D4Z4*), located at the subtelomeric 4q35 locus [6]. The mechanism by which the *D4Z4* contraction triggers the disease represents one of the most enigmatic conundrums for human geneticists and remains incompletely understood. The *D4Z4* array has been suggested to act as an insulator between telomeres and subtelomeric genes [7]–[8], such that its contraction might result in regulatory changes in neighbouring genes that could in turn alter muscle physiology [6], [9]–[11]. Despite intense focus on deregulated 4q35 genes, including one of the close neighbours, *FRG1* [12], and despite numerous large-scale investigations aimed at uncovering additional relevant candidates, none of the genes reported accounts for all aspects of FSHD, and additional players are still actively sought [6], [9], [13]. An emerging model is that the pathogenic effect of *D4Z4* contraction in FSHD1 is mediated in part by *DUX4*, a retrogene present within D4Z4 repeats themselves encoding a homeobox containing transcription factor that is normally silent in muscle [14]–[15]. In FSHD1 patients, the contraction of the D4Z4 repeat array leads to a change in chromatin structure that facilitates DUX4 expression [16]. Furthermore, the pathogenicity of the D4Z4 contraction requires polymorphisms distal to the last *D4Z4* repeat, that create a polyadenylation signal and thereby stabilize *DUX4* mRNA [17]. This stabilized RNA thus leads to increased expression levels in FSHD muscles of a pathogenic isoform of DUX4, which activity is thought to be toxic for muscles through transcriptional activation of various target genes including *Pitx1* and *p53* [18]–[21]. Another less frequent form of FSHD, clinically identical to FSHD1, is observed in absence of D4Z4 contraction. These cases, referred to as contraction-independent FSHD, include cases called FSHD2, that were shown to exhibit hypomethylated *D4Z4* repeats, recently shown to be caused by mutations in the *SMCHD1* gene [22]. FSHD2 is caused by the combination of such *SMCHD1* mutations with a DUX4 permissive (polyA) context, and also leads to DUX4 overexpression [22]. While FSHD2 cases represent so far the majority of contraction-independent cases, rare cases of contraction-independent FSHD with typical symptoms may also occur without hypomethylation, and be caused by yet unidentified pathogenic contexts. Neither the specificity of *SMCHD1* or of *DUX4* expression nor of its target genes identified so far [18]–[21], [23]–[24], provide sufficient account for the specificity of the muscle map and the non-muscular symptoms that characterize FSHD.

The regional specificity in the map of muscles affected in FSHD suggests that the causal abnormality interferes with a muscle subtype-specific developmental process. A gene involved in functional diversification during muscle development would thus provide a logical candidate to fill this gap. We focused on the cell adhesion molecule FAT1 because Fat-like protocadherins are known modulators of the planar cell polarity (PCP) pathway [25]–[27], a genetic cascade involved in coordinating tissue polarity, morphogenetic movements, and polarized cell flow [28]–[30]. *Fat1* has been reported to be expressed in developing muscles and tendons [31] and to be regulated by muscle developmental genes such as *Pax3*, *Lbx1*, or *Met* [32]–[34]. Thus, FAT1 may control muscle shape through PCP-like mechanisms analogous to those involved in polarized migration of vascular endothelial smooth muscle cells [35].

Here, we report the unexpected finding that *Fat1*-deficient mice reproduce the highly selective muscular and non-muscular aspects of the clinical picture of FSHD. We show that *Fat1* is required during development to shape specific groups of shoulder and facial muscles by modulating the polarity of myoblast migration. While constitutive inactivation of *Fat1* leads to neonatal lethality due to defects in kidney development [36], *Fat1* hypomorphic mice exhibit defects of muscle integrity with a topography prefiguring the map of muscles affected in FSHD. Furthermore, conditional mutagenesis suggests that a cell-autonomous function of *Fat1* in migrating muscle cells may account for a significant part of its muscle shaping function. The human *FAT1* gene is located only 3.6 Mb from the critical FSHD genomic region at 4q35, and emerges as a potential transcriptional target of DUX4 or p53 [18], [37]–[38]. We present evidence of altered *FAT1* levels in some foetal FSHD1 cases, in muscle, but not brain, accompanied with epigenetic modifications characteristic of silenced chromatin. Finally, we identified genetic variants deleting variable lengths of a putative cis-regulatory enhancer in the *FAT1* locus, which segregate with FSHD. Thus, either in presence or absence of D4Z4 contractions, mechanisms leading to tissue-specific deregulation of *FAT1* expression are associated with FSHD and may contribute to causing regional-specific muscle shape abnormalities that prefigure muscle degeneration in the adult.

## Results

### *Fat1* regulates myoblast polarity during planar migration

**Figure 1:**
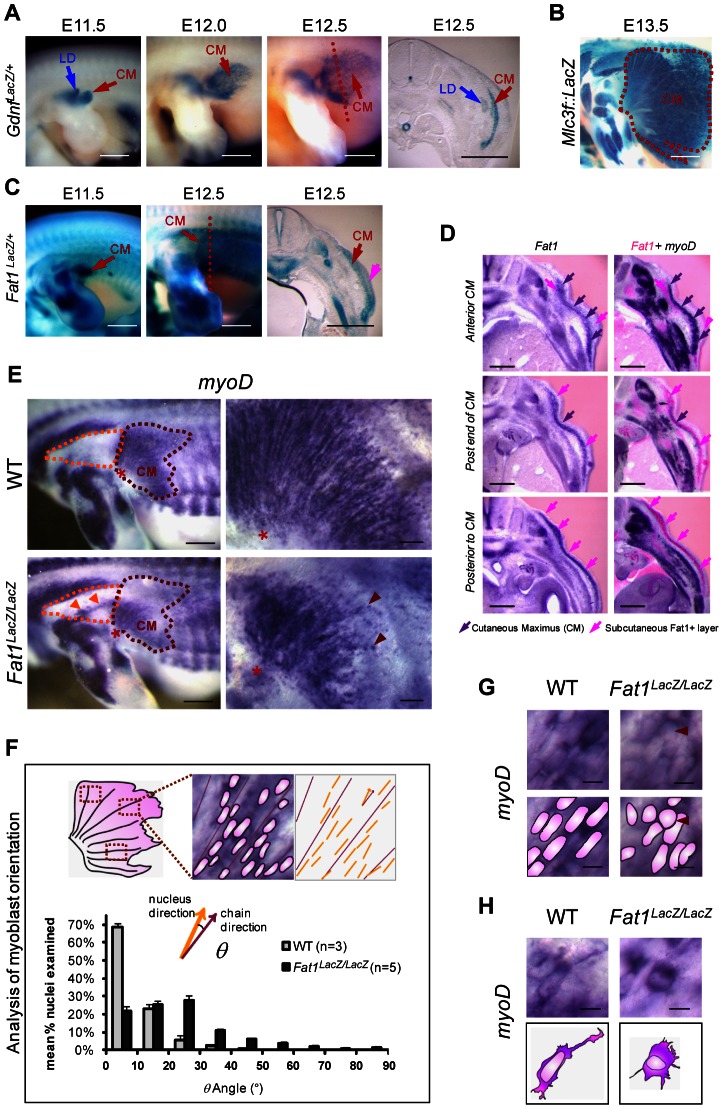
*Fat1* controls the shape of subsets of scapular muscle by modulating myoblast polarity during planar migration. (**A–C**) Reporter gene expression in the forelimb and flank of mouse embryos between E11.5 and E13.5. (**A**) *Gdnf-lacZ* staining labels myoblasts of the *latissimus dorsee* (LD) and *cutaneous maximus* (CM). CM myoblasts migrate away from the brachial plexus to form a subcutaneous muscle sheath, composed of radially-oriented chains of myoblasts. (**B**) At E13.5, *MLC3f-lacZ* staining reveals the characteristic fan-shaped form of the CM (dotted white purple line) as compared to other limb muscles. (**C**) *Fat1* expression detected using the *lacZ* gene trap allele KST249 (*Fat1^LacZ^*) is selectively localized within the CM and in surrounding tissue (pink arrow). (**D**) CM myoblasts express *Fat1* and migrate towards an increasing gradient of *Fat1* expression. Alternate vibratome cross-sections of a wild type E12.5 embryo were hybridized with *Fat1* (left column) and *MyoD* (purple, right column) RNA probes. Photographs of adjacent sections were superimposed (photoshop) after conversion of *Fat1* staining color in pink (right column; *Fat1* in pink, *MyoD* in purple). *MyoD* expression is used as a marker of the muscle lineage. Superimposition was meant to compare the relative levels of *Fat1* expression within and around the *cutaneous maximus* (CM) muscle (indicated with purple arrows), at three consecutive antero-posterior positions, respectively within the CM (top row), at the posterior end (middle row), and posterior to the caudal extremity of the CM at that stage. CM myoblasts, migrating from anterior to posterior, express lower levels of *Fat1* RNA than the surrounding subcutaneous cell layer (pink arrows). Intensity of *Fat1* staining in this subcutaneous layer increases gradually in caudal sections. (**E–H**) Orientation of CM myoblast migration in whole-mounts of E12.5 *Fat1^LacZ/LacZ^* and control embryos detected using *MyoD in situ* hybridization. In all panels anterior is to the left, dorsal is to the top. (**E**) The CM muscle (purple dotted line) in *Fat1^LacZ/LacZ^* embryos displays reduced size and altered shape as compared to wild type. Higher magnification images (right hand panels) show that within the CM muscle, radial organization of myoblast chains was perturbed by *Fat1*-deficiency, resulting in a fuzzy migration front and irregular distribution of myoblasts (red arrows). In addition, ectopic clusters of myoblasts (orange arrows) are detected in the shoulder area (dotted orange line). (**F**) Quantification of the abnormal orientation of *Fat1* mutant myoblasts. The angle between the longest diameter of each myoblast nucleus and the axis of the closest myoblast chain was measured on flat-mounted CM muscles. The bar graph presents mean (± s.e.m.) percentages of *myoD* ^+^ nuclei displaying a given angle (by angle ranges of 10°) for wild type (gray) and *Fat1^LacZ/LacZ^* (black) embryos. (**G, H**) High magnification images of *MyoD*-expressing myoblasts in equivalent positions – within the chains (**G**) or at the leading edge (migration front, **H**) – in the CM of mutants and controls. Scale bars: (**A–C**), 0.8 mm; (**D**) 300 µm; (**E**), left: 0.5 mm; (**E**), right: 100 µm; (**G, H**) 10 µm.

In search of mechanisms that control muscle position and form, we studied *Fat1* expression at stages of muscle morphogenesis. We chose first to study a muscle with a characteristic fan-shaped form, the subcutaneous muscle *cutaneous maximus* (CM). During embryogenesis, following delamination from the dermomyotomal lip at forelimb levels, CM precursors, identified through their specific expression of GDNF, reach the base of the limb, turn, and spread under the skin in a radial manner [39]–[40] (Figure 1A). This migration pattern reflects collective and polarized cell migration, visible owing to expression of the *MLC3F^2E^* reporter line or of the muscle fate marker MyoD, through the formation of chains of myoblasts aligned in radial directions (Figure 1B and 1E top right panel). At the stages of CM migration, whole mount X-gal staining in embryos carrying a *LacZ* reporter gene-trap insertion in the mouse *Fat1* gene revealed a hot-spot of *Fat1* expression highlighting the migration area (Figure 1C, Figure S1). We found that CM myoblasts express *Fat1* RNA and appear to be positioned in a subcutaneous layer which itself expresses *Fat1* RNA, this surrounding subcutaneous tissue displaying a rostrocaudal gradient of intensity, with highest intensity caudal to the extremity of the CM (Figure 1C; D). Thus, CM myoblasts express *Fat1* and appear to migrate along an increasing gradient of *Fat1* expression.

**Figure 2:**
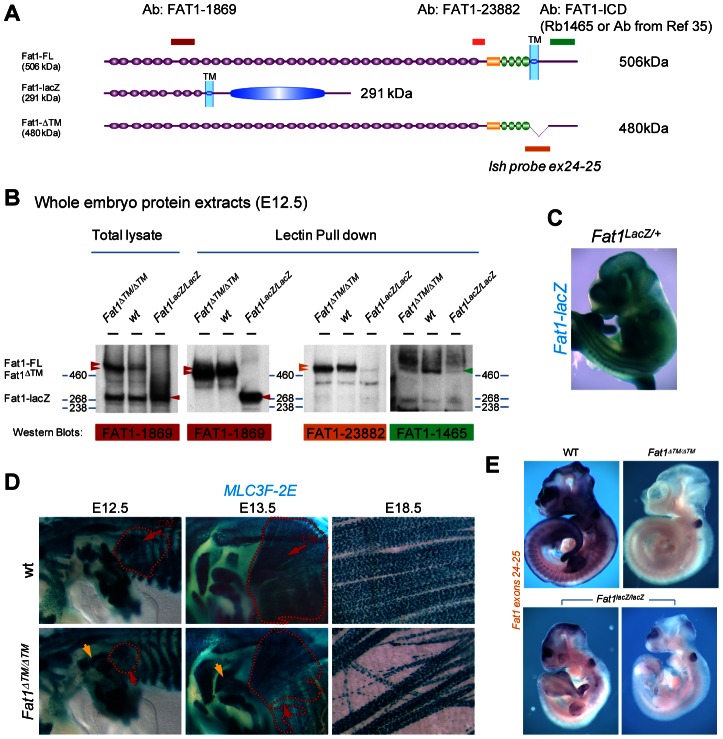
The transmembrane domain of FAT1 is required to polarize muscle migration. (**A**) Schemes representing the main protein product expected from a wild type, a *Fat1^LacZ^*, and a *Fat1^ΔTM^* locus. Positions of the epitopes for three antibodies are also shown, with a color code matching that used in the western blots below. (**B**) Western blot analysis of the FAT1 protein products observed in total lysates from E12.5 *Fat1^LacZ/LacZ^*, wild type, and *Fat1^ΔTM/ΔTM^* embryos using indicated antibodies, which targeted epitopes are positioned in (**A**). (**C**) Whole mount LacZ staining of E12.5 *Fat1^LacZ/LacZ^* mutant embryo. (**D**) Skeletal muscle groups were visualized in E12.5, E13.5, and E18.5 control and *Fat1^ΔTM/ΔTM^* embryos carrying the MLC3f-2E transgene, by X-gal staining. Whole mount analysis of skeletal muscles confirms the presence of a reduced CM (red dotted lines) at E12.5, leading to a misshaped CM one day later (E13.5), and the systematic presence of ectopic muscles in the shoulder area (yellow arrow), most frequently inserting between the deltoid and triceps muscles. Flat mounted preparations of the CM dissected from an E18.5 *Fat1^ΔTM/ΔTM^* embryo, showing the reduced density as well as randomly oriented multinucleated myofibres (right panels). (**E**) Whole mount in situ hybridization on E10.5 embryos with an RNA probe matching the Floxed exons (exons 24–25, the probe is indicated in yellow in Figure S4A). The profile of *Fat1* RNA expression in a wild type embryo matches previously reported expression domain, including staining in the limb, somites, branchial arches, telencephalon, midbrain, eye, tail bud, and neural tube roof plate. *Fat1^ΔTM/ΔTM^* embryos are entirely devoid of staining, apart from the otic vesicle, a known site of substrate trapping (yielding background staining). In contrast, varying amounts of residual RNA were consistently observed in *Fat1^LacZ/LacZ^* embryos, in the telencephalon, midbrain, limbs, tailbud, and somites. Two examples are shown with different RNA levels detected.

We therefore asked whether *Fat1* was required for CM location and/or form. We first took advantage of a mouse model carrying a gene-trap insertion in the mouse *Fat1* gene [41]–[42] (allele referred to as *Fat1^LacZ^*). Initial differentiation along the muscle lineage was unaffected in *Fat1^LacZ/LacZ^* embryos since CM myoblasts retained expression of broadly-expressed markers such as *MyoD* (n = 6), and markers of subsets of myoblasts (such as *Six1* (n = 2), *gdnf* (n = 2), and *Lbx1* (n = 2); data not shown). This allowed us to use *MyoD* expression to monitor precursor migration in *Fat1* mutants. In E12.5 *Fat1^LacZ/LacZ^* embryos, we observed 1) an aberrant morphology of the CM muscle, reduced in size, and with ill-defined anterior limits (Figure 1E), 2) a dispersion of migrating myoblasts not only within the CM but also in ectopic areas traditionally devoid of muscle cells. In the CM, higher magnification observations revealed that migration myoblasts failed to show a preferential alignment of their nuclei into migratory chains (Figure 1E–H). This phenotype was associated with morphological changes in individual myoblasts, such as the loss of long cytoplasmic protrusions extending from the leading edge and rounded morphology of some nuclei within the chains (Figure 1G, H). In further support of a role for *Fat1* in migration polarity, numerous clusters of ectopic myoblasts or disoriented single myoblasts were found in the shoulder region of E12.5 mutants, either in ectopic places, or within additional shoulder muscles such as the spinotrapezius muscle (Figure 1E orange arrowheads in orange dotted area; Figure S2, red arrows).

### Regulation of myoblast polarity requires FAT1 transmembrane domain

Further genetic evidence of such a function of FAT1 in control of muscle shape was obtained with another targeted allele of the *Fat1* locus, which we engineered by flanking two exons, 24 and 25, the latter containing the transmembrane domain, with LoxP sites (Figure S3A, targeted allele referred to as *Fat1^Fln^*). Crossing of mice carrying the conditional *Fat1^Fln^* allele with a ubiquitous CRE-expressing mouse line produced, by germline excision of the floxed exons, a constitutively recombined allele, *Fat1^ΔTM^*, which encodes FAT1 protein isoforms lacking the corresponding transmembrane domain (Figure 2A,B). Analysis of myogenic differentiation by in situ hybridization with a *myoD* probe indicated that *Fat1^ΔTM/ΔTM^* embryos exhibited phenotypes identical to those seen in *Fat1^LacZ/LacZ^* embryos (data not shown). This new allele also allowed studying later steps of muscle differentiation by crossing *Fat1^ΔTM^* mice with a transgenic line in which nls-LacZ reporter activity is driven by an enhancer from the *mlc3f* gene (MLC3F-2E) [43]. Expression of this transgene (MLC3F-2E:LacZ) is detected slightly later than *myoD* expression as it reflects differentiation in myocytes and sarcomere assembly [43], hence it allows visualising muscle shapes, but not migrating myoblasts. MLC3f-2E expression in *Fat1^ΔTM/ΔTM^* embryos revealed again the altered morphology of the CM muscle, with missoriented chains of myocytes in the ventral/pectoral half of the CM and shoulder belt muscles (Figure 2D, and Figure S3B). Furthermore, *Fat1^ΔTM/ΔTM^* embryos were found to exhibit an extra muscle ectopically located in the shoulder area (Figure 2D). Finally, we also visualized multinucleated myofibres owing to the nuclear β-galactosidase staining at late gestation stages, and confirmed the persistence of misoriented myofibers in the mature CM muscle of *Fat1^ΔTM/ΔTM^* E18.5/P0 embryos (Figure 2D). Taken together, our data show that *Fat1* is required to control the shape and position of subsets of migratory muscles in the developing embryo, by controlling coordinated polarity of collectively migrating myoblasts.

### The developmental map of *Fat1*-dependent muscles

**Figure 3:**
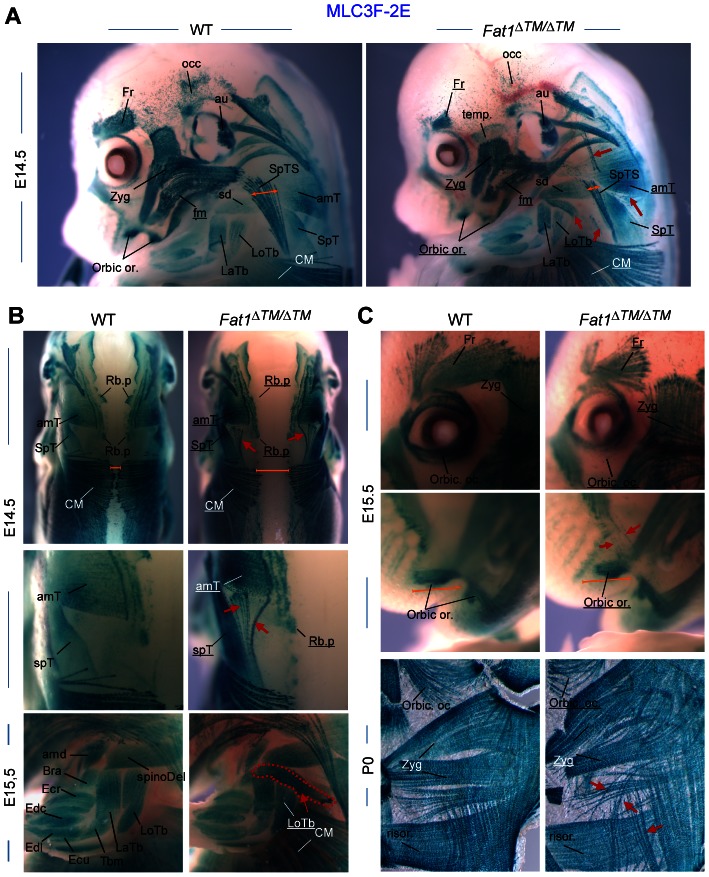
*Fat1* loss of function alters shapes of selective facial and scapulohumeral muscles. Skeletal muscle groups were visualized in E14.5, E15.5, and E18.5 wild type and *Fat1^ΔTM/ΔTM^* embryos carrying the MLC3f-2E (LacZ) transgene, by X-gal staining. (**A**) overview of the face and forelimb musculature at E14.5. Overall, constitutive ablation of *Fat1* causes developmental abnormalities of muscle shape, affecting selective subcutaneous muscles in the face (Zyg. Min and Zyg maj, muscles, Occip. F, orbic. Or. and temporalis Muscles) and selective muscles in the scapulohumeral region. Muscle names are indicated. Muscles which are reduced or show an altered shape have their name underlined in *Fat1^ΔTM/ΔTM^* mutant pictures. Ectopic muscles are indicated with red arrows. (**B**) Muscles of the scapulohumeral area at E14.5 and E15.5, visualized with dorsal views of the scapular muscles at E14.5, and side views of the forelimb at E15.5. Dorsal views reveal the reduced extent of the CM and Rhomboid muscles, and the abnormal connections between the upper and lower parts of the trapezius (amT and spT, respectively). A large additional ectopic muscle (red dotted line, bottom picture) is observed in *Fat1^ΔTM/ΔTM^* embryo, that appears ectopically inserted between the spinodeltoid and Triceps brachii (LoTB and LaTb) muscles. (**C**) Analysis of muscles in the face at E14.5 (**A**), E15.5 (**C**, top), and at P0 (**C**, bottom), reveals abnormalities in shape, myofibre orientation and density in several subcutaneous muscles (red arrows) that occupy positions equivalent to that of human muscles of facial expression, while deeper muscles such as the masseters (see Figure 6D and data not shown) display normal shape. Overall the topography of muscles affected in *Fat1* mutant mice resembles the map of muscles affected in human FSHD muscle in early phases of the disease. Muscle names abbreviations: amT: acromiotrapezius; amd: acromiodeltoid; Bra: brachialis; CM: cutaneous maximus; Ecu: Extensor carpi ulnaris; Ecr: Extensor carpi radialis; Edc: Extensor digitorum communis; Edl: extensor digitorum longus; Fr: Frontalis; LaTb: lateral Triceps Brachii; LoTb: Longitudinal Triceps Brachii; Occ: occipitalis; Orbic. Oc: orbicularis oculis; Orbic Or: Orbicularis Oris; Risor: Risorius (position equivalent to that of Risorius in human); SpD: spinodeltoid; SpT: spinotrapezius; SpTS: Subcutaneous part of the Spinotrapezius muscle; Temp: Temporo-parietal muscle; Zyg: Zygomaticus (position inferred from equivalent position in human).

We next wished to extend our description of the map of *Fat1*-dependent muscles by exploring the phenotypes exhibited by *Fat1^ΔTM/ΔTM^* embryos carrying the MLC3F-2E transgene at later developmental stages (E14.5 and E15.5), when migration has been completed and muscle shapes are determined. In the scapulohumeral area of all *Fat1^ΔTM/ΔTM^*;*MLC3F-2E* embryos examined, we consistently observed an extra muscle in a stereotyped ectopic position, systematically attached between the spinodeltoid muscle and the triceps brachii muscles (Figure 3A,B). Just dorsal to the spinodeltoid, we found a subcutaneous portion of the spinotrapezius muscle (SpTS) to be drastically reduced in *Fat1^ΔTM/ΔTM^*;*MLC3F-2E* embryos (Figure 3A, orange arrows). Observation from a dorsal point of view reveals that midline junction of the CM muscle and of Rhomboid muscles (Rh) is delayed, so that a large gap is seen in the back of an E14.5 *Fat1^ΔTM/ΔTM^* embryo (Figure 3B, orange line). Numerous mispositionned myofibres create ectopic bridges between the acromiotrapezius and spinotrapezius muscles in *Fat1^ΔTM/ΔTM^*;*MLC3F-2E* embryos (Figure 3B; read arrows in top and middle picture). Analysis of muscles in the face at E14.5, E15.5, and at P0, reveals abnormalities in shape, myofibre orientation, and density in several subcutaneous muscles in the facial skin (Figure 3C, red arrows) that occupy positions reminiscent of the position of human muscles of facial expression. The flat structure of these subcutaneous muscles is analogous to that of the CM muscle, and the alterations observed in *Fat1^ΔTM/ΔTM^* neonates also include random orientation of multinucleated myofibres (Figure 3C). In contrast, deeper muscles such as the masseters display normal shape in *Fat1^ΔTM/ΔTM^* mutants (see Figure 3C and data not shown). Of notice, although muscle shape defects were found in stereotyped places, their severity was variable, and *Fat1^ΔTM/ΔTM^* embryos were frequently asymmetrically affected (Figure S4, see also Figure S12A). As previously observed in *Fat1^LacZ/LacZ^* mutants, examination of muscle development at E14.5 and E15.5 in *Fat1^ΔTM/ΔTM^* embryos confirmed that *Fat1* loss of function selectively affects muscles of the facial and scapulohumeral ares, and that *Fat1* is not required to shape other migratory muscles such as the diaphragm or hindlimb muscles, which were identical between wild type and *Fat1^ΔTM/ΔTM^* embryos (Figure S4 and data not shown). Overall, in addition to the abnormal shape of the cutaneous maximus muscle, we found that *Fat1* was required to shape selective and stereotyped groups of muscles in the scapulohumeral interface, as well as subcutaneous muscles of the face.

### Mice with reduced *Fat1* expression develop early regionalized muscle wasting restricted to mis-shaped muscles

**Figure 4:**
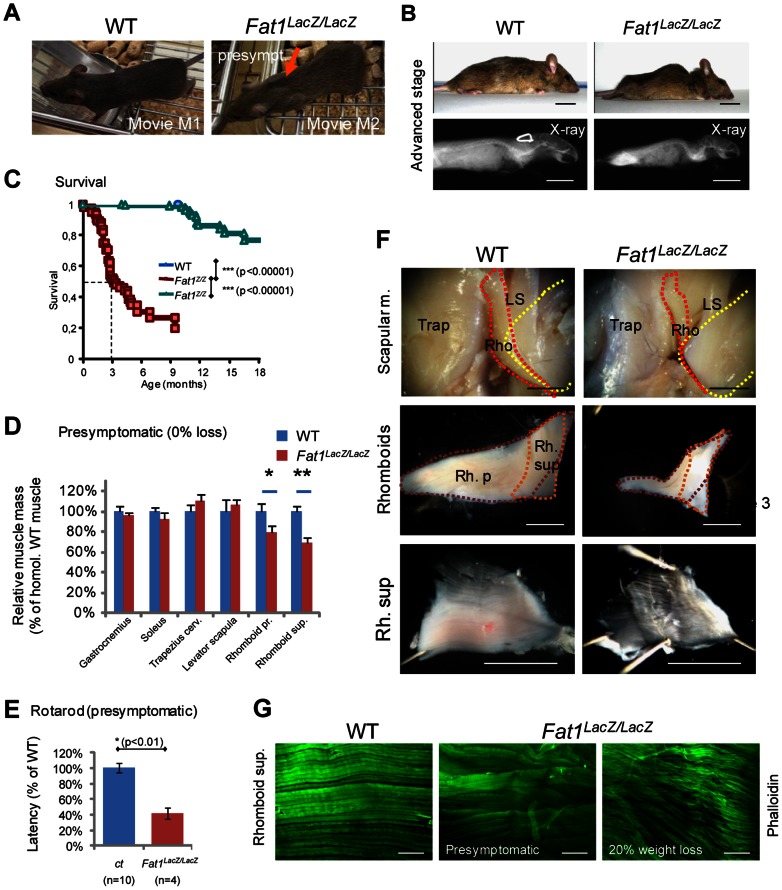
Presymptomatic adult *Fat1* mutant mice show selective defects in scapular muscles. (**A**) Adult *Fat1^LacZ/LacZ^* mice show visible scapular winging (orange arrow) at stages prior to detectable weight loss (defined as presymptomatic). Pictures (extracted from movies) show a posture in which the mice challenge their shoulder girdle muscles by extending their head as far rostral as possible. At 7 weeks, wasting of the rhomboid muscles can already be detected in presymptomatic *Fat1^LacZ/LacZ^* mice as they move on a cage grid. Note the large gap (orange arrow) between scapulas (where rhomboids normally maintain scapulas attached to the dorsal spine), not visible in the corresponding position in the wild type littermate. (**B**) At advanced symptomatic stages (30% weight loss, anesthetized mice), there is marked curvature of the spine in the upper back and shoulder area, also visible through X-ray *post-mortem* imaging. (**C**) Kaplan-Meier plot showing survival of wild type, *Fat1^LacZ/+^*, and *Fat1^LacZ/LacZ^* mice. Most *Fat1^LacZ/LacZ^* mice die between 2 and 4 months, with a median survival of 3 months, while a small group survives beyond 6 months. (**D**) Masses of dissected muscles of *Fat1^LacZ/LacZ^* mice at presymptomatic disease stage (0% weight loss, n = 3) relative to age-matched controls (n = 6; average wild type weight defined as 100%). (**E**) Motor performance defects in presymptomatic adult *Fat1^LacZ/LacZ^* mice. Rotarod analysis shows that the latency to fall off from the rod was significantly shorter in presymptomatic adult *Fat1^LacZ/LacZ^*. In this set of experiments, additional *Fat1^LacZ/LacZ^* mice that were symptomatic at the stage when training started had died by the time the test was performed and are therefore not included in the graph. (**F**) Scapular muscle dissection in adult wild type and *Fat1^LacZ/LacZ^* mice reveals a pronounced reduction in volume and thickness of the *rhomboid superficialis* (Rh. Sup.) and *rhomboid profundus* (Rb. P.). This likely underlies the scapular winging phenotype. In the top pictures, the *trapezius cervicalis* (Trap) has been removed on the right side of each mouse to uncover the other scapular muscles (*rhomboids*: Rho; *levator scapula*: LS). Yellow dotted lines indicate the extent of the scapula, red and orange dotted lines that of the two *rhomboid* muscles. The intermediate magnification highlights the respective shapes of the *rhomboid superficialis* (orange dotted line) and *rhomboid profundus* (purple dotted line). (**G**) Phalloidin staining of flat-mounted *rhomboid superficialis* muscles of wild type and *Fat1^LacZ/LacZ^* mice at presymptomatic (middle panel) or advanced disease (20% weight loss; bottom panel) stages shows that early defects of myofiber orientation precede reduction of myofibre diameter. Scale bars: (**F**) 2 mm; (**G**) 300 µm.

We next asked what the consequences of these muscle shape abnormalities were at postnatal stages. Constitutive deletion of *Fat1* was initially shown to lead to neonatal lethality most likely due to defects in kidney filtration [36], [42]. Likewise, constitutive deletion of the transmembrane domain (*Fat1^ΔTM/ΔTM^* mice) also leads to more than 50% lethality at birth, with only a small proportion of mutants surviving to adulthood (Figure S3C). We chose to examine adult *Fat1^LacZ/LacZ^* mutants, since the hypomorphic *Fat1^LacZ^* allele, which results from an insertion of a gene-trap construct in an intron, not deleting any functional domain, allows expression of variable amounts of residual *Fat1* RNA and FAT1 protein in *Fat1^LacZ/LacZ^* mutants (Figure 2E, Figures S5, and S13). This hypomorphic allele, in the genetic background we used, allowed bypassing the neonatal lethality in *Fat1^LacZ/LacZ^* mutants, with more than half the mutant mice surviving after 3 months (Figure 4C), and enabled us to study the postnatal consequences of reduced *Fat1* levels. The variable amounts of residual *Fat1* correlates with the variability in the severity of phenotypes and in the age of death of *Fat1^LacZ/LacZ^* mice. A fraction of these adult phenotypes, in particular the lethality, is likely to result from systemic consequences of kidney phenotype. Indeed, analysis of kidney morphology in the subset of *Fat1^LacZ/LacZ^* mice that exhibited severe weight loss revealed features characteristic of polycystic kidneys, such as cysts formed of enlarged tubules in the cortical renal area (data not shown). Therefore, to score with an objective criterion the progression through adult phenotype stages, body weight was measured for each individual and compared to its own maximal weight [44]. We arbitrarily set the moment a *Fat1^LacZ/LacZ^* mutant mouse has lost 10% of its weight as the visible onset of symptoms associated with kidney malfunction or with other phenotypes likely to have systemic consequences. Mutant mice showing more than 10% loss at the stage of analysis were defined as “symptomatic” (related to generalized symptoms, and not to muscles only), and the degree of severity was recorded as percentage weight loss, while *Fat1^LacZ/LacZ^* mutant mice that did not exhibit any weight loss yet were defined as presymptomatic. Although this threshold of 10% weight loss was defined arbitrarily, and even though we cannot exclude that kidney phenotypes also have systemic consequences earlier than this limit, it is difficult, during symptomatic phase, to attribute a primary cause to the symptoms observed. We therefore focused on the presymptomatic phase for most of our studies of adult muscle, and also chose to exclude from our adult studies mutant mice with an impaired growth curve. While *Fat1^LacZ/LacZ^* mice at symptomatic stages (with 20–30% body weight loss) displayed generalized muscle mass reduction (Figures S6B–C, presymptomatic mutant mice showed scapular winging, whereas lumbar posture and hindlimb function appeared unaffected (Figure 4A). Postural abnormalities affecting the shoulder area, indicating weakness of the muscles involved in scapular movements, can be seen when presymptomatic mice move on a cage grid, especially in situations in which they challenge the shoulder girdle muscles by transferring bodyweight rostrally on their forelimbs. These postural abnormalities were accompanied by functional motor defects evidenced in rotarod assays at presymptomatic stages (Figure 4E). Early symptomatic mice (around the 10% threshold) also showed kyphosis, a curvature of the spine known as a hallmark of muscle wasting in the shoulder girdle (Figure 4D, F), without displaying skeletal abnormalities (Figure 4B, X-ray). Similar observations were made in the small proportion of *Fat1^ΔTM/ΔTM^* mice that survived to adult stages.

We next investigated the pathological basis for the selective postural abnormality of the scapulae at presymptomatic stages. Dissection of individual muscles in presymptomatic *Fat1^LacZ/LacZ^* mice revealed a significant mass reduction for both rhomboid muscles when compared to controls (Figure 4D). As expected from the embryonic defect, a severe reduction in thickness of the CM muscle was also observed, although its subcutaneous location made accurate dissection and therefore mass measurement unfeasible. Defects in myofibre orientation similar to those observed at late embryonic stages were confirmed in CM (Figure S6D and data not shown) and in rhomboid muscles (Figure 4G) at all stages examined. In contrast, masses of muscles with unaltered shape when examined during development (i.e hindlimb muscles such as gastrocnemius or soleus) were also not significantly reduced at presymptomatic stages (Figure 4D, Figure S6B, S7). This argues that persistence in mature muscles of misoriented myofibres resulting from fusion of depolarized myoblasts contributes to the shoulder muscle phenotype in presymptomatic mice, although it does not rule out an additional direct function of *Fat1* in muscle, whose loss may also cause muscle degeneration. Lastly, another consequence of developmental dysgenesis that is likely to contribute to focal muscle wasting is the persistence of ectopic muscles (Figure S7). Such ectopic muscles were found to share tendon attachment sites with existing muscles (typically two ipsilateral muscles) including shoulder belt muscles (trapezius, LD, pectoral muscles), and the humeral muscle triceps brachii (Figure S7). This association correlated with a unilateral reduction of the corresponding muscle mass, reduction that nevertheless did not result significant until early symptomatic stages (Figure 4D and data not shown).

**Figure 5:**
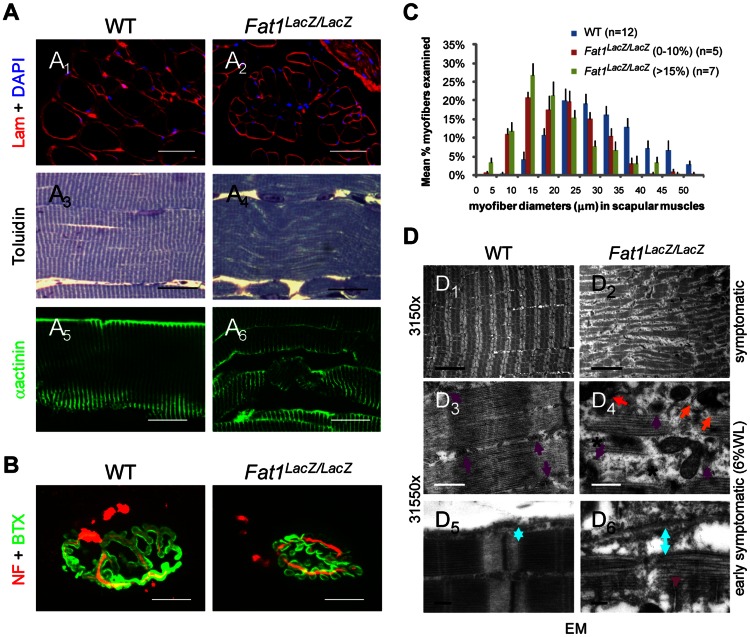
Abnormally shaped shoulder muscles of *Fat1*-deficient mice develop phenotypes involving reduced muscle fibres diameter and structural abnormalities. (**A**) Muscle architecture visualized on transverse (**A**_1,2_) or longitudinal (**A**_3–6_) sections of rhomboid muscles from wild type and *Fat1^LacZ/LacZ^* mice (20% weight loss), using antibodies against laminin and α-actinin, or toluidine blue staining. (**B**) NMJs were visualized by immunolabeling nerve endings with anti-neurofilaments antibodies (NF, red) and AchR clusters with α-bungarotoxin (green). (**C**) Plot of muscle fiber diameter in scapular muscles (*rhomboid*, *trapezius*, *latissimus dorsi*, and *cutaneous maximus*) of adult *Fat1^LacZ/LacZ^* mice at early symptomatic (n = 5, red bars) and advance stages (n = 7, green bars), compared to wild type littermates (n = 12, blue bars). (**D**) Electron micrographs at three different magnifications in *rhomboid muscle* fibres from *Fat1^LacZ/LacZ^* adult mice at early symptomatic stages (6–15% weight loss) show fragmentation of the myofibre architecture and loss of t-tubule integrity. In wild type myofibres, t-tubules (purple arrows) are visible between myofibrils, precisely aligned on either side of each Z-band, at a position coinciding with the end of the myosin filaments. By contrast, in dystrophic fibres from *Fat1^LacZ/LacZ^* mice, the general disorganization correlated with missing (stars), mis-oriented, mis-aligned (orange arrows), or fragmented (red arrows) triads. An increased distance (indicated as blue double arrowed bar) between the sarcolemma and contractile apparatus is observed in *Fat1^LacZ/LacZ^* muscles, compared to wild types, indicating a loss of the tight association between the contractile apparatus and the sarcolemma. Scale bars: (**A_1–2_**) 50 µm; (**A_3–6_**) 20 µm; (**B**) 15 µm; (**D_1,2_**) 5 µm; (**D_3,4_**) 0.5 µm; (**D_5,6_**) 0.2 µm.

The phenotypes resulting from developmental dysgenesis were not restricted to muscle shape and mass. Histological analyses revealed that a significant reduction in fibre diameter was detectable already at early symptomatic stages in those muscles in which we detected developmental defects, including the CM, Rhomboids (Figure 4G, superior and profundis), and Trapezius muscle (Figure 5C, pooled analysis). This was also true for *Fat1^ΔTM/ΔTM^* mice analysed at presymptomatic stages (Figure S8). In contrast, at presymptomatic stages, analysis of myofiber diameters in muscles whose shape was unaffected at developmental stages (such as gastrocnemius or soleus, and also diaphragm) revealed no significant abnormality as compared to control mice (Figure 4D, Figure S6B, and data not shown). In affected muscles (trapezius, rhomboid, Pectoralis Major, LD, and CM), we observed a range of additional abnormalities including inflammatory infiltrations between myofibres, most frequently perivascular, in both presymptomatic *Fat1^LacZ/LacZ^* and *Fat1^ΔTM/ΔTM^* mice (Figure S6D and Figure S7). Fibre necrosis was also observed at more advanced symptomatic stages (beyond 10% weight loss, Figure S7L and data not shown), but as mentioned earlier, it is impossible to distinguish whether any abnormality at symptomatic stage is strictly related to muscle defects, or reflects systemic consequences of unrelated phenotypes. Finally, observation of myofibre structure in affected muscles (trapezius, rhomboid, Pectoralis Major, LD, and CM) revealed progressive disruption of higher level organization, with appearance at presymptomatic stages of multiple faults disrupting the regular alignment of sarcomeric structures (Figure 5A, D), and the detachment of the sarcolemma from the contractile apparatus (Figure 5D). Overall, alterations of muscle integrity at pre-symptomatic stages were only detected in those muscles in which we reported fully penetrant myoblast or myofibre orientation defects (CM, Rhomboids, and Tapezius). Analysis of neuromuscular junctions in affected shoulder muscles also revealed a proportion of junctions showing fragmentation (Figure 5B), denervation, and atrophy (Figure S9). Such defects did not reflect a primary failure of NMJ innervations, as all neuromuscular junctions observed at early postnatal stages (P3) were indistinguishable from wild type (data not shown). Nevertheless, although the muscles that were spared during development and at presymptomatic stages (e.g gastrocnemius, soleus, masseters) were seen to harbour histological signs of muscle atrophy (evenly reduced myofiber diameter) at advanced symptomatic stages (Figure S6B), we did not observe muscle degeneration, inflammation, necrosis, or fragmentation of the contractile apparatus (data not shown). These results are consistent with the possibility that the developmental abnormalities of muscle shape constitute a topographic frame in which muscles might be predisposed to undergo early onset muscle wasting, prior to the appearance of systemic consequences of non-muscle phenotypes and the concomitant generalization of muscle wasting. These findings do not exclude however the possibility that *Fat1* may play additional roles during muscle biology other than controlling shape during development.

### Ablation of *Fat1* functions in premigratory myoblasts with *Pax3-cre* is sufficient to alter muscle shape

**Figure 6:**
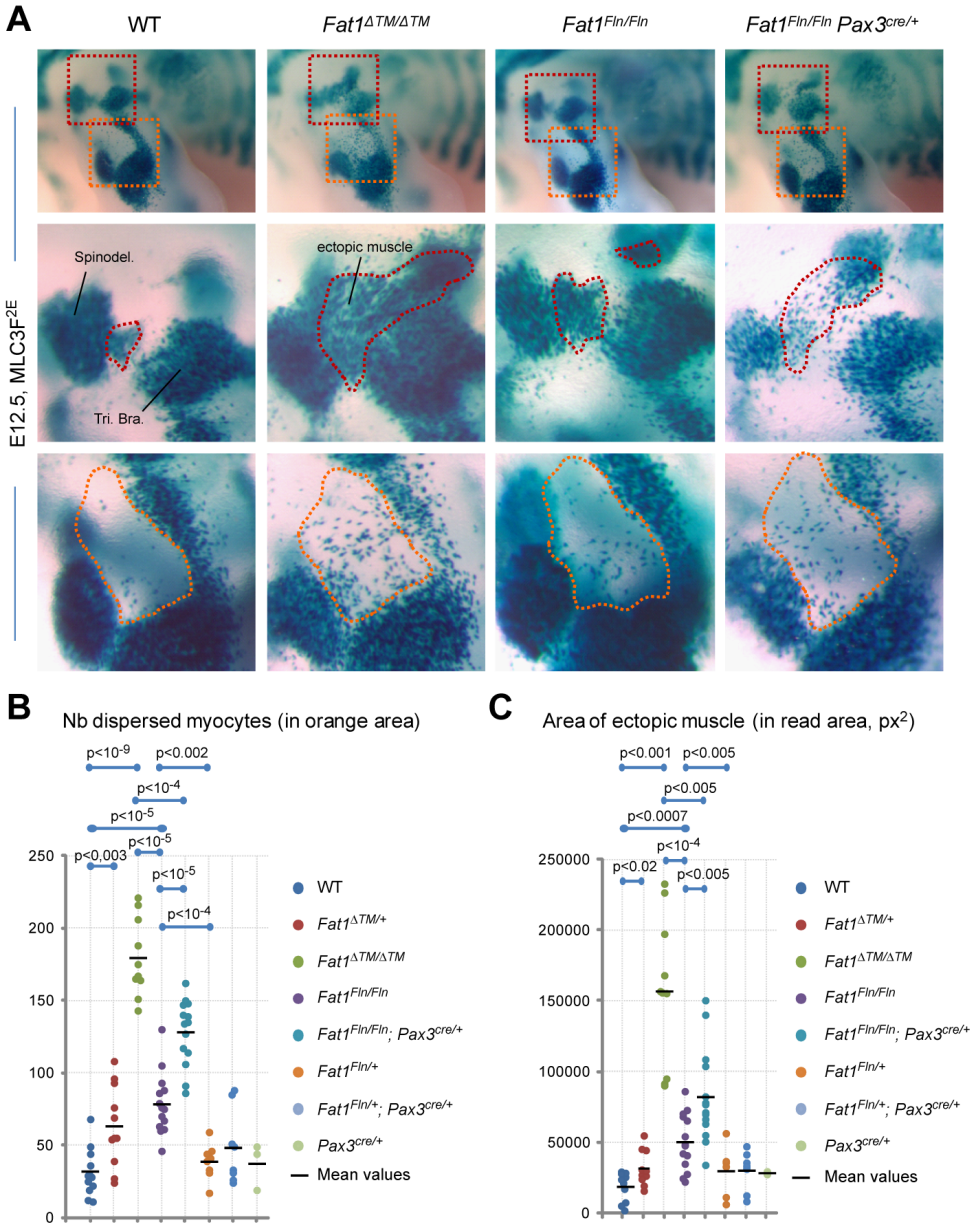
Ablation of *Fat1* in premigratory myoblasts using *Pax3-cre* partially reproduces the muscle migration/shape abnormalities of the constitutive knockout. (**A**) Skeletal muscle cells were visualized at E12.5 in WT, *Fat1^ΔTM/ΔTM^*, *Fat1^Fln/Fln^*, and *Fat1^Fln/Fln^*; *Pax3^cre/+^* embryos, owing to the MLC3F-2E transgene by performing X-gal staining, after clearing in 100% glycerol. The upper panels show micrographs of the forelimb area, and indicate the positions at which higher magnification pictures shown in the two lower panels were taken. (**B, C**) The phenotype was quantified in WT, *Fat1^ΔTM/ΔTM^*, *Fat1^Fln/Fln^*, and *Fat1^Fln/Fln^*; *Pax3^cre/+^* as well as in the control genotypes in *Fat1^ΔTM/+^*, *Fat1^Fln/+^* and *Fat1^Fln/+^*; *Pax3^cre/+^* and *Pax3^cre/+^* in two different manners: (**B**) by counting the number of dispersed myocytes found in the elbow area (orange dotted lines in the lower panels in (**A**)), (**C**) by measuring the area occupied by the ectopically positioned muscle (or myocyte cluster) that appears inserted between (red dotted line in middle panels). All data from a given genotype are plotted on a vertical line. Overlapping dots were arbitrarily moved away from the vertical lines to allow showing all results distinctly. In both cases, the *Fat1^ΔTM/ΔTM^*, *Fat1^Fln/Fln^*, and *Fat1^Fln/Fln^*; *Pax3^cre/+^* groups were each significantly different from the control genotypes (WT, *Fat1^Fln/+^*, and *Fat1^Fln/+^*; *Pax3^cre/+^* respectively, t-test, p values indicated), and were significantly different from each other (*Fat1^ΔTM/ΔTM^* from *Fat1^Fln/Fln^*, and from *Fat1^Fln/Fln^*; *Pax3^cre/+^*, but also *Fat1^Fln/Fln^* from *Fat1^Fln/Fln^*;*Pax3^cre/+^*, t-test, p values indicated).

We next asked if the function of *Fat1* in shaping facioscapulohumeral muscles was exerted cell-autonomously in migrating muscle precursors. In order to perform tissue-specific ablation of *Fat1* in muscles at a stage compatible with migration, we reasoned that transgenic lines in which CRE expression would reproduce that of genes of the muscle differentiation cascade, such as *myoD* or *Myf5*, would occur too late to have an impact on the migration itself. Therefore, to ablate *Fat1* exons 24 and 25 in premigratory myoblasts, we took advantage of the *Pax3-cre* knock-in line [45] (Figure S10). Our conditional allele of *Fat1* (*Fat1^Fln^*) initially includes the neo cassette that was used to engineer the mouse model. Although presence the neo cassette caused mild lowering of *Fat1* expression levels (Figure S11), this only resulted in subtle, although statistically significant, morphological defects in *Fat1^Fln/Fln^* embryos/mice compared to controls (Figure 6 and Figure S12). This allowed using the *Fat1^Fln/Fln^* mutants for conditional studies with tissue-specific CRE lines, without requiring Flp/FRT recombination to further ablate the neo cassette. We therefore compared muscle development in *Fat1^Fln/Fln^*;*Pax3^cre/+^* and *Fat1^Fln/Fln^* embryos, taking advantage of the MLC3F-2E transgene 1) to visualize the shape of every muscle and 2) to quantify the number of muscle cells dispersed in ectopic areas. We followed muscles belonging to *Pax3*-derived territories in the scapulohumeral area, where ablation of *Fat1* leads to measurable phenotypes in *Fat1^ΔTM/ΔTM^*;*MLC3F-2E^+^* embryos (Figure 6A). First, we found significantly higher numbers of dispersed myocytes in the forelimb of *Fat1^Fln/Fln^*;*Pax3^cre/+^* embryos than in *Fat1^Fln/Fln^* embryos (Figure 6A, B). Second, an ectopic muscle similar to the one found in *Fat1^ΔTM/ΔTM^* embryos could be measured in *Fat1^Fln/Fln^*;*Pax3^cre/+^* embryos, and its surface was significantly larger than in *Fat1^Fln/Fln^* embryos (Figure 6A, C). At later developmental stages, in addition to confirming the persistence and position of this ectopic muscle in *Fat1^Fln/Fln^*;*Pax3^cre/+^* embryos, as in *Fat1^ΔTM/ΔTM^*; *MLC3F-2E^+^* embryo. Furthermore we also detected a reduced density of myofibers in the CM muscle and in the subcutaneous part of the spinotrapezoid muscle (Figure S12). As the *Pax3^cre/+^* line is a CRE knock-in, but also a knock-out of the endogenous *Pax3* locus, the resulting loss of one copy of *Pax3* may be in itself sufficient to enhance FAT1-dependent phenotypes. To rule this out, we have evaluated the effect of combining a *Pax3^cre/+^* context to the recombined *Fat1^ΔTM^* allele, and found no enhanced phenotype in either *Fat1^ΔTM/+^*:*Pax3^cre/+^* or *Fat1^ΔTM/ΔTM^*:*Pax3^cre/+^* embryos compared to *Fat1^ΔTM/+^* or *Fat1^ΔTM/ΔTM^* embryos, respectively (data not shown). Finally, *Fat1^Fln/Fln^*;*Pax3^cre/+^* embryos did not display significantly more abnormalities in the subcutaneous facial muscles or in the spinotrapezius muscle than the mild phenotypes observed in *Fat1^Fln/Fln^* embryos (Figure S12), consistent with the fact that facial muscles do not belong to the Pax3-CRE lineage [46]. Furthermore, if ablation in facial neural crest cells, driven by Pax3-CRE activity, had been responsible for altering muscle shape, it would have done so as efficiently in facial muscles as in trunk muscles. The lack of enhancement of facial muscle phenotypes in *Fat1^Fln/Fln^*;*Pax3^cre/+^* compared to *Fat1^Fln/Fln^* embryos thereby also excludes a contributing role of *Fat1* expression in neural crest-derived cells. Thus ablating *Fat1* in *Pax3*-derived cells is sufficient to partially reproduce the defects observed in scapulohumeral muscles of the constitutive *Fat1* mutants, indicating that *Fat1* is required cell-autonomously in migrating myoblasts to control the polarity of their migration.

### Later FAT1 expression in differentiated muscles

**Figure 7:**
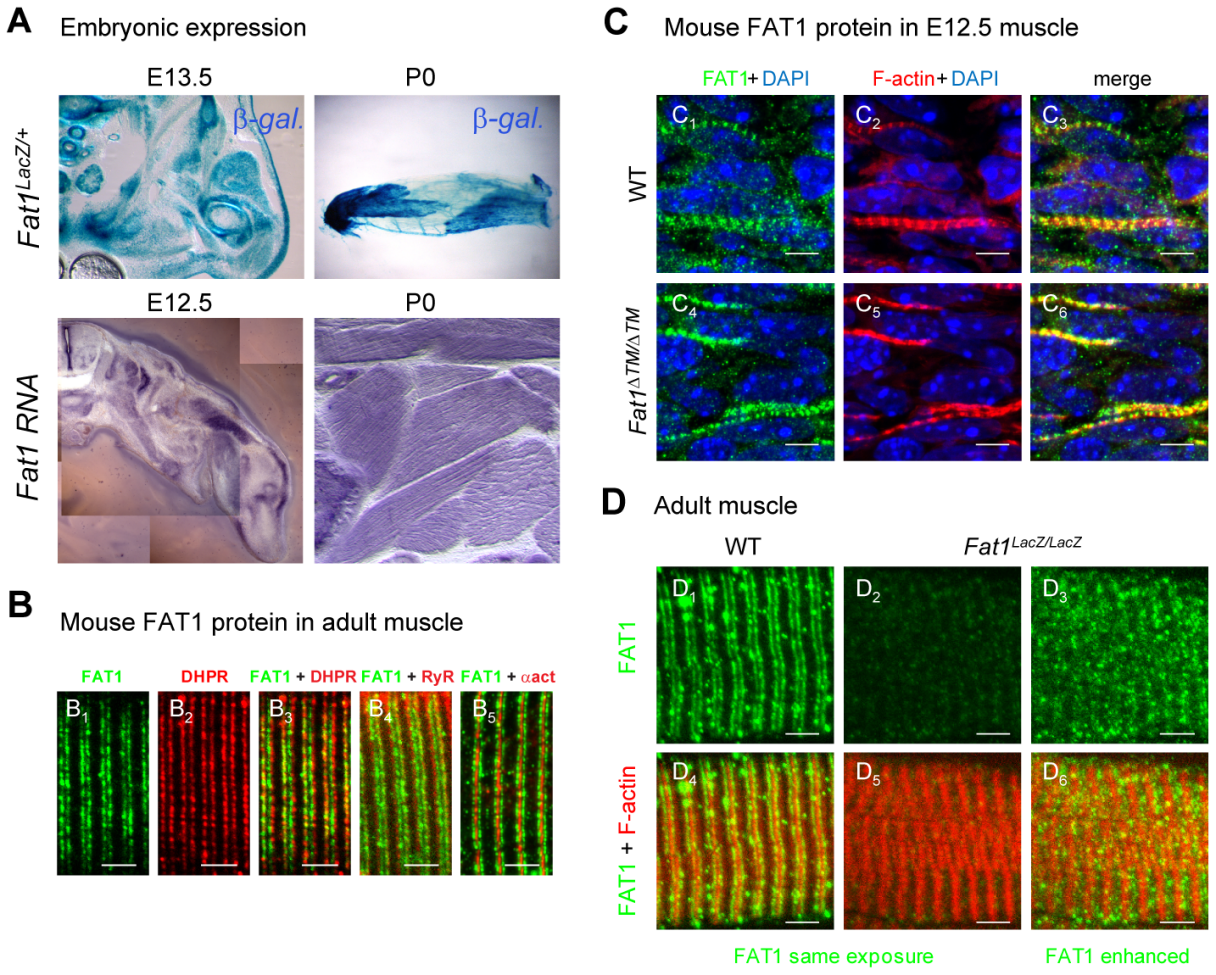
*Fat1* expression at late stages of muscle differentiation. (**A**) *Fat1* expression was visualized in E13.5 embryos or in neonate (P0) muscle by β-galactosidase staining or by in situ hybridization with a *Fat1* 3′UTR RNA probe. (**B**–**D**) Immunolocalization of FAT1 (anti-FAT1-ICD from [35], green) was performed in E12.5 mouse embryo (**C**), and on adult (**B, D**) muscle fibers on longitudinal muscle cryosections from wild type (**B**, **C_1–3_, D_1,4_**), from *Fat1^ΔTM/ΔTM^* embryos (**C_4–6_**), and from *Fat1^LacZ/LacZ^* (**D_2–3_, D_5–6_)** mice, combined with either antibodies against alpha-actinin (red, **B_5_**), DHPR (Cacna1s) (red, **B_2,3_**), or RyR (red, **B_4_**), or with Phalloidin (red, **C, D**). In **D**, Green channel images (FAT1) were first captured with either identical exposure time between wild type and mutants (**D_1,4_** and **D_2,5_**, 421 ms), or with longer exposure time (**D_3,6_**, 2222 ms). This indicates that the epitope detected by the anti-FAT1-ICD antibody (from ref [35]) is present in reduced but detectable amounts in *Fat1^LacZ/LacZ^* muscles. This observation was made when *Fat1^LacZ/LacZ^* mice (n = 2 at P0; and n = 3 at adult stages) displayed severe muscle defects at the stage of dissection, indicating that levels of FAT1 protein inversely correlate with phenotype severity. Scale bars: (**B**–**D**) 4 µm, (**C**) 6 µm.

As we asked whether in addition to the control of muscle migration, *Fat1* may play additional roles in mature muscle, we noticed that in mouse, *Fat1* is also expressed in differentiated muscle fibres after migration stages. This expression can be detected through the pattern of β-galactosidase expression in *Fat1^LacZ/+^* embryos, and by in situ hybridization (Figure 7A). Furthermore antibodies against FAT1 C-terminal cytoplasmic tail detected a protein localized in stripes within muscle fibres (Figure 7B–D), on either side of alpha-actinin-positive sarcomere boundaries (so called Z-bands, Figure 7B). In adult mouse muscle, the stripes of FAT1 protein are closely juxtaposed with DHPR, a calcium channel present in transverse (t)-tubules [47] (Figure 7B). Such localization is consistent with *Fat1* also playing a direct role in muscle biology, distinct from its early function in orienting myoblast polarity. Consistent with previous reports showing that cytoplasmic variants in FAT1 proteins exhibit distinct subcellular localisation [48], and that the cytoplasmic domain can translocate in the nucleus [49], another antibody directed against the cytoplasmic domain (FAT1-1465 antibody) also detected FAT1 protein in significant proportion of nuclei in adult mouse muscle fibres (data not shown). Western blot analyses indicated that a full length FAT1 protein is only detected in whole embryo extracts (at E12.5, Figure 2B) or in isolated brain tissue, but not in muscle tissue, where the most abundant bands detected with anti-FAT1-ICD antibodies were smaller molecular weight proteins (Figure S13), which production is spared by the genetic alterations in both *Fat1^LacZ/LacZ^* and *Fat1^ΔTM/ΔTM^* mutants (Figure 7C,D, Figure S5, S11, S13 and data not shown). While some of these smaller isoforms might be cleavage products of full length FAT1 [50]–[52], additional short isoforms are also consistent with gene products resulting from transcript initiation at alternative downstream promoters, as proposed by genome browsers (Ensembl, UCSC; Figure S5A, with EST-based genes referenced in NCBIM37 mouse genome and in GRCh37 human genome assemblies). Neither the gene trap insertion after the first exon (this study), nor the removal of the entire first exon (in the published knockout allele [36]), suppress such gene products. Deletion of the transmembrane domain in *Fat1^ΔTM/ΔTM^* mutants also allowed expression of protein products with unchanged size (Figure S13), although it nevertheless led to a more severe phenotype with drastic neonatal lethality (compare Figure S3C and Figure 4C). Quantitative RT-PCR confirmed the presence of significant amounts of *Fat1* RNA containing the last exons (26 to 28) in *Fat1^ΔTM/ΔTM^* mutants, albeit at reduced levels when compared to wild types (Figure S11). Thus, in the case of all mutant alleles, the remaining smaller isoforms might still carry out *Fat1* functions at least partially, resulting in hypomorphic phenotypes with variable severity. Consistently, in immunohistochemistry experiments on muscle sections, residual FAT1 staining is also observed in myofibres of *Fat1^ΔTM/ΔTM^* mutants and *Fat1^LacZ/LacZ^* mice, and staining intensity in *Fat1^LacZ/LacZ^* mice that survived to adulthood inversely correlated with phenotype severity at the level of individual myofibers (Figure 7C,D and data not shown). Presence of unchanged smaller FAT1 isoforms in muscles of *Fat1^ΔTM/ΔTM^* mutants precludes using this mouse line to investigate their function. However, it indicates that the phenotype of muscle migration is not the consequence of their deletion, but results from ablation (constitutive or driven by *Pax3-cre*) of the transmembrane domain in full length FAT1 proteins that are abundant at developmental stages (Figure 2B).

### *Fat1*-deficient mice present characteristics of FSHD

**Figure 8:**
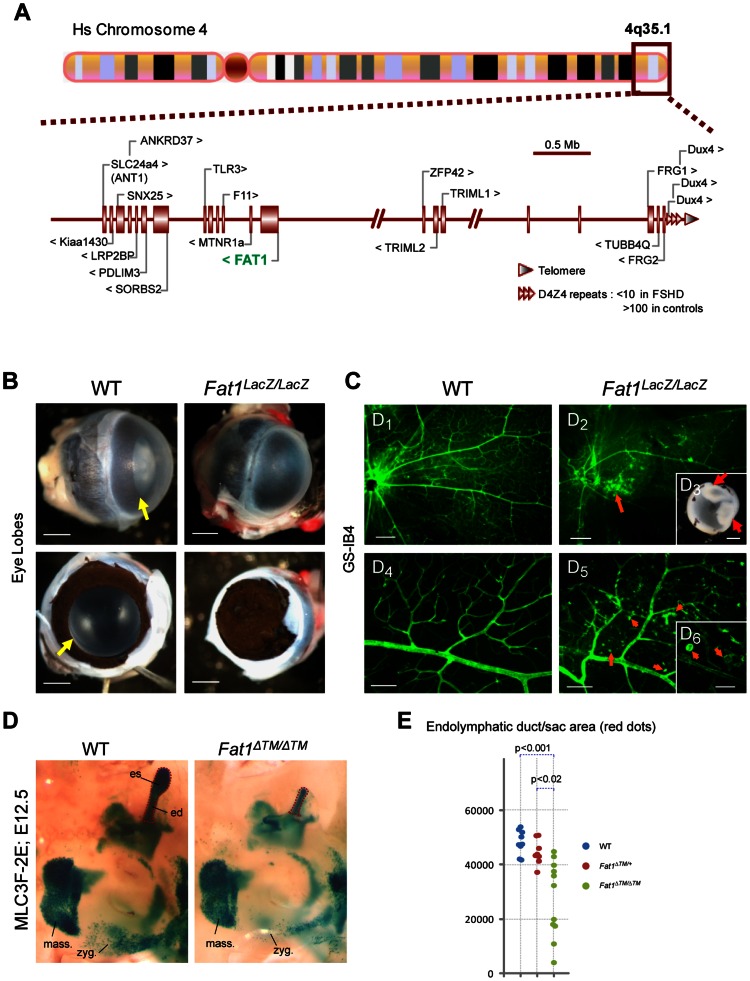
Selective changes in *Fat1* mutant mice recapitulate the clinical picture of FSHD. (**A**) Schematic representation of the human 4q35.2 region, including 5 Mb upstream of the FSHD-associated *D4Z4* repeat array. (**B–C**) Retinal defects and exudative vasculopathy in adult *Fat1^LacZ/LacZ^* retinas. *Fat1^LacZ/LacZ^* eyes have an opaque appearance, in contrast to wild type eyes (**B**; yellow arrow). Removal of the cornea reveals absence of opening of the pigmented retina (aniridia), which therefore covers the lens and prevents light from entering the eye. (**C**) Retinal vasculature visualized using isolectinB4 (GS-IB4) staining of flat-mounted adult retinas from wild type and *Fat1^LacZ/LacZ^* mice. The retina of *Fat1^LacZ/LacZ^* mice displayed zones in which the normal net of secondary and tertiary vessels was replaced by disorganized vasculature, revealing numerous intra-retinal microvascular abnormalities, including IB4-binding microaneurysms (orange arrows). Insert: Example of severe retinal detachment (red arrows) observed in *Fat1^LacZ/LacZ^* eyes, visible even through the lens prior to its removal during dissection. (**D**) The shape of the inner ear was visualized at E12.5 in WT and *Fat1^ΔTM/ΔTM^* embryos owing to the MLC3F-2E transgene, which is expressed in the developing inner ear in addition to differentiating muscles. Micrographs show an area of the face around the ear. This area shows: left: the masserter muscles (unaffected), bottom: a stream of muscle cells migrating subcutaneously from the second brachial arch (future subcutaneous muscles of the face, which migration path is visibly affected); and top right: the inner ear structure with the endolymphatic duct (ed), a long tube oriented dorsally, finishing with an enlarged area called the endolymphatic sac (es). Both the ed and es are reduced in half *Fat1^ΔTM/ΔTM^* inner ears examined (frequently asymmetric). (**E**) Quantification of the inner ear shape defect was performed by measuring the area occupied by the endolymphatic duct (ed) and endolymphatic sac (es), as illustrated with the red dotted lines in (**D**). Each value for a given genotype were plotted on a vertical line, to illustrate the scale of variability of mutant phenotypes. Scale bars: (**B,C_3_**) 0,5 mm; (**C_1–2_**) 200 µm; (**C_4–5_**) 80 µm, (**C_6_**) 30 µm.

Strikingly, the topography of selective alterations in muscle shape that we observed during development in *Fat1* mutant mice closely resembles the map of muscles affected in early phases of human FSHD. Muscle shape abnormalities such as those seen in facial subcutaneous muscles, in trapezius, or in rhomboid muscles are expected to result in lack of facial skin mobility and scapular winging, two symptoms that are frequently the first clinical manifestations of FSHD. The selective muscle weakness observed in presymptomatic *Fat1* mutants in muscles belonging to the developmental map was also reminiscent of the early phase of FSHD. Even at the scale of EM observations, defects in myofibre structure, such as sarcolemma detachment (Figure 5D), included aspects similar to those reported in FSHD biopsies [53]. Finally, asymmetry of muscle symptoms is an important aspect of FSHD symptoms. Asymmetries in muscle shape abnormalities were observed not only in the robust phenotypes displayed by *Fat1^ΔTM/ΔTM^* embryos, but also in the very subtle phenotypes associated with by mild lowering of FAT1 expression in *Fat1^Fln/Fln^* embryos (Figure 6, Figure S12A). In this context, it was interesting to note that the human *FAT1* gene is located at 4q35.2, 3.6 Mb proximal to the *D4Z4* array whose contraction is associated with FSHD (Figure 8A). We therefore asked whether in addition to muscle phenotypes, *Fat1*-deficient mice may also share similarities with non muscular symptoms of FSHD. Besides muscular abnormalities, the phenotypic spectrum of FSHD patients also includes vision defects linked to vascular abnormalities [6], [54]–[55]. As previously reported, constitutive *FAT1* loss-of-function causes abnormalities in eye development, with variable severity and penetrance [36]. The *Fat1^LacZ/LacZ^* mice surviving as adults carried milder phenotypes ranging from residual patterning defects (aniridia, small eye, Figure 8B) to perfectly shaped eyes and retina, in which analysis of vasculature with IB4 or PECAM staining revealed numerous areas with intraretinal telangiectasia, microvascular lesions, micro-aneurysms, and frequent retinal detachments (Figure 8C). Additional non-muscular symptoms associated with FSHD also include high frequency hearing loss, although the cause of these deficits remains underexplored. *Fat1*-deficiency was recently reported [56] to cause mild morphological defects in the inner ear, such as reduced cochlear elongation, and to exacerbate the appearance of ectopic sensory hair caused by loss of FAT4, another FAT-like protocadherin, reflecting their cooperation during in elongation and sensory hair cell patterning in the cochlea [26], [56]–[58]. Furthermore, owing to expression of the MLC3f-2E transgene during inner ear development [59], we observed shortening of the endolymphatic duct and endolymphatic sac in *Fat1^ΔTM/ΔTM^* embryos at E12.5 (7 affected sides out of 12), this shortening being frequently asymmetric (Figure 8D, E). These phenotypes are expected to influence audition. Thus, in addition to the similarity of muscle abnormalities, adult *Fat1* mutant mice also show non-muscular defects reminiscent of clinical symptoms of FSHD. Nevertheless, the severity scale of these phenotypes includes phenotypes more dramatic than those seen in FSHD, and *Fat1*-deficiency also leads to phenotypes such as the previously reported kidney abnormalities, that have no equivalent in FSHD.

### Deregulated *FAT1* expression in human FSHD1 foetal muscles

Considering the gene location and the provocative similarities between *Fat1*-deficiency in mouse and FSHD, we therefore asked whether alterations in *Fat1* expression might be an essential step in the molecular mechanism leading to FSHD pathology in human. As in spite of the essential role of *Fat1* in kidney development, FSHD is not known to be associated with kidney abnormalities, if a mechanism linking FSHD to *Fat1* exists, it is expected to involve partial functional alterations only, such as tissue-specific deregulation of *FAT1* during development. We thus first asked whether in addition to the previously reported gene expression changes [9]–[11], [60], any deregulation of *FAT1* expression levels could be detected in the classical context of FSHD1, in which the pathology is due to the presence of a contracted D4Z4 array on a permissive/pathogenic DUX4-activating context (4qA haplotype) [17]. This possibility was reinforced by the finding that *FAT1* appears to be downregulated by DUX4-fl, but not by DUX4-short in human myoblasts [18]. This result was further validated by qPCR, after lentiviral infection of human myoblasts with DUX4-fl as compared with GFP control (Figure S15D), indicating that DUX4 overexpression is capable of lowering *FAT1* expression in cultured muscle cells. As our results in mice point to the crucial role of *FAT1* deregulation during development, we aimed to analyse *FAT1* expression in rare cases of biopsies from foetuses with a prenatal diagnosis of FSHD1, in spite of the fact that stages of myoblast migration were not accessible to experimentation in this context. Nevertheless, the observation that FAT1 protein is a component of differentiated muscle fibres, enriched in the t-tubule system, is consistent with additional later functions of *FAT1* necessary for muscle integrity.

**Figure 9:**
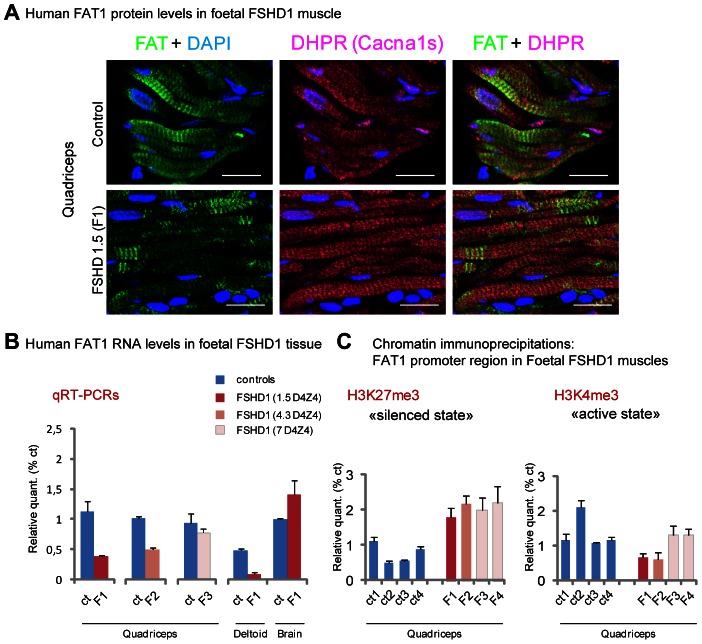
FAT1 protein and RNA levels are mis-regulated in human foetal FSHD tissues. (**A**) Immunolocalization of FAT1 (Rb-1465 anti FAT1-ICD, green) and DHPR (Cacna1s, magenta) in longitudinal sections from human quadriceps biopsies from a control (top) or and FSHD (F1, bottom) foetus with 1.5 D4Z4 repeats. (**B**) qPCR analysis of *FAT1* mRNA levels in quadriceps (3 left graphs) and deltoid muscles (middle graph) and in brain (right graph), comparing respectively with age-matched control foetuses (blue bars), a 26 weeks old FSHD1 foetus (F1) harbouring 1.5 D4Z4 repeats in the 4q35 region (dark red bars), a 16 weeks old FSHD1 foetus harbouring 4.3 D4Z4 repeats at 4q35 region (F2), and twin FSHD1 foetuses aged 28 weeks, with 7 D4Z4 repeats. (**C**) Analysis of the regulatory status of the promoter region by Chromatin immunoprecipitation. The respective level of the following histone marks: H3K27me3 (silenced chromatin; **C-left**), and H3K4m3 (promoter active; **C-right**), in muscle extracts from four age matched controls (ct1 to 4) or four FSHD1 foetuses (F1 to F4) are shown. Relative quantities were normalized with the level of histone marks at the promoter of the *GUSB* gene as internal control, and expressed as % of control 1 (ct1). Scale bars: (**A**) 50 µm.

Possible alterations of *FAT1* expression were therefore assessed in muscle biopsies of human FSHD1 cases at foetal stages through a series of independent approaches. Human FAT1 protein was detected by immunohistochemistry in human muscle biopsies from control foetuses of various stages with antibodies against FAT1 C-terminal cytoplasmic tail, with a striped pattern similar to that seen in mice (Figure 9A, Figure S15). We thus first studied *FAT1* expression levels in tissues from an FSHD1 human foetus carrying a pathogenic 4qA allele harbouring 1.5 D4Z4 copies, expected from previous family history to lead to severe infantile FSHD (Figure S14). Immunocytochemistry with anti-FAT1 antibodies on sections from the quadriceps muscle revealed an overall decrease in FAT1 protein levels compared to quadriceps biospies from control foetuses (Figure 9A), with an irregularly stripped pattern of FAT1 in myofibres that otherwise show a normal distribution of other muscle proteins, such as DHPR. To assess this *FAT1* lowering quantitatively, mRNA expression levels were then followed by qRT-PCR in muscle biopsies from 4 FSHD human foetuses carrying pathogenic 4qA alleles harbouring 1.5, 4.3, and 7 D4Z4 copies (referred to as F1, to F4, respectively; Figure S14A). In F1 foetus, *FAT1* levels were reduced 5-fold in the deltoid (a muscle belonging to the FSHD map) and 3-fold in the quadriceps muscles (a muscle traditionally affected only at late stages in the human disease; Figure 9B). This was also confirmed by Western Blot with anti-FAT1-ICD antibodies (Figure S15A). Additional regulatory changes were detected (Figure S15B), such as an increased level of MURF1 or dysferlin RNAs, while RNA of other muscle components, such as DHPR or γ-Sarcoglycan, were unchanged, ruling out secondary effects of loss of muscle integrity at this stage or quality of the biopsy. In contrast, no significant difference in *FAT1* mRNA levels could be observed in brain when comparing FSHD and control samples from the same foetuses (Figure 9B). Reduction of *FAT1* mRNA levels, albeit to a lesser extent (25% reduction; Figure 9B), and aberrant protein localisation (Figure S15C) were observed in the quadriceps of a second FSHD foetus harbouring 4.3 D4Z4 repeats (F2), from an independent family with previous FSHD history (Figure S14). Finally, no significant quantitative changes were observed in muscle biopsies of twin FSHD foetuses with 7 D4Z4 repeats (Figure 9B), although accumulation of FAT1 protein could be observed in some myofibre nuclei (data not shown), a localization never observed in age matched control biopsies, but reminiscent of adult mouse muscles. In contrast to foetal stages, analysis of *FAT1* mRNA levels in a series of adult FSHD1 biopsies or FSHD-derived myoblasts did not reveal any significant change compared to control biopsies or myoblasts (data not shown), a result consistent with published data [10], [60], or with data available on GEO NCBI. Overall, these results indicate that 1) a reduction of *FAT1* levels in differentiated muscles can be observed is some FSHD1 cases but is not common to all FSHD1 cases at the stages examined; 2) the observed changes in *FAT1* expression levels in FSHD1 occur only during development.

We next asked whether the changes we observed were accompanied with alterations in chromatin state around regulatory sequences of the *FAT1* locus. We thus performed chromatin immunoprecipitations (ChIP) on muscle biopsies derived from these same FSHD1 and control foetuses (Figure 9C), looking for potential changes in the levels of two widely studied chromatin marks: H3K4me3 (trimethylation of histone H3 on lysine 4), a mark of active promoters, and H3K27me3 (trimethylation of histone H3 on lysine 27), which marks transcriptionally silent chromatin [61]–[62]. Consistent with RT-PCR data, we observed a significant decrease in the level of H3K4me3 decorating the *FAT1* promoter region in the two FSHDs foetuses with less than 5 repeats, but not in the foetuses with 7 repeats, as compared to 4 control muscle biopsies of similar age range (Figure 9C right). However, all 4 FSHD1 foetuses nevertheless showed a significant increase in H3K27me3 levels (Figure 9C left). These data are consistent with a switch in chromatin conformation towards the silenced state in the same FSHD1 samples in which RNA levels were reduced, a switch that has the potential to account for a large part of the observed decrease in *FAT1* levels.

### CGH-based identification of contraction-independent FSHD cases carrying deletions of an intronic regulatory element of *FAT1*

**Figure 10:**
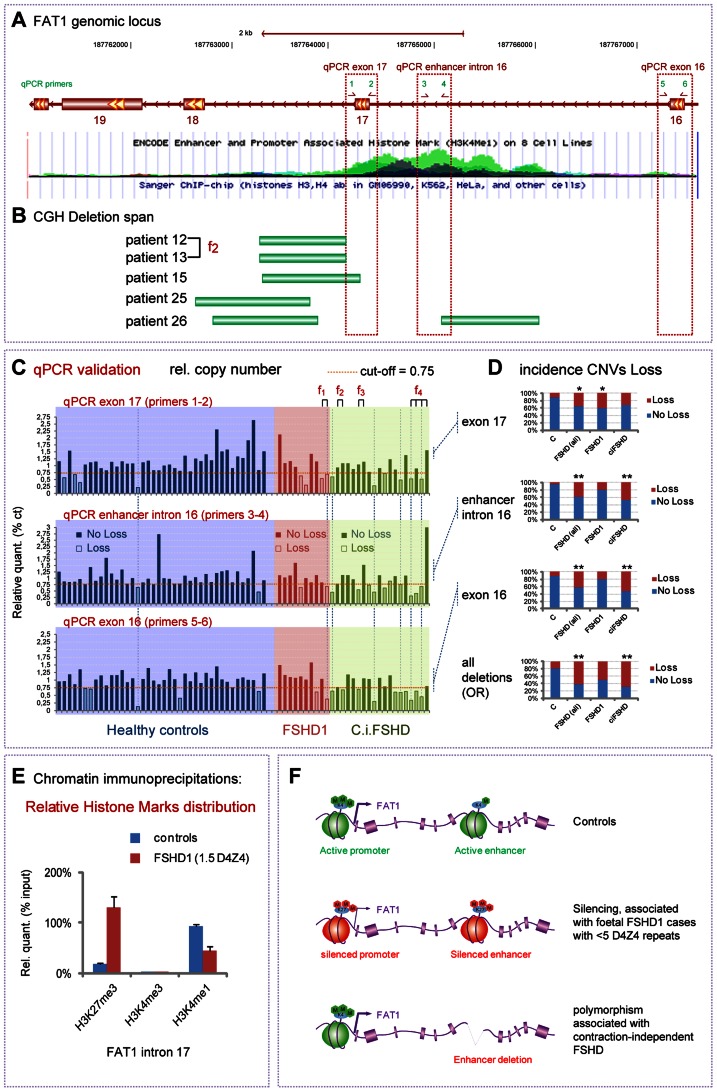
Identification of contraction-independent FSHD patients carrying deletions of an intronic *FAT1* enhancer. (**A**) View of the Human genomic *FAT1* locus focusing on an area including FAT1 exons 17-18-19. The lower image is a USCC browser based screen-copy image showing a track displaying ENCODE enhancer and promoter associated histone mark (H3K4me1) on 8 cell lines. (**B**) Positions of copy number variants identified in 5 FSHD patients by CGH and positioned on the genome by CGHweb analysis. Patients are identified with a specific number, and their characteristics are available in the Table S1. The deletion span varies from deletions restricted to intron 17 to deletions spanning over intron 17, exon 17 and intron 16, including a ENCODE-putative enhancer visible through genomic browsers. (**C**) Copy number validation of the deletion by qPCR. The three graphs show the relative amounts of PCR fragments obtained using primers couples 1–2 (exon 17), 2–3 (enhancer intron 16) and 4–5 (exon 16)), in a group of 40 healthy controls (blue area), a group of 10 FSHD1 patients (red area), and a group of 19 contraction-independent patients (c.i.FSHD). All data were normalized using an unrelated genomic fragment (Adora) as internal control, and one of the control DNAs (number 21) was used as the reference DNA (where all values are set to 1). A cut-off of 0.75 has been set. Individuals in which the relative value is lower than the cut-off are considered as having lowered copy numbers (indicated as loss). Information on each patient (regarding clinical and genetic diagnostic) are available in the Table S1. (**D**) The distribution of CNVs corresponding to loss CNV (seen as red) is shown in controls and in FSHD groups (all together, or FSHD1 and c.i.FSHD separately) for each of the three spots considered individually (top three graphs) or considered together (bottom plot, where loss represents the number of cases having a loss for at least one of the three spots). The cases where a significant link (as measured by X^2^ or Fischer tests) are indicated with one or two stars (* for p<0.05; ** for p<0.001; p-values indicated in the result section). (**E**) Analysis by Chromatin immunoprecipitation of the relative enrichment of the chromatin marks H3K27me3 (silenced chromatin), H3K4m3 (promoter active), and H3K4me1 (enhancer active), at the level of the intronic enhancer located between exons 17 and 18 of FAT1, in muscle extracts from two age matched controls and FSHD1 foetus F1. (**F**) Schematic summary of the finding, showing a conformation switch of the chromatin to repressed state in FSHD1 foetal muscle (in cases with severe expected outcome with <5 D4Z4 repeats), at the level of both the promoter and the intron 17/18 enhancer of *FAT1* exons (middle). (bottom) Deletions of part of all the enhancer at introns 16/17 are predicted to interfere with tissue-specific regulation of *FAT1* expression, and. represent (when carried on one allele) a polymorphism that segregates with FSHD (FSHD1 and c.i.FSHD).

*FAT1* deregulation is not the only gene expression change reported to be associated with the D4Z4 contraction causing FSHD1. As we also wished to determine to what extent the changes we found were relevant to the specific clinical phenotype, rather than a silent consequence of the D4Z4 contraction, we therefore extended our investigation to contraction-independent FSHD cases. Such patients have typical FSHD symptoms, but are not genetically associated to a pathogenic contraction of the D4Z4 array on chromosome 4. A large fraction of these contraction-independent FSHD cases is now known as FSHD2, in which hypomethylated D4Z4 repeats are combined with with a normal sized D4Z4 array on chromosome 4 permissive for DUX4 expression [22], [63]–[64]. Besides, other rare cases of contraction-independent FSHD cases remains unexplained, and represent interesting candidates to test whether alterations of the *FAT1* locus might be directly associated with FSHD. To identify such alterations of the *FAT1* locus, we performed an array-based comparative genomic hybridization screen (CHG [65]), a method used to uncover copy number variants. The custom-designed CGH array we employed covered the whole *FAT1* genomic region, including non-coding sequences. In our CGH survey of 29 FSHD cases, including 10 FSHD1 cases and 19 contraction-independent cases (5 of which at least not showing D4Z4 hypomethylation, see Table S1 for clinical and genetic characterization of patients), we detected 5 cases exhibiting loss of portions of the intron 17 (between exons 17 and 18), or intron 16 of the *FAT1* gene (Figure 10A,B, Figure S16). Besides the overlap with exon 17, we noticed that these deletions mapped near or within a hot spot of H3K4me1 methylation, a hallmark of cis-regulatory enhancers [61], spanning across intron 16 and part of intron 17 (Figure 10A, and Encode high throughput data, available on the UCSC browser [66]). According to the ENCODE ChIP seq data set [67], this element appears labeled as having strong enhancer activity in a human skeletal muscle myoblast line (HSMM) but not in 8 other non-muscle cell lines (Figure S16B). Examining the chromatin status at this locus by ChIP experiments, we consistently found that in control foetal muscle biopsies, intron 16 but also intron 17 were decorated by high levels of the enhancer signature H3K4me1 and negligible amounts of H3K4me3 (promoter signature) (Figure 10D, blue lanes, and data not shown), providing further in vivo support to the possibility that this sequence might indeed act as regulatory element in vivo.

To determine whether loss of functional portions of the putative enhancer were associated with FSHD, we analyzed copy number variants (CNVs) in a set of 40 healthy controls, 19 contraction-independent FSHD cases, and 10 FSHD1 cases. As the sensitivity of the CGH method might not allow detecting all cases with accurate precision, we applied a more precise qPCR method, and evaluated relative copy numbers by comparing 3 positions within and around the putative enhancer to a control spot on another chromosome (Figure 10A, C; 3 additional positions shown in Figure S16). Having set the threshold for considering a genome as carrying reduced copy numbers (loss) to 75% of the value in a healthy control used as reference genome, we found some healthy controls that exhibited reduced copy numbers of genomic regions at the core of the H3K4me1 hotspot in intron 16 (5% of controls) or in either surrounding exons (10% of control cases in both cases). This finding is consistent with a study, available through public datablases, that identified cases with loss of similar genomic segments at this locus in a group of 90 healthy individuals [68]. Thus, such deletions/copy number reductions are not sufficient on their own to cause FSHD symptoms, when occurring on only one allele of *FAT1*. However, in all three positions, the proportion of FSHD cases (all cases included) who exhibited loss was significantly higher than the proportion of healthy controls carrying reduced copy numbers at the same spot (Figure 10C,D; X^2^ test, p values<0.016; <0.00075; and <0.00041, for exon 17; enhancer; and exon 16, respectively). Cases with a deletion spanning the whole region were also significantly more frequent in the FSHD group than among controls. When considering only contraction-independent FSHD cases, as much as 47% carried the CNV including the putative enhancer, as compared to 5% of controls, and up to 68% carried a CNV encompassing at least one of the three considered positions, as opposed to 20% of the controls (Figure 10C,D, Fischer test, p<0.0004 and p<0.0001 for enhancer and exon 16, respectively). Conversely, when considering the distribution of cases with increased copy numbers (gain, above a threshold of 1.25× over the average control value) we found that there were significantly less FSHD cases with gain-CNVs than among the control group (X^2^ test, p<0.017 and p<0.014 when considering all FSHD cases or contraction-independenty cases only, respectively). Finally, we also analyzed the methylation status at D4Z4 repeats on chromosome 4 on a subset of our group of contraction-independent FSHD patients (5 out of 19), and found no indication of hypomethylation (at the CpoI site, Table S1) on the proximal D4Z4 unit [64]. This does not exclude that others patients in our c.i-FSHD group would be diagnosed as FSHD2, but indicates that FSHD can occur in non-contracted patients independently of the hypomethylation, known FSHD2 hallmark [22], [64]. Together, these results indicate that partial or complete deletions of *FAT1* intron 16/17 putative enhancer represent a polymorphism not sufficient to cause FSHD by itself when present on one allele only of chromosome 4, but which segregates with FSHD. Therefore, this CNV can be combined with pathogenic or sub-pathogenic contexts, and may act as a novel disease modifier in FSHD.

## Discussion

FAT-like cadherins play various roles in tissue morphogenesis, by modulating cell polarity, adhesion and tissue growth. Here we show that during development, *FAT1* controls the shape of subsets of muscles in the facial and scapulohumeral regions, and does so by modulating the polarity of collective myoblast migration, a function in accordance with the emerging link between planar cell polarity and collective directional migration events [29]–[30], [69]. These muscle shape abnormalities are predictive of early onset muscle wasting, as observed in *Fat1*-deficient mice that bypassed neonatal lethality. Using *Pax3-cre* for conditional ablation of *Fat1* functions in premigratory myoblasts, we show that a cell autonomous requirement for *Fat1* function in the migrating myoblasts accounts for a significant component of this role in shaping muscles. Taken together, the location of the human *FAT1* gene next to the critical FSHD locus at 4q35, the similarity between the *Fat1*-dependent muscles and those affected in FSHD, and the appearance in *Fat1* mutants of non-muscle features of FSHD, suggest a possible role of *FAT1* in the pathophysiology of this disease. In our human studies, we found two ways by which altered *FAT1* regulation underlies a link with FSHD: 1- we observed muscle-specific lowering in foetal FSHD1 biopsies; 2- we identified a polymorphism deleting a putative cis-regulatory enhancer in the *FAT1* locus, which significantly segregated with FSHD. Together, these results strongly support the idea that tissue-specific de-regulation of *FAT1* expression/function might play a critical role in FSHD pathophysiology.

### *Fat1* is required in migrating myoblasts to shape selective muscles in the face and shoulder

The altered myoblast migration polarity caused by loss of *Fat1* functions leads to selective developmental dysgenesis of scapulo-humeral and subsets of subcutaneous muscles of the face. Understanding how *Fat1* controls muscle shape required first determining which part of its expression domain accounts for this function. In addition to the muscles, *Fat1* is expressed in several of the cell types that interact with migrating muscle cells. The highest expression was seen in non-muscle cells, such as the subcutaneous layer towards which CM myoblasts migrate (Figure 1). This muscle-skin interface is analogous to the bone-muscle interfaces (tendons, joints) of skeletal muscles, where *Fat1* also accumulates at later stages (Figure 7A). Here, however, we show that ablating the floxed transmembrane domain of FAT1 with a *Pax3-cre* knock-in line leads to efficient excision in premigratory muscles of the limb but not the face, and reproduces at least partially the migration phenotype observed in constitutive *Fat1* knockouts in the scapulohumeral region. *Pax3-cre* excision does not occur in motor neurons, hence ablation in this cell type does not contribute to the phenotype observed in *Fat1^Fln/Fln^*;*Pax3^ cre/+^* embryos. No significant muscle shape defects were caused by *Pax3-cre* -mediated *Fat1* ablation in subcutaneous muscles of the face. This is not surprising, as muscles in the face do not derive from *Pax3*-expressing precursors but were previously shown to derive from a subset of *islet1*-expressing pharyngeal mesoderm cells [46], [70]. In addition to trunk migrating myoblasts, *Pax3-cre*-mediated excision occurs in dorsal neural tube and neural crest. Although *Fat1* expression is detected in Schwann cells (neural crest-derived) along the nerves at P0, we did not detect such an expression at the stage of muscle migration (E12.5, see Figure S11C), making it unlikely to for *Fat1* to control migration polarity by acting in neural crest derivatives. Furthermore, as *Pax3-cre*-derived neural crest amply colonizes the developing face, the lack of enhanced muscle phenotype in the face of *Fat1^Fln/Fln^*;*Pax3^ cre/+^* embryos disqualifies the neural crest component of *Fat1* expression from playing a major contribution in muscle shaping, and strongly suggests that *Fat1* is required cell-autonomously in migrating myoblasts to control the polarity of their migration. As however, the muscle phenotype of *Fat1^Fln/Fln^*;*Pax3^ cre/+^* embryos is significantly weaker than the phenotype of constitutive mutants, it leaves the possibility that other component of *Fat1* expression domain may also contribute to its function in muscle patterning.

The rationale for why such a selective group of muscles is affected by *Fat1* loss of function is still unclear. This group of muscle includes subsets of migratory muscles of the face and shoulder area. In the face, defects are restricted to branchiomeric muscles derived from the second brachial arch (subcutaneous muscles of the skin, Figure 3), while first branchial arch derived muscles (masseters and temporalis), as well as extraocular muscles, are unaffected (Figure 5 and data not shown) [70]–[72]. The scapulohumeral region can be divided in two components: 1) the CM, as well as humeral muscles (triceps, deltoid, or muscles which pattern is affected by the supernumerary muscle) derive from somitic *Pax3*-driven hypaxial migratory precursors (Figure S10); 2) In contrast, some of the shoulder muscles such as the acromiotrapezius and spinotrapezius, or the rhomboids, belong to the cucullaris group and were previously shown to derive from non-somitic, occipital lateral plate mesoderm [46], [72]–[73]. Such specificity is in apparent contrast with the broader expression domain of *Fat1* in muscles as observed at E12.5 and later (Figure 1, 7, and S1), although clear differences in expression levels between muscles can be distinguished (Figure 7A). Given that distinct regulatory programs govern the development of these muscle groups [2], [74], the selective impact of *Fat1* on muscle shapes could be determined by its interaction with some of the selective myogenic regulators.

### Does altered muscle shape predispose to muscle wasting?

Advanced symptomatic stages in *Fat1*-deficient mice are likely systemic consequences of such non-muscle phenotypes. Nevertheless, the muscle wasting and dystrophic features measured at presymptomatic stages were detectable selectively in those muscles that exhibited myofiber orientation defects, even in cases with no other detectable phenotypes. Despite the important variability in postnatal phenotype strengths observed with the *Fat1^LacZ^* allele, myofibre orientation defects and dystrophic features in the CM and shoulder muscles (Rhomboids, Trapeze) were observed in all mutant cases examined, not only of embryos, but also at adult stages, even in cases of *Fat1^LacZ/LacZ^* mice surviving to old ages with no other detectable phenotype. This specificity argues against the idea that restricted topography of muscle defects would be a consequence of renal problems or of other non-muscular defects. Furthermore, the observed match between the topography of the developmental phenotype and the specific map of muscles that undergo wasting at presymptomatic stages in adult *Fat1^LacZ/LacZ^* mice supports the idea that the selective muscle degeneration might occur as a consequence of the altered muscle shape. Future experiments will be necessary to determine whether the limited defects observed in *Pax3-cre/Fat1* embryos are sufficient to predispose muscles to early onset degeneration, and whether additional triggers might be required for degeneration to occur in adult life. Among phenotypes observed in adult *Fat1*-deficient muscles, it will also be interesting to distinguish secondary consequence of the altered muscle shapes, from phenotypes reflecting additional, independent functions of *Fat1*, whether exerted in muscles too or in other cell types.

### Tissue-specific de-regulation of *FAT1* as a potential mechanism in FSHD pathogenesis

The spatial distribution of muscles mis-shaped as a result of *Fat1* loss of function as seen at E14.5/E15.5 (Figure 3) appears to overlap very closely with, and thus to predict, the map of muscles affected at early stages in FSHD. Furthermore, the observation of non-muscle phenotypes such as defects in retinal vascularisation or inner ear patterning also bears some similarities with symptoms observed in FSHD patients. Despite this strong concordance between the phenotype of *Fat1*-deficient mice and FSHD symptoms, the selectivity of the shared phenotypes raises a paradox. *Fat1* expression during development is not restricted to FSHD-relevant tissues, and constitutive deletion of *Fat1* leads to pronounced renal defects and neonatal lethality. Even the *Fat1* hypomorphic phenotypes presented above cannot be considered as an exact phenocopy of FSHD. Overall this mouse model is also more severe than FSHD, and 50% of the mice die within 3 months, likely of milder versions of the kidney phenotype (such as polycystic kidney). In contrast, FSHD is not known as a lethal disease, and has no reported association with kidney problems. Absence of renal dysfunction in FSHD is a strong indication that FSHD cannot simply be considered a “*FAT1* knockout”. Thus, cases of patients with severe *FAT1* loss of functions and kidney failure might be fatal before onset of muscle dystrophy and might thus fail to be classified as FSHD. In support of this hypothesis, a rare case of a 5-year-old girl carrying a duplication of the *D4Z4 array* and showing vascular retinopathy and sensorineural deafness was also reported to have focal glomerulosclerosis of the kidney [75]. Instead, lack of association between FSHD and renal dysfunction indicates that any FSHD mechanism involving *FAT1* alterations must necessarily preserve FAT1 expression/function in kidney (at least). Our results with mice suggest that such selective alterations of *FAT1* function/expression may matter during development, in muscle precursors, at a stage when their migration occurs, for which FSHD human material was not available so far - and can ethically not be sought. *FAT1* levels may not be changed to an equal extent in all tissues and times, consistent with our observation that *FAT1* levels were reduced in disease-relevant muscles but not in brain, and at foetal but not adult stages. Thus, an engineered mouse model in which *Fat1* functions are specifically ablated in muscles and preserved in the renal system, even though lacking effects of other DUX4 target genes, may represent a more suitable tool to study consequences of the muscle abnormalities in adult, and a better model reflecting the tissue-specific *FAT1* depletion that we propose might be occurring in FSHD.

### Loss of a putative *FAT1* enhancer as a novel disease modifier in FSHD

The finding that human cases of contraction-independent FSHD, with such a characteristic and restricted set of clinical symptoms, segregate with the deletion of a putative regulatory genomic element in the *FAT1* locus instead of the traditional *D4Z4* contraction, strongly supports the idea that altered *FAT1* regulation plays a key role in the pathology. The putative cis-regulatory enhancer reported in this study, which deletion segregates with FSHD in contraction-independent cases is likely to carry tissue-specificity information driving *FAT1* expression in FSHD-relevant cell types, and future experiments are required to demonstrate such activity. The finding that healthy controls can exhibit heterozygous loss of this fragment of the *FAT1* locus, containing two exons and an enhancer, is consistent with the observation that heterozygous loss of *Fat1* functions in mice does not have major consequence of life span, health, and muscle integrity. However, we did observe a significant degree of haploinsufficiency in *Fat1^ΔTM/+^* embryos, evidenced by the presence of subtle muscle shape defects (Figure 6B,C, see indicated p values), the most frequent position being between the acromyotrapezius and spinotrapezius muscles (as in Figure 3B). Such phenotypes were also consistently detected in *Fat1^Fln/Fln^* embryos (Figure 6 and Figure S10D), in which expression levels were similar to those measured in *Fat1^ΔTM/+^* embryos (Figure S10B, C), suggesting that muscles in the shoulder area are highly sensitive to *Fat1* dosage. While copy number variants outside of the putative enhancer might occur without causing any regulation change, we reasoned that the further such deletions would extend into the ENCODE predicted enhancer, the more functional transcription factor binding sites they may remove, hence increasingly interfering with *FAT1* regulation on the deleted allele, thereby sensitizing the locus to additional contexts that may additionally impact on *FAT1* expression.

Interestingly, two of the FSHD1 cases presented here were monozygotic twins, both carrying a contracted 4q35 allele with 3 D4Z4 units, one of the twins being asymptomatic while the other twin had been diagnosed with a classical FSHD. We found that the twin with FSHD symptoms displayed reduced copy numbers throughout the length of the studied area, encompassing both exons 16 and 17 and the intron 16 putative enhancer, while the asymptomatic twin exhibited reduced copy numbers only at the distal-most region towards exon 16, this difference possibly representing a de novo somatic mutation (Figure 10 and Table S1). Although this correlation does not constitute a demonstration of causality, it provides support to the hypothesis that this lowered copy numbers (heterozygous) of *FAT1* exons 17/16 and of portions of the putative *FAT1* enhancer portions have the potential to worsen FSHD symptoms when combined to a pathogenic context. However, obtaining a formal demonstration of this hypothesis will require studying phenotypes/genotype correlations on a large cohort of patients, and knowing in each case if the FSHD-causing genetic context is FSHD1, FSHD2, or other un-identified contraction-independent contexts. Overall, deregulation of the *FAT1* gene is associated with FSHD, either as a consequence of DUX4 overexpression, and/or epigenetically encoded in FSHD1 and FSHD2, or through the deletion of a putative enhancer that segregates with contraction-independent FSHD patients.

### An additional function for *FAT1* in muscle differentiation and/or physiology?

Among possible products of the *Fat1*-gene, our results in mice indicate that the control of migration polarity and muscle shape requires a *Fat1* RNA containing a transmembrane domain encoded by the floxed exons and deleted in the *Fat1^ΔTM^* allele. In contrast, other functions can be executed by incomplete *Fat1* isoforms. Residual RNAs containing 3′ *Fat1* exons can rescue (to an extent correlating with RNA levels) kidney defects and their consequences, but not muscle dysgenesis. Interestingly, however, both mouse models retain the capacity to produce FAT1 protein isoforms containing an intracellular domain, albeit at reduced levels quantified by qPCRs (Figure S10B,C), ruling out a major contribution of these isoforms to the muscle shape phenotypes observed in both mouse models. In muscle fibres, FAT1 is a novel component of t-tubules. Does *Fat1* expression in differentiating and mature muscle reflect additional functions in muscle biology? The presence of FAT1 protein in close association with the contractile apparatus, as soon as differentiation starts, may reflect a role in sarcomere assembly. These FAT1-enriched stripes are maintained in mature muscle fibres, tightly juxtaposed with the t-tubule system (Figure 7B). This may indicate a further involvement in excitation-contraction coupling, an essential process required throughout adult life for muscle function and maintenance. However, this striped pattern is established as early as the contractile apparatus assembles (Figure 7C), before the alignment and docking of T-tubules to the contractile apparatus takes place, the latter phenomenon occuring postnatally in mice [76]. This indicates that in muscle, FAT1 isoforms are not inserted in the t-tubule compartment itself, but may be located at an interface juxtaposing t-tubules and the contractile units, possibly reflecting a new function for *Fat1* for example during assembly of the t-tubule network. As myoblast migration precedes differentiation and sarcomere assembly, the accumulation of these FAT1 protein isoforms in the contractile apparatus occurs too late to be accountable of the function in migration polarity.

FAT-like proteins were previously reported to be subject to various cleavage events by Furin convertase or by α- or γ-secretases [50]–[52]. Furthermore, alternative splicing events in the cytoplasmic exons were reported to influence subcellular targeting of FAT1 proteins [48]. Our work in mice unexpectedly indicated that in addition to producing a large transmembrane protein and its cleavage products, the *Fat1* gene also produces small molecular weight protein products which appear not to contain a transmembrane domain, and synthesis of which is largely preserved in both *Fat1*-deficient mouse models, although at reduced levels. Bioinformatic scans and existing ESTs reported on all genomic browsers are indeed consistent with the possibility that short isoforms may result from transcript initiation at alternative downstream promoters, and may code for protein products devoid of leader peptide and transmembrane domain and potentially produced in the cytosol (lacking a leader sequence). Thus, understanding the roles played by the isoforms of FAT1 produced in muscles will require first characterizing the exact exon and domain composition of the *Fat1* RNA and protein isoforms produced in muscle (wild type and *Fat1^ΔTM/ΔTM^*), and second designing novel strategies to ablate them independently of the transmembrane domain containing isoforms.

Interestingly, residual expression of such muscle-specific isoforms is genetic background dependent and its levels in *Fat1^LacZ/LacZ^* mice inversely correlated with phenotype severity. Furthermore, reduced expression levels and abnormal sub-cellular localization were observed in muscle of human foetal cases with expected severe and early onset FSHD1 (as predicted by the degree of D4Z4 contraction and family history), while no significant changes in RNA levels were detected in adult FSHD1 muscles compared to controls. These observations are consistent with the idea that deregulated *FAT1* expression in differentiated muscle may be predictive of early (infantile) onset and severe dystrophy. These data suggest that the causes of the early phase, common to all FSHD patients and restricted to muscles of the face and shoulder, might be uncoupled from the causes of later phases of the disease - which spreads to other muscles, a condition that occurs in a subset of FSHD patients with childhood onset, the latter ending up wheel-chair bound [6].

### Link with the known mechanisms in FSHD1

Recent studies have brought to light several possible molecular pathways by which the *D4Z4* contraction on a 4qA allele may exert its pathological effect in FSHD1. Among those, stabilization of *DUX4-fl* mRNAs by polyA-creating polymorphisms was shown to enable expression of a toxic form of DUX4, the latter causing muscle dystrophy through altered regulation of numerous target genes, including *Pitx1*, *p53*, and other germline-specific genes or myogenic regulators [17]–[21], [23]–[24]. Another mechanism involves production by the contracted region of DBE-T, a chromatin-associated long-non-coding RNA that causes de-repression of several 4q35 genes [77], including *FRG1*, whose overexpression was previously proposed to contribute to causing muscle degeneration too [11]–[12]. Other mechanisms also influencing 4q35 gene expression include a telomeric position effect, according to which propagation across 4q35 of changes in methylation or chromatin conformation might be due to the loss of the CTCF barrier function of the *D4Z4* array [8], [13], [78]. The relative contribution of DUX4-mediated gene regulation and of mechanisms leading to altered 4q35 gene expression is controversial [9], [17] and may reflect an underestimated diversity in the clinical expression of FSHD1 [79]–[80]. Understanding which of these mechanisms, or what combination, contributes to modifying tissue-specific distribution of *FAT1* will require developing cellular or animal models adequately reproducing FSHD mechanisms and mimicking in vitro key steps of muscle shape development. This will also allow defining whether there are differences in the sensitivity to a contracted allele between developmental stages and adult muscle, but also between FAT1 isoforms. DUX4 can repress *FAT1* expression in human myoblasts ([18] and Figure S15D). Such regulatory influence could involve some DUX4 target genes such as p53 [37]–[38], or myogenic transcription factors. Our data suggest that irrespective of whether *FAT1* is regulated by DUX4, by *DBE-T*, or by anyone of their respective downstream or upstream targets, this regulation must occur primarily during development, in the cell type in which *FAT1* is required to control migration polarity. This model does not exclude the possibility that the pathogenic 4q35 allele may further contribute to directly triggering muscular dystrophy in adult muscle, through additional mechanisms independent of *FAT1* de-regulation.

### Can deregulated *FAT1* in FSHD lead to altered PCP/Frizzled signaling?

A number of clinical features of FSHD, including non-muscular symptoms such as hearing loss and retinal vasculopathy [81]–[82], carry the signature of defects in the Wnt/PCP pathway [26], a cascade of tissue polarity regulating genes, involving non-canonical Wnt/Frizzled signalling (core PCP genes) and modulated by the protocadherins FAT and Dachsous [25], [27]. Sensory hair cell polarity in the cochlea is the best mammalian PCP paradigm, and deafness has become a traditional hallmark of altered PCP signalling [26], [57]–[58]. Even through the anatomical nature of auditory abnormalities in FSHD is not known, it will be relevant to explore whether it carries further characteristics in common with altered PCP. Furthermore, vascular abnormalities in the retina, also known as Coats disease, are phenotypically similar to familial exudative vitroretinopathy (FEVR), recently linked to mutations in the Wnt receptor Frizzled4 (FZD4) and its ligand Norrin [83]–[85]. Moreover, the Wnt/PCP pathway is also known to play key roles in muscle biology. PCP-activating Wnts, such as Wnt11 or Wnt7a act as instructive signals for myofibre orientation during muscle morphogenesis [86], for muscle satellite cell expansion through symmetric division [87], and for neuromuscular synapse development [88]. Thus, altered regulation of *FAT1* may in turn de-regulate the function or expression of its genetic partners, such as other components of the planar cell polarity cascade but also of the Hippo pathway. Mutations in other components of these genetic cascades may also play a causal role in a subset of the FSHD patients lacking the *D4Z4* contraction. Overall, by linking FSHD to *FAT1*, our work opens new avenues for the exploration and treatment of this and other neuromuscular disorders.

## Methods

### Ethics statement

Animals were maintained and sacrificed in accordance with institutional guidelines. Adult mice were either sacrificed for experiments through anaesthesia, or euthanized by cervical dislocation. Efforts were made to minimize the number of adult *Fat1*-deficient mutant mice examined after more than 25% weight loss.

Human DNAs were obtained from FSHD and control cases at La Timone Hospital (Marseille, France). The protocol for their collection was approved by the Université de la Méditerranée (Marseille, France) Committee on Human Research and an agreement of informed consent authorizing scientific experiments was signed by each individual patients. Human Tissues samples were obtained from abortus cases at La Timone Hospital (Marseille, France) and at AP-HP (Assistance Publique-Hopitaux de Paris, France). The protocol for their collection was approved by the Université de la Méditerranée (Marseille, France) Committee on Human Research and an agreement authorizing scientific experiments was signed by the parents. Termination of pregnancy (performed at the stages corresponding to individual cases) was decided after late prenatal diagnosis.

### Mouse lines

#### Characterization and genotyping of the *Fat1^LacZ^* allele

*Fat-^LacZ^* mice, previously generated using the genetrap ES line KST249 (see detailed characterization below), were obtained from Marc Tessier-Lavigne. Initial characterization of the transgene insertion site on *Fat1* transcript was performed by 5′RACE PCR (as documented on the international genetrap consortium databases (http://www.genetrap.org/cgi-bin/annotation.py?cellline=KST249) indicated insertion downstream of the first exon. Genome walking experiments (LAM-PCR, GATC Biotech; plasmid rescue, restriction analysis by Southern blot) consistently indicated that multiple copies of the transgene were inserted in tandem, thus preventing so far identification of genomic sequences flanking the transgene. Genotyping was performed on genomic DNA using the following PCR to detect the transgene (primer sets OF47: 5′ GGA ACT TCT CAG ATC TGC GGG CTGC 3′; and OF48: 5′-TCT CAT CTT GGG TGA GGT GGG TCCC-3′; or OF49: 5′-GGA ACT TCT GGA TCT GCG ATC TGCG-3′ and OF57: 5′ CCC CAA ACA CTG CCA ACT ATG-3′). To recognise heterozygotes (one mutant allele) from homozygotes (two mutant alleles) at postnatal and adult stages, we performed dot blot hybridization experiments (using a beta-geo probe made with OF47-OF48 PCR product, dig-labelled by random priming) and discriminated difference in staining intensity, or qPCR analysis, using OF47 and OF48 primers for the transgene, and the following primers as reference (met primers wt: FM20: 5′ AAG CTT CTG GTT CTG ATG CTC TGT CAG -3′; Met-610: 5′- AGG ATT GAT CAT TGG TGC GGT C – 3′). At embryonic stages, we also performed X-gal staining on yolk sacs (or any dissected fragment of embryo), the intensity of staining being a reliable indicator of the genotype until E16.5.

To follow progression through adult phenotype, each mouse's weight was measured weekly, and the weight at a given stage is compared to it maximal measured weight. Mice with less than 10% weight loss are considered presymptomatic (with respect to systemic consequences of phenotypes such as kidney filtration defects).

#### Transgenic mouse lines

*Gdnf-lacZ* mice were used with permission of Genentech, and genotyped as previously described [40]. Mlc3f-2E transgenic mice were kindly provided by Robert Kelly, and genotyped as previously described [43]. *Pax3-cre* knock-in (Pax3^tm1(cre)^Joe line [89]) mice were used with permission of Jonathan Epstein and genotyped with the following generic CRE-specific primers: MSP4: 5′-ATC CGA AAA GAA AAC GTT GA-3′; MSP5 5′-ATC CAG GTT ACG GAT ATAG T-3′. Rosa26-YFP mice (Gt(ROSA)26Sor^tm1(EYFP)^Cos line, [90]) were kindly provided by Teddy Fauquier and obtained from the Jackson laboratory (mouse strain 006148), and were genotyped following Jaxmice instructions.

#### Generation of *Fat1* conditional and constitutive mutants

Mice carrying a conditional *Fat1^ΔTM^* allele were constructed in the research facility of iTL (ingenious Targeting laboratories, genetargeting.com). Construction of mice with the conditional *Fat1^ΔTM^* allele began by isolation of the 129 SvEv BAC clone RP22: 41E14, containing the murine sequence of the *Fat1* locus, including the exons 24 and 25, which we aimed to flox, exon 25 containing the transmembrane domain. An 11 kb region used to construct the targeting vector was first subcloned from the BAC using a homologous recombination-based technique. The region was designed such that the short homology arm (SA) extends about 2.3 kb to the 5′-end of the LoxP/FRT-flanked Neo cassette. The long homology arm (LA) extends 6.22 kb to the 3′-end of the single Lox P site. The single Lox P site is inserted upstream of exon 24 in intron 23–24, and the LoxP/FRT-flanked Neo cassette is inserted downstream of exon 25 in intron 25–26. The target region is ∼2.6 kb containing exons 24–25. The BAC was sub cloned into a ∼2.4 kb pSP72 (Promega) backbone vector containing an ampicillin selection cassette for retransformation of the construct prior to electroporation. A pGK-gb2 LoxP/FRT-flanked Neomycin cassette was inserted into the gene as described in Figure S4. The targeting construct can be linearized using NotI prior to electroporation into ES cells. Ten micrograms of the targeting vector was linearized by NotI, and transfected by electroporation of iTL1 129/SvEv embryonic stem cells. After selection with G418 antibiotic, surviving clones were expanded for PCR analysis to identify recombinant ES clones. Screening primers A1 and A2 were designed downstream of the short homology arm (SA) outside the 3′ region used to generate the targeting construct. PCR reactions using A1 or A2 with the F3 primer (located within the Neo cassette) amplify 2.42 or 2.51 kb fragments, respectively. The control PCR reaction was performed using the internal targeting vector primers AT1 and AT2, which are located at the 3′ and 5′ ends, respectively, of the SA. This amplifies a product 2.05 kb in size. Primers for PCR Screening : A1: 5′- AAG CTT CCT GCT GTC ACT AAG G -3′; A2: 5′- ACG TGC ATG TTA ACT GGG TAC AC-3′; AT1: 5′- AGG TTC TGA ACA GGG AAT GAA ACG – 3′; AT2: 5′- TCT GTT GAG CAT ATG TGC AGA TC – 3′; OUT1: 5′- GGC TGC TAC GTC TCA GGG C – 3′; F3: 5′- GCA TAA GCT TGG ATC CGT TCT TCG GAC – 3′. Individual clones from positive pooled samples were screened using A1 and F3 primers. Positive recombinant clones were identified by a 2.42 kb PCR fragment. A PCR was performed on SA positive clones to detect presence of the third LoxP site using the LOX1 and SDL2 primers. This reaction amplifies a wild type product 390 bp in size, and a product from the targeted locus of 452 bp in size. 3 clones were reconfirmed for SA integration and third LoxP site retention using the same methods as described above. All reconfirmed clones were sequenced to verify SA integration and retention of the third LoxP site. Secondary confirmation of positive clones identified by PCR was performed by Southern Blotting analysis. DNA was digested with NcoI, and electrophoretically separated on a 0.8% agarose gel. After transfer to a nylon membrane, the digested DNA was hybridized with a probe targeted against the 3′ external region. PB1/2 Probe Primers: PB1 5′-TCA GCT CAC CCA GCT AAT GC – 3′; PB2 5′-TCA ACG ACA GCG TTG ACA AGG – 3′. Positive clones identified by PCR were further confirmed by Southern Blotting analysis with an internal probe. DNA was digested with NcoI, and electrophoretically separated on a 0.8% agarose gel. After transfer to a nylon membrane, the digested DNA was hybridized with a probe targeted against the 3′ internal region. Two clones were confirmed as correctly targeted and used for injection in blastocysts. Targeted iTL1 (129/SvEv) embryonic stem cells were microinjected into C57BL/6 blastocysts. Resulting chimeras with a high percentage agouti coat color were mated to wild-type C57BL/6 mice to generate F1 heterozygous offspring. Tail DNA was analyzed as described in Figure S4A from pups with agouti coat color. Heterozygous F1 offspring carrying the targeted allele were further reconfirmed using the PCR conditions used for ES screening (A1 and F3 primers). Germline transmission of the targeted allele was obtained for one recombined ES clone.

Mice carrying the targeted locus (refered to as *Fat1^Fln^* allele, as it contains the floxed exon and the neo cassette) were amplified, and kept as either heterozygotes or homozygotes, as *Fat1^Fln/Fln^* males and females are viable and largely fertile. Mice carrying the *Fat1^Fln^* allele were crossed with mice carrying the ubiquitous cre (Deleter-cre line, official name Tg(CMV-cre)1Cgn [91]) to produce offspring with a recombined allele (Refered to as *Fat1^ΔTM^*). After elimination of the *Deleter-cre* transgene, a line with the recombined allele was maintained separately from the line carrying the original *Fat1^Fln^* allele. *Fat1^ΔTM/+^* (also carrying the *MLC3F-2E* transgene) were bred to produce *Fat1^ΔTM/ΔTM^* embryos or mice. Genotyping primers (numbered according to Figure S3): Primer 1 (OF165) : 5′ - GTA GGG ACG TTC TGT GAG GTG AGC G – 3′ ; primer 2 (OF166): 5′ – CTG TGG AAA GGG CGC AGC AGA AAC G- 3′; primer 3 (ON17) : 5′ - AAC TCG CCC TCA GAC AGC GAC TCC - 3′; Primer 4 (ON16); 5′ – GGG ATC CAG ATC TAC CAC TTT AGC TG – 3′; Primer 5 (Uni): 5′ - AGC GCA TCG CCT TCT ATC GCC TTC; Primer 6 (LAN1): 5′ – CCA GAG GCC ACT TGT GTA GC – 3′.; Primer 7 (ON30) : 5′ – GTC CGA AGA ACG GAT CCA AGC TTA TGC – 3′. A combination of primers OF165 and OF166 amplifies a band of 458 bp on the wild-type DNA, a band of 520 bp corresponding to the *Fat1^Fln^* allele, and no product from the *Fat1^ΔTM^* allele. The *Fat1^ΔTM^* allele is recognised by a 305 bp product obtained with a combination of OF165 and ON30 primers. The neo cassette can be removed using FRT/Flp recombination (performed with *ActFlpe* mice, official name Tg(ACTFLPe)9205Dym [92]), generating a new conditional allele referred to as *Fat1^Flox^*. The *Fat1^Fln^* and *Fat1^Flox^* alleles can be recognised from each other using specific PCR products (For *Fat1^Fln^*, the combination of ON17 and Uni primer yields a 406 bp product; for *Fat1^Flox^*, the combination of ON17 and ON30 yields a 345 bp PCR product). In the current study, all the conditional experiments were done using the *Fat1^Fln^* allele. To perform the conditional ablation, of *Fat1* in *Pax3-cre*-derived tissues, we mated *Fat1^Fln/Fln^* mice with *Pax3^cre/+^* mice. Because of our observation of female germline activity of the *Pax3-cre* line, we exclusively selected *Fat1^Fln/+^; Pax3^cre/+^* males to perform crosses with *Fat1^Fln/+^* or *Fat1^Fln/Fln^* females (also carrying the *MLC3F-2E* transgene) to produce conditional embryos.

### Human tissue collection

Human Tissues samples were obtained from abortus cases (see ethics statement) after termination of pregnancy, decided after late prenatal diagnosis (of FSHD1 or of non muscular medical symptoms for control cases). The cases used, and their respective stages are described in Figure S8A. Four cases of foetuses diagnosed with FSHD were used (Figure S8A) referred to as F1, F2, F3 and F4, respectively. Family history included in the F1 case early-onset and severe FSHD phenotypes in a sibship carrying the same haplotype (family tree shown in Figure S8D). In the she second (F2) and third (F3 and F4, twin foetuses) cases one parent had FSHD. FSHD diagnosis was characterized by standard procedures involving southern blotting using a combination of restriction enzymes and probes, to characterize contraction status, 10 versus 4 chromosome, and haplotype. The p13E-11 probe was used on genomic DNA digested with EcoRI alone or with EcoRI and BlnI, hence determining D4Z4 array length and distinguishing and 4q contractions from 10q contractions [93]. Molecular combing is then performed with a combination of probes (including those for D4Z4, the p13E-11, qA and qB-specific probes, and 10q versus 4q specific) allowing to distinguish simultaneously 10q from 4q as well as qA from qB haplotypes and the degree of contraction [94] (see also simplified probe set in Figure S14B–E). Control biopsies (Figure S14A) were also obtained from abortus cases, for which termination of pregnancy was performed on the basis of medical diagnosis different from FSHD or other muscle related diseases. Detailed information on clinical and genetic diagnostic for the patients used for CGH and qPCR studies is provided in the Table S1.

### X-gal staining, immunohistochemistry, antibodies

X-gal staining was performed using classical procedures on embryos or postnatal tissues previously fixed in paraformaldehyde (PFA) 4% (time depending on strength of lacZ expression), rinsed in PBS, and incubated in X-gal in combination with potassium ferri- and ferro-cyanide (FeCN). Staining was terminated by rinsing in PBS, and post-fixing in PFA4%. Embryos were transferred in 100% glycerol for imaging and counting dispersed myoblasts.

For adult murine tissues, anaesthetized mice were perfused with PFA 4% in phosphate buffer saline (PBS) prior to dissection. Shoulder belt muscles were carefully dissected under a stereomicroscope, rinsed in PBS, shortly incubated with fluorescent alpha-Bungarotoxin to visualise neuromuscular junctions. When necessary, observation under fluorescence was used to visualise and sub-dissect zones enriched in neuromuscular junctions. Samples were cryoprotected in 25% sucrose (in PBS), embedded in a mix with 7.5% gelatine and 15% sucrose in PBS, and frozen for cryostat sections.

Immunofluorescence was performed using primary antibodies to neurofilament (NF-M, Ab1789, Chemicon), tau (AbCAM), laminin (Sigma), alpha-actinin (Clone EA-53, Sigma), Ryanodine Receptor RyR1 (MA3-925, Thermo scientific), Dyhydropyridine Receptor alpha 1S (MA3-920, Thermo scientific), rabbit anti-GFP (invitrogen). Antibodies against FAT1 were the following: two rabbit polyclonal antibodies raised against human FAT1, HPA001869, HPA023882 from SIGMA (epitopes described in the human protein Atlas (http://www.proteinatlas.org) recognised two regions of the extracellular domain of FAT1 indicated FAT1-1869 and FAT1-23882, respectively, in Figure 2A, B. Two antibodies against the intracellular domain of mouse FAT1 were used: Fat1-ICD from ref [35], and an additional anti-Fat1 rabbit antisera (Rb1465) we raised against a GST-fusion protein encompassing the intracellular domain of mouse FAT1 (see complete procedure below). Secondary antibodies used were Cy3- or Cy5-conjugated (Jackson Immunoresearch) or conjugated with Alexa-488 or Alexa-555 (Invitrogen). NMJs were visualised with Alexa-488 conjugated alpha-Bungarotoxin (1/2000), and F-actin with alexa-594 or Alexa-647 conjugated-Phalloidin (Invitrogen). Retinal vasculature was visualised with Alexa-488-conjugated GS-IB4 (Invitrogen), as described [83], including CaCl2 1 mM and MgCl2 1 mM in all incubating solutions. Image acquisition was performed with a Zeiss Axioplan equipped with Apotome.

### Ultrastructure studies

For electron microscopy analysis, muscles were dissected from mice previously perfused in 4% PFA, and post-fixed in 2% PFA, 2.5% glutaraldehyde, 50 mM CaCl2 in 0.1 M cacodylate buffer (pH 7.4). Muscles were additionally postfixed with 1% OsO4, 0.1M cacodylate buffer (pH 7.4) for 2 h at 4°C and dehydrated in a graded series of ethanol, with a 2h incubation step with 2% uranyl acetate in 70% Ethanol at 4°C. Samples were further dehydrated and embedded in epon resin. Thin (70-nm) sections were stained with uranyl acetate and lead citrate and examined by transmission electron microscope (Zeiss EM 912). Images were acquired with a digital camera Gatan Bioscan 792, using the Digital Micrograph software.

### In situ hybridizations and measurement of myoblast orientation

Embryos were collected in PBS and fixed in 4% PFA. In situ hybridizations were performed with a MyoD RNA probe on whole mount E12.5 embryos, according to previously published procedures [95]. In order to assess the orientation of myoblasts, the CM muscle sheet was dissected and flat mounted, after completion of the ISH procedure, for high magnification imaging with a Zeiss Axioplan. For each muscle, three areas in stereotyped positions of the CM (3 positions in which the main chain direction made a 10°, 45°, and 70° angle with the DV axis, respectively) were imaged at 63X resolution. Scoring myoblast direction was done using AxioVision image software (Zeiss Imaging). For each picture, three to four chains were outlined. For each cell, an angle between the closest outlined chain and the nucleus direction was measured. Every cell for which the nucleus was visible was assigned such an angle. The distribution of angles was thus determined for each embryo side (two CM muscles per embryo), by defining angle ranges of 10°, and determining the percentage of cells showing an angle in the given angle range. This distribution was averaged between 3 wild types embryos sides (n = 3), and 5 *Fat1^LacZ/LacZ^* embryo sides (n = 5).

### Western blot analysis

Tissue extracts were prepared in EBM buffer (20 mM Tris–HCl pH 7.5, 150 mM NaCl, 1% Triton, 5 mM EDTA, 5 mM EGTA, 10% glycerol) supplemented with protease inhibitors [95]. To enrich the lysates in membrane associated proteins, lysates were lectin-purified by incubation with Lectin-sepharose beads. For immunoblotting, 50 µg of protein extracts were separated by SDS–PAGE using 3–8% gradient gels (Invitrogen), blotted onto nitrocellulose membrane and detected with specific antibodies. Immunoblots were revealed by ECL (Amersham).

### Anti-FAT1 antisera production and purification

We first constructed a GST-FAT1 fusion protein, containing the C-terminal part of the intracellular domain of mouse FAT1 (from aa 4451 to 4588; an epitope entirely contained by exon 28, and comprising approximately one third of the cytoplasmic domain), in PGEX2 vector. Serum was collected from two rabbits immunized with the GST-FAT1 fusion protein (Rb 1465 and 1464). Antibodies were affinity purified from the two antisera, using the same GST-FAT1 (GST-Fat1-aa4451-4588) fusion protein, loaded on Affi-gel 15 support in poly-prep chromatography columns, following the manufacturer's instruction (Biorad).

### RNA extraction and Real-Time quantitative Polymerase Chain Reaction (RT-PCR)

In both mouse and human samples, total RNA was isolated using Trizol reagent (Gibco, BRL). RNA was resuspended in 100 µl DEPC-treated H2O and quantified by spectrophotometry; samples used for RT-PCRs had a 260/280 absorbance ratio greater than 1.8. cDNA was synthesized from 1 µg of total RNA using Superscript III (Invitrogen) or the First Strand cDNA Synthesis Kit (Fermentas RevertAid: K1622) and random oligonucleotides.

In mouse RNA samples, expression levels of *Fat1*, *Creatine kinase B* (*CKB*) or *HPRT* were determined by semi-quantitative and/or quantitative RT-PCRs using real-time sybrgreen PCR assay (life technologies), using the following primer sets. *Fat1* Primer set exons 20–21 (product size: 511 or 525 bp), 5′ CCA CGC GGT TGT CAT GTA CG 3′ (exon 20-Fw), and 5′ TCC AGT AGG CGA GGG ATT GC 3′ (exon 21-rev). *Fat1* Primer set exon 6–8 (product size: 545): 5′ AAG CCC CTT GAT GCA GAA CA 3′ (exon6-Fw); 5′ TCA GCG TTC CTC CCT TTG TC 3′ (exon8-rev). *Fat1* primer set exons 24–25 (product size 142 bp) 5′ TGC TGT CTG TCA GTG TGA CTC AGG C 3′ (exon 24-Fw); 5′ GAG AGG CAT CCT CAC AGT GCT TCC C 3′ (exon 25-rev); Fat1 primer set exons 26–28 (product size varies according to splice variants expressed; 3 products are observed:268 bp, 304 bp; and 330 bp) 5′ CGC TTA GCT CCT TCC AGT CAG AGT CC 3′ (exon 26-Fw); 5′ GGG TGG GTG TAT GGA CTC GAA CTG G 3′ (exon 28-Rev); *HPRT* primer set: HPRT-fw: 5′ CAC AGG ACT AGA ACA CCT GC 3′ HPRT-rev: 5′ GCT GGT GAA AAG GAC CTC T 3′. *Creatine kinase B-type* (*CKB*); CKB-Fw: 5′ ACG ACC ACT TCC TCT TCG ATA A 3′; CKB-rev: 5′ TTT TCA GTG TCA GCA ACA GCT T 3′. For qPCR experiments, the *HPRT* gene was used as endogenous reference gene to normalize the data across all samples. For each gene examined, primers were chosen at the junction between two exons, to distinguish by their size the RT-PCR products from the genomic DNA PCR products. For *Fat1* primer sets, sizes expected from genomic PCR amplicons, in case of genomic DNA contamination, have been indicated on Figures S4 and Figures S5).

Expression of the human *FAT1* gene was monitored by a real time quantitative RT-PCR method using TaqMan gene expression assay reference number Hs00170627_m1 targeting the 5′ part of the *FAT1* sequence (Applied biosystem), or using real-time sybrgreen PCR assay (Roche) (see primers below). The ubiquitous *beta-glucuronidase* (*GUS*), was used as endogenous reference gene to normalize the data across all samples. *FAT1* primers were chosen at the exon2-3 junction: forward primer: 5′- CAT TAG AGA TGG CTC TGG CG-3′; reverse primer: 5′- ATG GGA GGT CGA TTC ACG-3′). (Fw GUS: 5′-CTC ATT TGG AAT TTT GCC GAT T-3′; Rev *GUS*: 5′- CCG AGT GAA GAT CCC CTT TTT A-3′). Primers used for other muscle genes: *DHPR*-Fw: 5′- CGC AAC TGG TGG GTT GCC AGC-3′; *DHPR*-Rev: 5′- GGC CCA TCC TCC AGC AAC GC -3′; *MURF1*-Fw: 5′- CTT GAC TGC CAA GCA ACT CA -3′; *MURF1*-Rev: 5′- CAA AGC CCT GCT CTG TCT TC -3′; *DYSF*-Fw: 5′- GAA GCC AAG GTC CCA CTC CGA -3′; *DYSF*-Rev: 5′- CAG GCA GCG GTG TGT AGG ACA -3′; *Calp3*-Fw: 5′- TCT CTT CAC CAT TGG CTT CGC -3′; *Calp3*-Rev: 5′- TGC TGC TTG TTC CCG TGC -3′; *B2M*-Fw: 5′- CTC TCT TTC TGG CCT GGA GG -3′; *B2M*-Rev: 5′- TGC TGG ATG ACG TGA GTA AAC C -3′; *γSARC*-Fw: 5′- CGA CCC GTT TCA AGA CCT TA-3′; *γSARC*-Rev: 5′- CCT CAA TTT TCC CAG CGT GA -3′. Similar results were obtained with two other normalizing genes (*β-2-microglobulin* (*B2M*) or the human acidic ribosomal phosphosprotein (*PO*)).

Each experiment was performed in triplicate and repeated at least three times against age matched unaffected foetuses used as controls (see Figure S6A). For quantitative RT-PCR experiments, relative quantities of RNA expression were calculated using the comparative cycle threshold (ΔΔCt) method [96] and were normalized with GUS RNA levels as endogenous reference gene. Briefly, the fold change of RNA expression levels was calculated by the equation 2−ΔΔCt, where Ct is the cycle threshold. The cycle threshold (Ct) is defined as the number of cycles required for the fluorescent signal to cross the threshold in qPCR. ΔCt was calculated by subtracting the Ct values of the endogenous control (*GUS*) from the Ct values of the RNA of interest (*FAT1* or control muscle genes, such as *DHPR*, *MURF1*, *DYSF*, *Calp3*, *γ-Sarcoglycan*). ΔΔCt was then calculated by subtracting ΔCt of the sample used as control from the ΔCt of FSHD biopsies.

### Repression of *FAT1* by DUX4-fl

Human primary myoblasts unaffected by muscle disease were infected with lentivirus carrying either *DUX4-fl* or *GFP* as control for 24 hr. RNA was extracted with Qiagen RNeasy kit, DNAse'd with Ambion Turbo DNAse and reverse transcribed with Invitrogen SuperScript III according to manufacturers' instructions. Real time quantitative PCR was performed with the following primers: *FAT1*-f 5′ – GGA AAG CCT GTC TGA AGT GC - 3′; *FAT1*-r 5′ – TGT ATG TCC GGC AGA GGA AC -3′; *RPL13a*-f 5′ – AAC CTC CTC CTT TTC CAA GC - 3′; *RPL13a*-r 5′ – GCA GTA CCT GTT TAG CCA CGA - 3′. *FAT1* values were normalized to the internal standard *RPL13a* and expressed as percent relative to control condition.

### Chromatin immunoprecipitation assay

ChIP assays were performed on chromatin from fetal muscle biopsies (tissue samples obtained as described above) using the Magna A ChIP kit (Millipore/Upstate). For chromatin preparation, muscle samples (∼50 mg) were weighed, cut in small pieces, and cross-linked with 1.5% paraformaldehyde for 10 minutes, the cross-linking reaction being stopped by addition of glycin. Nuclei were extracted from the tissue samples by using a 2 ml dounce tissue grinder and the kit's cell lysis buffer. Chromatin was then sheared by sonication and quantified after DNA extraction. Immunoprecipitations were performed on 5 µg of Chromatin, with 3 µg of the following antibodies: anti-H3K4me3 (17–614, Millipore), anti-H3K27me3 (07–449, Millipore), and anti-H3K4me1 (ab8895, Abcam), following the ChIP kit instructions, using _proteinA-conjugated mareferredbeads. Immunoprecipitaded material was then washed, cross linking was reversed with proteinase K at 56° for 2 h, and DNA was extracted. The presence of individual regulatory regions in immunoprecipitated chromatin was analyzed by qPCR using sybr green (Invitrogen) on a Biorad CFX96 apparatus. Relative quantities of each chromatin bound fragment expression were calculated using the comparative cycle threshold (ΔΔCt) method again [96] and were normalized either relative to the amount of input DNA (in the same amount of chromatin before immunoprecipitation, quantified with the same PCR), or with levels of the promoter region of a normalizing genes GUSB.

Oligonucleotides for *FAT1* Promoter are: *FAT1*_P1_Fw: 5′ CTT AAG TTT GCC CTG GTC GGA AGC C 3′; *FAT1*_P1_Rev: 5′ AAA GTC CTC GGC AGC TCC GTG ATC C 3′; Oligonucleotides for the 17/18 intronic enhancer were: *FAT1*_inton17_Fw: 5′ gga gtg ggg agg agg gaa gag tgg g 3′; *FAT1*_intron_Rev: 5 ctt ccc tct tgc tct tct tct agc c 3′. Primers for the normalizing *GUSB* promoter were: *GUSB*_E/P_Fw: 5′ AGA GGA TGT AGA CCA GGC AAA AGC C 3′; *GUSB*_E/P_Rev: 5′ TAG AGG ACA GGA CAT GAC ATC AGG C-3′. All sequences were selected based on the Encode ChIP tracks on the UCSC browser (available with the base genome Human Mar. 2006 (NCBI36/hg18)).

### Genomic DNA array screen for microdeletions in FSHD patients

DNA was hybridized on a Nimblegen HD2.1 genomic array consisting of 135,000 probes targeting the 4q35.2 genomic region. Probes were designed with a spacing of 10 bp between consecutives probes in the exonic/intronic regions and 100 bp in the intergenic regions. Labeling of DNA and hybridization was based on the Nimblegen protocols. Arrays were scanned with the MS200 scanner (Roche) and the acquired paired files were analyzed using the CGHweb algorithms [97]. To visualize the deletion/duplication events, coordinates were formatted as a bed file and added in the custom tracks of the genome browser (genome.ucsc.edu). Patients that had CNVs in the intron 16–17 area are refered to with the number corresponding to their number in the patient summary table (Table S1).

A first PCR validation was performed using primers flanking the deleted area. This approach is expected to yield a PCR product of approximately 1500 bp from a control locus, and a smaller size product in the case of the deleted allele, the deletions being approximately 1 kb long. Primer sequences are: Del-Fw: 5′- CCT TCA CCT GCA GTA AG-3′; Del-Rev: 5′- CTA GGA TTC CTA AGA GC -3′. This approach led to validate the presence of both the 1500 band and the smaller band in the three independent patients (numbers 11, 12 and 25), plus patient 13 (sibling of 12), as carrying a deleted allele as well. Moreover, 20 unaffected controls were tested with this method and only yielded the control 1500 bp band, indicating the absence of deleted alleles.

Further validation of the deletion was performed by quantitative PCR, comparing the relative amount of PCR products scanning the deleted area, using as reference a PCR product outside the considered zone, a method used to quantify copy number variations [96], [98]. All DNA samples, whether from healthy controls or from FSHD patients, were normalized with the ADORA Reference PCR, against one healthy control DNA used as standard DNA (set to 100%). The reference primers are as follows: chr17p12 : ADORA2B-2F: 5′-GTC ACT CTT TTC CAG CCA GC-3′ ; ADORA2B-2R:5′-AAG TCT CGG TTC CGG TAA GC – 3′. The primers corresponding to the deleted area were as follows: qPCR-primer-1: 5′ – GCA ACA GAG GCC AAT GGA AA – 3′; qPCR-primer-2: 5′ – CTG AAA AGA TTT CAG GTT ACA CGC T – 3′; qPCR-primer-3: 5′ – TTC GGT AAG ATG GGA GCA GCC TTC C - 3′; qPCR-primer-4: 5′ – GGT CCT GAC AAG CTA ATC CTG AGG G - 3′; qPCR-primer-5: 5′ – GGA GTG TGG TGT GTT CTA GGT TAT GG – 3′; qPCR-primer-6: 5′ – AGC AGA CAA GAG CAC AAG GCA TTT C – 3′; qPCR-primer-7: 5′ – GGA ACA CAG CCA AAT CTA TAT GGG – 3′; qPCR-primer-8: 5′ – TCT TCC TCC TCA CAC TCC CTT TC – 3′; qPCR-primer-9: 5′ – CCT GGG CAA TGA GTG TAA CTC C – 3′; qPCR-primer-10: 5′ – CCA ACC TCC TCC CTA CTC CAC TT -3′; qPCR-primer-11: 5′ – CCA GTG GCA GCA GGT CTG ATT AAG C - 3′; qPCR-primer-12: 5′ – GGG AAA CGT AGA ATT CAA GAA GTC GC - 3′ (primers numbered as in Figure 10A and Figure S16A).

### Assay of hypomethylation status at *D4Z4* repeats

Among contraction-independent cases, a large proportion, referred to as FSHD2, harbours hypomethylated *D4Z4* [63]–[64], while others do not and are expected to carry unrelated causal abnormalities. Hypomethylation was assessed for 8 patients as indicated in the patient summary table, by digesting genomic DNA, with BlnI, CpoI and Eco91I, and hybridizing the southern blot with the p13E-11 probe, as described [64]. Numbers indicated represent the percentage of methylated proximal D4Z4 unit at chromosome 4q35.

### Statistical analysis

Results were expressed as the mean ± s.e.m. Statistically significant differences were assessed by unpaired t-Student test, or Mann-whitney test for non-Normally distributed data, X^2^ test or Fischer tests (for linkage studies), calculated with the StatEL add-in program to excel. The Kaplan-Meier plot was made with the StatEL add-in program to excel, and P value was calculated with the logrank test. * indicates P value<0.05; ** indicates P value<0.001.

## Supporting Information

Figure S1 *Fat1-LacZ* expression. (A) *Fat1^LacZ/+^* E10.5; E11.5 and E12.5 embryos stained with X-Gal to reveal β-galactosidase activity. The dotted areas are magnified in Figure 1C. The hotspot of expression in/around the Cutaneous Maximus (CM) is indicated with a red arrow. (B) *LacZ* expression in *Fat1^LacZ/+^* embryos faithfully reproduces *Fat1* expression as seen by in situ hybridization on transverse sections of E12.5 embryos in equivalent positions (upper thoracic). Positions of the CM, *Latissimus Dorsi* (LD) and *Trapeze*. (C) X-Gal staining of an E12.5 embryo carrying the *MLC3F-2E* (*LacZ*) transgene, showing the pattern of muscle differenciation.
(TIF)

Figure S2 Myoblast orientation phenotypes in non-CM scapular belt muscles of *Fat1^LacZ/LacZ^* embryos. Whole mount in situ hybridization with a *MyoD* RNA probe on wild type (A, C, E) or *Fat1^LacZ/LacZ^* (B, D, F) E12.5 embryo. (A, B) Low power magnification micrographs showing a side view of shoulder area. Anterior is to the left, dorsal is to the top. (C, D) Higher power magnification micrographs showing an enlargement of the corresponding boxed areas in (A and B), respectively. *Fat1^LacZ/LacZ^* embryos present numerous dispersed myoblasts in ectopic positions in the shoulder area, either as individual cells (red arrows), or clustered and forming ectopic muscles (orange arrows). (E, F) Higher (x63) magnification views of the corresponding boxed areas within the *trapezius* muscles in (C and D) showing misoriented myoblasts in *Fat1^LacZ/LacZ^* embryos. Scale bars: (A–B) 0.5 mm; (C, D) 50 µm; (E, F) 10 µm.
(TIF)

Figure S3 Targeted conditional deletion of FAT1 transmembrane domain. (A) Strategy used to generate the conditional allele. 1- Top: genomic organization of the *Fat1* locus around the targeted area. 2- Targeted *Fat1* locus, in which exons 24 and 25 (the latter containing the transmembrane domain) were flanked by *LoxP* sites (*Fat1^Fln^* allele). The locus also contains a pgk-neo selection cassette, itself flanked by *LoxP* sites (yellow triangles) and by FRT sites (blue triangles, for later removal of the *pgk-neo* cassette only). An external probe (red bar) was used to identify recombinant ES clones by Southern blotting. The sizes of the NcoI restriction fragments are indicated.3- *Neo* cassette removal is permitted by FRT-mediated excision of the neo cassette, which is flanked with both *FRT* (blue) and *LoxP* (yellow) sites. This generates a *Fat1^Flox^* allele, in which exons 24–25 are flanked on the 5′ side with one LoxP site, and on the 3′ side with one leftover FRT site, followed with a LoxP site. 4- CRE-Recombined *Fat1* locus: Genomic organisation of the targeted *Fat1* locus after cre-mediated excision of the entire fragment comprised between loxP sites, including exons 24–25 and the neo cassette. This new recombined allele is referred to as *Fat1^ΔTM^* allele. Primers indicated (1 to 7) are the ones used for genotyping by PCR. ES screening primers are given in the method section. (B) Abnormal shape of the Cutaneous Maximus in *Fat1^ΔTM/ΔTM^* embryos. Flat mounted preparations of dissected skeletal muscle groups from E13.5 and E18.5 control and *Fat1^ΔTM/ΔTM^* embryos, carrying the *MLC3f-2E* transgene, in which differentiated skeletal muscle cells are revealed by X-gal staining. Analysis of skeletal muscles confirms the reduced and misshaped CM at E13.5. The *MLC3F* transgene also reveals the presence of disoriented muscle cells in the forming CM at higher magnification. The shape of CM from E18.5 embryos is shown on the right. (C) Kaplan-Meier plot showing the probability of survival of *Fat1^ΔTM/ΔTM^* mice. Most (70%) *Fat1^ΔTM/ΔTM^* mice die between postnatal days P0 and P1, and less than 15% of *Fat1^ΔTM/ΔTM^* mice survive beyond 3 months after birth.
(TIF)

Figure S4 Selective and asymmetric muscle shape abnormalities in E14.5 *Fat1^ΔTM/ΔTM^* embryos. Skeletal muscle groups were visualized in E14.5, wild type and *Fat1^ΔTM/ΔTM^* embryos carrying the *MLC3f-2E* (LacZ) transgene, by X-gal staining. (A) Low magnification images showing the entire embryos. The drastically reduced length and density of the CM muscle is also visible at that stage. (B) high magnification views of hindlimb shank musculatures, showing no obvious shape differences, in particular in the *tibialis anterior* muscles, at that stage. Muscle name abbreviations: bf: *biceps femoris*; CM: *cutaneous maximus*; edl: *extensor digitorum longus*; pd: *peroneus digitorum*; pl: *peroneus longus*; ta: *tibialis anterior*. (C) Illustration of asymmetry of muscle shape abnormalities observed in two *Fat1^ΔTM/ΔTM^* embryos, by showing their respective left and right sides. All red arrows point to shape abnormalities that are different between the two sides.
(TIF)

Figure S5 Residual expression of *Fat1* RNAs in the *Fat1^LacZ/LacZ^* hypomorphic allele. In the *Fat1^LacZ^* insertion allele (ES line name *Fat1^KST249^*), multiple copies of a secretory-trap vector [41]–[42] were inserted in tandem downstream of the first exon of the *Fat1* gene (Figure S3A). As a result, the main product of the *Fat1* locus is a fusion protein of 291 kDa, including the first exon of *Fat1* (the first 8 cadherin domains), in frame with the exogenous transmembrane and beta-geo fusion protein (Figure S2A). (A, top) Schematic representation of the gene trap vector and its insertion point in the mouse *Fat1* locus. Precise content of the gene-trap vector has been described previously [41]–[42]. With its splice acceptor site, the depicted cassette behaves as an exon. Following (in blue), is an element encoding a transmembrane domain, and a beta-geo fusion reporter (in frame fusion of the β-galactosidase and the neomycin resistance gene). The ES selection procedure ensures that this reporter cassette is in frame with the preceding exon (exon 1 of mouse FAT1). The resulting FAT1-β-gal fusion is a transmembrane protein depicted in Figure 2A. This protein is recognized by antibodies raised against an epitope of FAT1 mapping in exon 1 (Fat1-1869; Figure 2B), but not by an antibody raised against a downstream epitope in the extracellular domain (Fat1-23882; Figure 2B). (A, bottom) Representation of several possible RNA products of the *Fat1* gene in mouse as they appear proposed by Ensembl as Ensembl-Havana and as EST-based gene products, respectively. At least three alternative sites of transcription initiation were identified, two of which located downstream of KST249 integration site. (B) RT-PCR analysis of *Fat1* transcripts in wild type and *Fat1^LacZ/LacZ^* mice, using primers matching exons 6–8 of mouse *Fat1*, and *HPRT* as control RNA. RT-PCRs were performed on RNA extracted from kidneys. mRNA containing *Fat1* exons 6–8 in kidneys are absent in adult *Fat1^LacZ/LacZ^* mice compared to control. (C) RT-PCR analysis of *Fat1* transcripts in wild type and *Fat1^LacZ/LacZ^* mice, using primers matching exon 20-21 junction of mouse *Fat1*, *Creatine kinase b (CKB)*, and *HPRT* as control RNA. RT-PCRs were performed on RNA extracted from *rhomboid* and CM muscles. Residual *Fat1* mRNA levels containing exons 21–22 in *Fat1^LacZ/LacZ^* mice inversely correlate with phenotype severity. In all cases, primers were chosen in two consecutive exons, so that the size of the amplicon resulting from cDNA and from any potential genomic DNA contamination would result different (sizes indicated). The band sizes shown are those resulting from cDNA amplification, and cannot be genomic DNA contamination. Animals used for RNA extractions were 10 week old littermates, a wild type mouse of 23 g, and 2 cases of *Fat1^LacZ/LacZ^* mice: one presymptomatic (with only slightly reduced weight as compared to wild type (20.4 g)), and one with strongly impaired growth and with 15% weight loss (11 g when sacrificed). Levels of residual Fat1 RNA differ between *Fat1^LacZ/LacZ^* mice, but also between muscles in a given mouse. The lowest levels of *Fat1* correlate with the most severe phenotype, as assessed by body weight, or by levels of *CKB*, known to be elevated in dystrophic muscles. (D) In situ hybridization with an RNA probe corresponding to the 3′UTR of Fat1 on whole mount spinal cords from E12.5 *Fat1^LacZ/+^* (left) and *Fat1^LacZ/LacZ^* (right) embryos. Results show that *Fat1* RNA levels are reduced in most but not all cells within the *Fat1*-expression domain.
(TIF)

Figure S6 Specific muscle wasting in presymptomatic *Fat1^LacZ/LacZ^* mice turns to generalized muscle wasting in symptomatic *Fat1^LacZ/LacZ^* mice. (A) Kaplan-Meier plot showing the relationship between disease span and age of onset, as evidenced through the comparison of the probability of onset (blue curve: probability of being presymptomatic) with the survival curve (probability of survival), on the same set of 49 *Fat1^LacZ/LacZ^* mice. *Fat1^LacZ/LacZ^* mice with early onset show short disease span, while the disease span can be long for mice with late onset. (B) Extent of muscle wasting in adult *Fat1^LacZ/LacZ^* mice at presymptomatic and symptomatic stages of phenotype progression. Muscle dissection in adult wild type (B_1,4_) and *Fat1^LacZ/LacZ^* mice at presymptomatic (B_2,5_) or advanced symptomatic (B_3,6_; 28% weight loss) stages reveals a pronounced reduction in volume and thickness of the CM muscle at both stages, while *Gastrocnemius* muscle in the hindlimb only show pronounced wasting at later stages or phenotype progression. Because of the tight association with the skin of its caudal, the CM cannot easily be dissected for weight measurements. In pictures in B1,2,3, this skin-associated caudal part was therefore arbitrarily cut, preventing fair assessment of its true volume. The CM was consequently not included in the relative muscle mass measurements in Figure 3D and Figure S4 (C). (B_7–9_) Myofibre diameter within the *Gastrocnemius* muscle was visualized on transverse sections from wild type and *Fat1^LacZ/LacZ^* mice at presymptomatic stage (B_8_) or at advanced symptomatic stage (B_9_, 20% weight loss), using antibodies against laminin. (C) Masses of dissected muscles of *Fat1^LacZ/LacZ^* mice at advanced (20–30% weight loss, n = 3) disease stages relative to age-matched controls (n = 6; average wild type weight defined as 100%). (D) Histological section of *Cutaneous maximus* muscles from adult wild type and *Fat1^LacZ/LacZ^* mice at presymptomatic stage, lightly stained with Toluidin Blue, showing reduced thickness (top), as well as reduced myofibre density, reduced myofibre diameter, and infiltration with connective tissue (bottom, higher magnification). Scale bars: (B_1–6_) 4 mm; (B_7–9_) 50 µm.
(TIF)

Figure S7 Reduced myofiber diameter and cellular infiltrations in affected muscles from adult *Fat1^ΔTM/ΔTM^* mice. (A) Cryosections of the *Trapezius Thoracis*, *Pectoralis Major*, *Tibialis anterior*, and *Soleus* muscles from adult wild type and presymptomatic *Fat1^ΔTM/ΔTM^* mice (4 months old) were stained with phalloidin-Alexa647 (purple), anti-Laminin (green), Collagen I (red), and DAPI (blue). The selected areas illustrate places in the *trapezius* and *Pectoralis Major* muscles with cellular infiltrations (yellow arrows) between myofibres, with two examples (right pictures) of perivascular infiltrations. In tibialis Anterior, the selected area contains nuclei of infiltrated cells surrounded with Collagen I-positive deposit (yellow arrow). (B) Average myofiber diameters were quantified in the analysed muscles, and presented as %age of the area of the corresponding Wild type muscle. Myofiber diameter is significantly smaller in *Trapezius* (** p<0.001), in *Pectoralis Major* (** p<0.001), and in *Tibialis anterior* (* p<0.01), but not in *soleus*.
(TIF)

Figure S8 Ectopic muscles variably connecting shoulder or humeral muscles, which are subject to loss of integrity at adult stages. Ectopic muscles (Ect.) were identified during dissection of shoulder or limb musculature from P3 *Fat1^LacZ/LacZ^* pups (A–C) and several adult *Fat1^LacZ/LacZ^* mice at presymptomatic (D–I) or advanced (J–L) stages of disease progression. Each ectopic muscle was first photographed in its original context (except rhomboids, where the dissection procedure makes it impossible), to visualise original attachment sites with other muscles, and at higher magnification, after dissection pinned on sylgard, without disconnecting the ectopic muscles from the limb or shoulder muscles they were connected to. (C, L) Toluidin counterstained semithin sections of the ectopic muscles seen in (A and B) and (J and K), respectively. Images show that myofibers properly assemble the contractile apparatus (C, P3) but display severe alterations of their histology at adult stages (L). LD: *latissimus dorsi*; Trp: *trapezius*; Tri: *triceps brachii*; s. sc: *subscapularis muscle*. Scale bars: (A) 2 mm; (B) 0,5 mm; (D) 6 mm; (E) 1 mm; (C, F) 20 µm.
(TIF)

Figure S9 Examples of NMJ denervation and atrophy in *Fat1^LacZ/LacZ^* mice. NMJs were visualized in wild type (A_1_–A_3_) and *Fat1^LacZ/LacZ^* (B_1_–D_3_) *rhomboid muscles* by immunolabelling AChR clusters with α-bungarotoxin (α-BTX, green), and nerve endings with neurofilaments (NF, red). Each synapse is shown with separate α–BTX-green (**_1_**) and NF-red channels (**_2_**) separately, and with a merge image of both channels (**_3_**). In wild type synapses, the NF-positive axon endplate overlaps with all circumvolutions of the bretzel-like shaped α-BTX-positive postsynaptic area. A portion of axon proximal to the NMJ is intensely stained with NF (white stars in all panels). By contrast, *Fat1^LacZ/LacZ^* synapses display reduced α-BTX-positive surface, with fragmentation, interruptions, swelling, or absence of the axon endplate, although the NF-positive axon (white starts) is still detectable.
(TIF)

Figure S10 Tissue-Specificity of *Pax3-cre* recombinase activity. (A, B) *Pax3-cre* activity was assayed by performing anti-YFP immunohistochemistry (rabbit anti-GFP antibody, Invitrogen) on cryosections of an E12.5 *Pax3^cre/+^; Rosa26^Lox-STOP-Lox-YFP/+^* embryo. (A) Section at Forelimb level, showing muscle masses in the limbs. The two magnified areas show the limb and spinal cord region. In the limb, besides its activity in muscles, *Pax3-cre* also leads to excision in neural crest derivatives including Schwann cells along the nerves. In the Spinal cord, *Pax3-cre*-derived lineage includes dorsal neural precursors and their derivatives mostly confined dorsally. Motor neurons in the motor columns do not express YFP, hence are not part of the *Pax3-cre* lineage. (B) a slightly posterior section shows efficiency of *Pax3-cre* activity in the *Cutaneous maximus* muscle. (C) Section of an E12.5 *Fat1^LacZ/+^* embryo stained with X-gal at a forelimb level comparable to that shown in (A), illustrating *Fat1* expression in the same muscle mass as that expressing YFP in the magnified area in (A), as well as in a cervical motor neuron pool, throughout the ventricular zone, and in multiple non muscle sites, including the vertebral bodies. (D) In situ hybridization was performed with antisense probes for *myoD* (top) and for *Fat1*-exon24/25 (the floxed exons) on alternate cryostat sections of a *Fat1^Fln/Fln^* and a *Fat1^Fln/Fln^; Pax3^cre/+^* E12.5 embryos, at a level slightly posterior to that shown in (B). In contrast to *Fat1* expression in the ventral neuroepithelium (vNE) or in the lung, which is preserved because it does these tissue-types do not derive from *Pax3*-expressing precursors, *Fat1* expression is reduced or abolished in *Pax3*-derived cell types, such as the dorsal neuroepithelium (dNE) or muscles (CM, LD and *spinotrapezius* are indicated).
(TIF)

Figure S11 *Fat1* RNA levels are mildly affected by the conditional strategy. (A) Scheme of the murine *Fat1* genomic locus, showing exon/intron structure, highlighting the area that has been floxed in the conditional allele, as well as the positions of primers that have been used for quantitative RT-PCR studies on mouse tissues. (B,C) RT-PCR studies were performed on RNA preparations from embryos of the indicated genotypes, to evaluate the impact of *Fat1* targeting on expression of *Fat1* mRNAs containing either the floxed exons (24–25), or the last C-terminal exons (26 to 28). (B) All PCRs were performed on cDNA preparations with or without reverse-transcriptases (+ or − RT), and were loaded on agarose gels to validate that the observed amplicons were specifically obtained from cDNA. In all cases, since primers were chosen in two different exons, and the sizes expected from amplicons from cDNA and genomic DNA are indicated. (C) quantitative PCRs were performed on cDNAs from wild type, *Fat1^ΔTM/+^*; *Fat1^ΔTM/ΔTM^*; *Fat1^Fln/+^* and *Fat1^Fln/Fln^* embryos, to measure the relative amount of RNA containing the floxed exons (24–25, top graph, blue), or the last C-terminal exons (26 to 28, bottom graph, red). Data for each genotype were averaged from 3 embryos, and HPRT was used as normalizing gene. (Top graph): As expected, expression of mRNAs containing exons 24–25 is abrogated in *Fat1^ΔTM/ΔTM^* embryos, and reduced by 50% *Fat1^ΔTM/+^* in embryos. The presence of a neo cassette in the conditional *Fat1^Fln^* allele exerts a mild effect on *Fat1* expression, visible through a 50% reduction of the exon 24–25 signal in *Fat1^Fln/Fln^* embryos. (Bottom graph): Expression of mRNAs containing the last exons is not abrogated by the constitutive deletion of exons 24–25, and respresents less than 50% in *Fat1^ΔTM/ΔTM^* embryos compared to wild-type. In contrast to upstream exons 24–25, expression of RNAs containing exons 26–28 is only moderately influenced by the neo cassette in *Fat1^Fln/Fln^* embryos. This suggests that the lowering observed in *Fat1^ΔTM/ΔTM^* embryos is a consequence of loss of *Fat1* function and indicates an autoregulation mechanism.
(TIF)

Figure S12 *Fat1* ablation in trunk premigratory muscle precursors under *Pax3-cre* reproduces the scapulohumeral muscle shape phenotypes of the constitutive mutants. Muscle anatomy was visualized at E14.5 (A, B) and E15.5 (C), by X-Gal staining in *Fat1^Fln/Fln^*; *MLC3F-2E* and *Fat1^Fln/Fln^*; *Pax3^cre/+^*; *MLC3F-2E* embryos. Mild phenotypes can be detected in *Fat1^Fln/Fln^* embryons in the face (reduced *occip. Frontalis* muscle, and *zygomatics*), and through appearance of misplaced muscle fibres between *Trapezius Cervicalis* and *Trapezius Thoracis*, frequently unilateral or asymmetric (red arrow). While *Pax3-cre* driven recombination in *Fat1^Fln/Fln^*; *Pax3^cre/+^*; *MLC3F-2E* embryos does not cause any worsening in muscle shape and size in the face, abnormalities can be seen in the scapulohumeral region, such as the appearance of an additional muscle, in an ectopic position reminiscent of that seen in *Fat1^ΔTM/ΔTM^* embryos, without insertion of its extremity between the *spinodeltoid* and the *Triceps brachii* muscles.
(TIF)

Figure S13 Residual FAT1 protein isoforms are produced in *Fat1^LacZ/LacZ^* and *Fat1^ΔTM/ΔTM^* mice. Residual FAT1 protein levels in *Fat1^LacZ/LacZ^* mice inversely correlate with phenotype severity. (A) Western blot analysis on Lectin-purified muscle protein extracts from 9 days old pups, comparing a wild type and two *Fat1^LacZ/LacZ^* cases with different phenotype severity, was performed using a previously characterized anti-FAT1-ICD antibody from ref [35]. (B, C) Western blot analysis was performed with: (B) total brain protein lysates from the same cases shown in (A), or with (C) brain and muscle protein lysates from wild type and *Fat1^ΔTM/ΔTM^* P0 pups. Membranes with were blotted with anti-FAT1 antibodies (Rb-1465). ERKs protein levels were used as loading controls (lower panels in A and B). Mutant *Fat1^LacZ/LacZ^* mice survive postnatally with variable phenotype severity (see Figure 3C). In the two examples shown here, both *Fat1^LacZ/LacZ^* mutants showed strong phenotypes since birth including impaired growth, with a milder case weighing 3.6 g and a severe case weighing 2.1 g (where reduced weight reflects phenotype) compared to their wild type littermates weighing 6.5 g in average.
(TIF)

Figure S14 Characteristics of human foetal FSHD1 and control cases. (A) Tables listing the FSHD1 foetuses (top) and control fetuses (bottom) from which biopsies were used for the present study. These tables indicate for each case, the ID symbol, the stage at which termination of pregnancy was performed (in weeks of amenorrhea), their sex, and for FSHD1 cases, the number of *D4Z4* microsatellite repeats. (B) Scheme representing the design of DNA probes and fluorophores used for genotyping FSHD patients by molecular combing, in combed genomic DNA as described in ref [94]. (C) Molecular combing genotyping results showing 3 different alleles of 4q35 were detected in the genome of one male individual, chimaeric carrier of an FSHD1 allele: One 4qB allele (blue, right side), one “normal” 4qA allele with a long D4Z4 strech (green), and one contracted 4qA allele (4qA*), qualifying as FSHD1. This individual was not diagnosed with FSHD, most likely owing to his degree of chimaerism, but was the father (family tree shown in (D) of one first child with severe, early onset FSHD, and one foetus diagnosed with FSHD as well through prenatal diagnosis (one of which was used in this study as F1;genotype shown in E). (D) Family tree showing the distribution of FSHD symptoms in this patient family. (E) Molecular combing genotyping of the F1 FSHD foetus, showing one 4qA* contracted FSHD1 allele inherited from the father, and one 4qB allele inherited from the mother (distinct from the father's 4qB).
(TIF)

Figure S15 qRT-PCR and immunohistochemistry data from foetal FSHD muscle. (A) Western blot analysis of Fat1 levels in muscle protein extracts from the 26 weeks old FSHD Foetus with 1.5 D4Z4 repeats (F1) and 2 age-matched control foetuses (C_1_ and C_2_) with the anti-FAT1-ICD antibody from ref [35]. (B) qPCR analysis of mRNA levels of several genes other than *FAT1* (shown in Figure 7) involved in muscle biology (*DHPR*, *γ-sarcoglycan* (*γSARC*), *MURF1*, *DYSF*) in quadriceps muscles of a 26 weeks old FSHD1 foetus (F1) harbouring 1.5 D4Z4 repeats in the 4q35 region (dark red bars), and a 16 weeks old FSHD1 foetus harbouring 4.3 D4Z4 repeats at 4q35 region (F2), respectively compared with age-matched control foetuses (blue bars). (C) Immunolocalization of FAT1 (Rb-1465 anti FAT1-ICD, green) and α-actinin (αact, red) in longitudinal sections from human quadriceps biopsies from a control (top) or and the FSHD1 (F2, bottom) foetus with 4.3 D4Z4 repeats. (D) Quantitative PCR shows that *DUX4-fl* downregulates expression of *FAT1* in human primary myoblasts. DUX4-fl or GFP (control) were expressed in unaffected muscle cells by lentiviral delivery. Data were normalized to internal standard *RPL13a* and represented as mean +/−SD of triplicates with control set at 100%.
(TIF)

Figure S16 Custom array CGH analyses of genome copy number changes in the 4q35.2 region. (A) Scheme of the genomic region in which CNVs were identified. The region represented is identical to that shown in Figure 10. The dotted line boxes represent positions of the PCR primers used for qPCR validation of copy number variants. The boxes in grey are those shown in Figure 10, the brown ones represent position of the primers for qPCRs shown in (D). (B) Screen copies of USCC browser lanes matching the position shown in (A), representing ENCODE-derived data (available on http://genome.ucsc.edu). The “layered H3K27Ac track”, shows enrichment of the H3K27Ac histone mark across the genome as determined in 7 cell lines by a ChIP-seq assay (the H3K27Ac histone mark is the acetylation of lysine 27 of the H3 histone protein). The lower tracks represent expanded image showing a chromatin state segmentation [67] for each of 7 of the nine human cell types, computationally integrating ChIP-seq data for nine factors plus input. The intron 16 ehancer appears labelled as exhibiting strong enhancer activity in HSMM cells (human skeletal myoblast muscle cells). (C) Genome copy number variation frequencies are plotted as a function of position in the same region as that shown in (A). Chromosome locations (NCBI36hg18 build) are indicated by numbers above graph. Negative values (log2ratio<−0.3) indicate frequencies of probes showing copy number decreases between DNA of patient 11 respect to a control DNA. The extent of deletion is of around 1 kilobase. (D) Copy number validation of the deletion by qPCR. The relative amounts of a PCR fragments obtained using primers couples indicated in (A), which amplification corresponds to the control allele (blue bars), were compared between a control patient DNA (with 2 copies of the normal allele), and three C.I.FSHD patients carrying one copy of a deleted allele (green bars); but also three additional c.i.FSHD patients (orange bars) that did not show copy number variations in the CGH experiments. Patients' numbers refer to the numbers indicated in Table S1. Data were normalized using an unrelated genomic fragment (Adora) as internal control. Patients 12 and 13 were both confirmed, with two Primer sets matching the deletion span (qPCR (1–2) and qPCR (3–4), corresponding primers shown in A), to carry half the amount of the normal allele compared to the control DNA, validating the deletion.
(TIF)

Table S1 Characterization of FSHD patients involved in the study. Information provided for each patient includes genetic and clinical characteristics, as well as raw results for each FSHD patient of qPCR measurement of copy number variants at 6 positions in the FAT1 locus, and of analysis of D4Z4 methylation (at the CpoI site, proximal repeat, 4q). STATUS indicates whether the diagnosis was FSHD1 or contraction-independent FSHD (c.i.FSHD), but also which cases were considered as carrying a deletion (Loss at any of the 6 positions considered). Patients are numbered as they appear in Figure 10.
(XLSX)
