# Supplementary figures and images for: Deregulation of the Protocadherin Gene FAT1 Alters Muscle Shapes: Implications for the Pathogenesis of Facioscapulohumeral Dystrophy
Source: PLoS Genet. 2013 Jun 13;9(6):e1003550. doi: 10.1371/journal.pgen.1003550 (PMC3681729; doi:10.1371/journal.pgen.1003550)

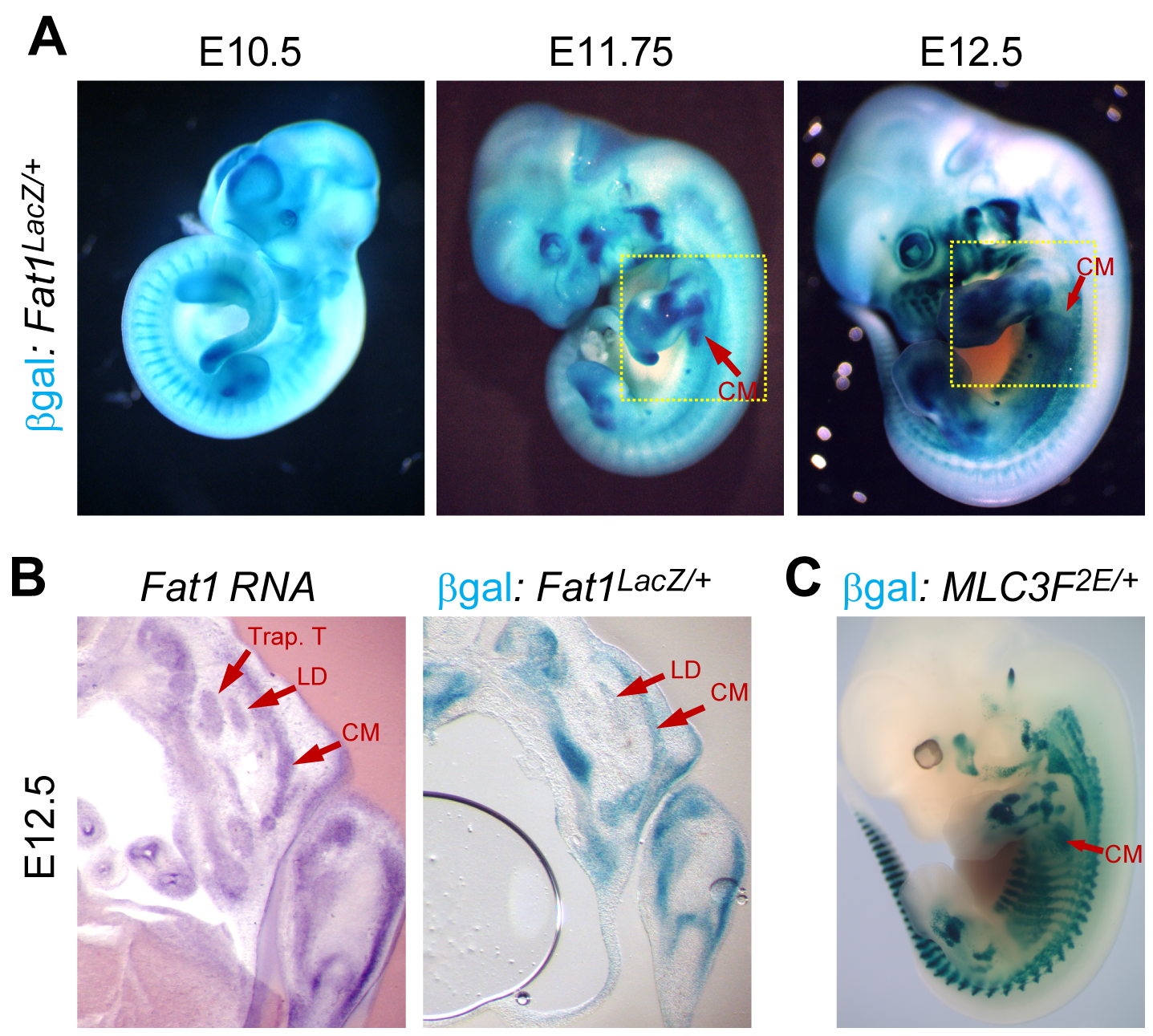

Supplement: Figure S1 — Fat1-LacZ expression. (A) Fat1LacZ/+ E10.5; E11.5 and E12.5 embryos stained with X-Gal to reveal β-galactosidase activity. The dotted areas are magnified in Figure 1C. The hotspot of expression in/around the Cutaneous Maximus (CM) is indicated with a red arrow. (B) LacZ expression in Fat1LacZ/+ embryos faithfully reproduces Fat1 expression as seen by in situ hybridization on transverse sections of E12.5 embryos in equivalent positions (upper thoracic). Positions of the CM, Latissimus Dorsi (LD) and Trapeze. (C) X-Gal staining of an E12.5 embryo carrying the MLC3F-2E (LacZ) transgene, showing the pattern of muscle differenciation. (TIF) [file pgen.1003550.s001.tif]

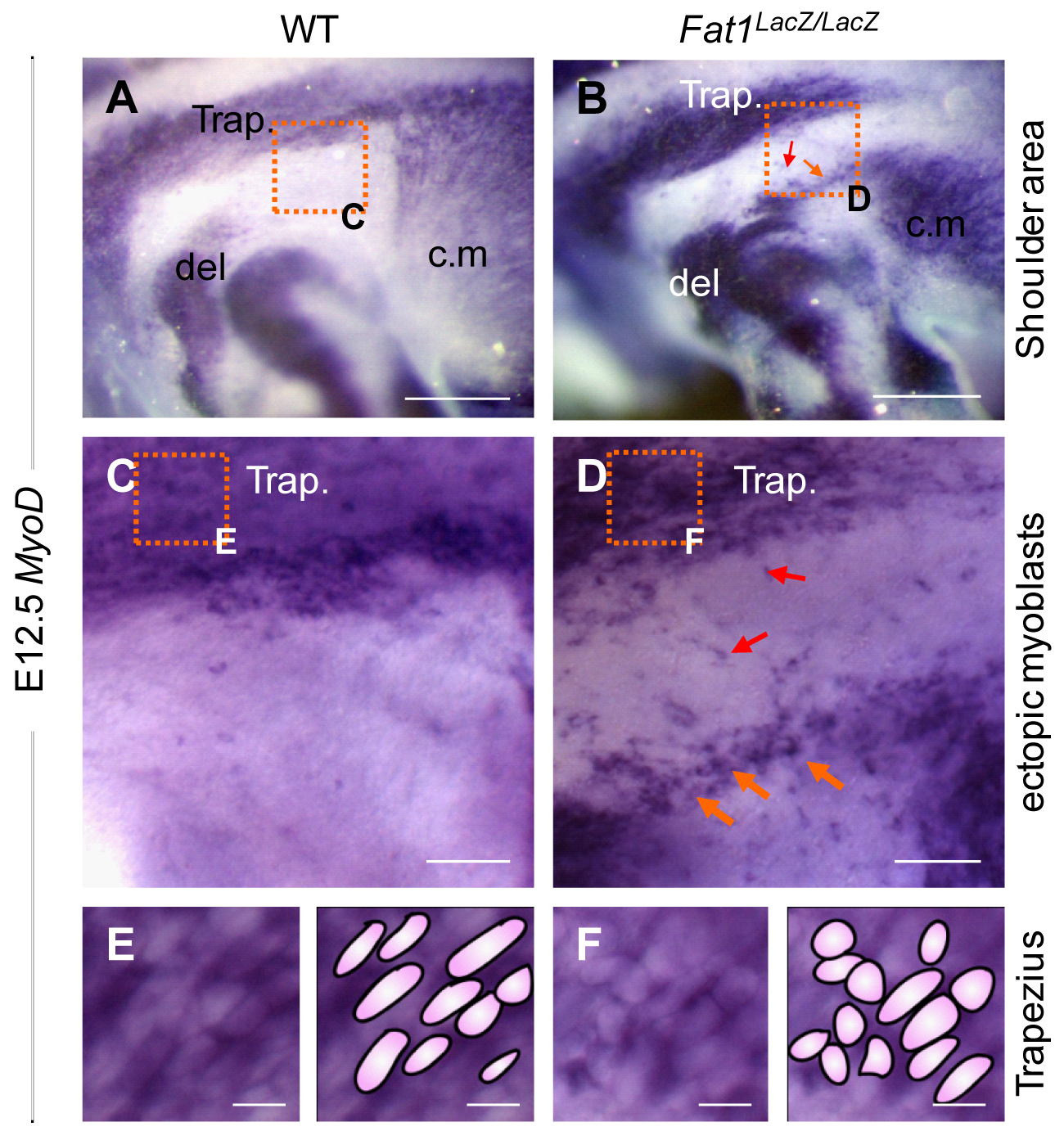

Supplement: Figure S2 — Myoblast orientation phenotypes in non-CM scapular belt muscles of Fat1LacZ/LacZ embryos. Whole mount in situ hybridization with a MyoD RNA probe on wild type (A, C, E) or Fat1LacZ/LacZ (B, D, F) E12.5 embryo. (A, B) Low power magnification micrographs showing a side view of shoulder area. Anterior is to the left, dorsal is to the top. (C, D) Higher power magnification micrographs showing an enlargement of the corresponding boxed areas in (A and B), respectively. Fat1LacZ/LacZ embryos present numerous dispersed myoblasts in ectopic positions in the shoulder area, either as individual cells (red arrows), or clustered and forming ectopic muscles (orange arrows). (E, F) Higher (x63) magnification views of the corresponding boxed areas within the trapezius muscles in (C and D) showing misoriented myoblasts in Fat1LacZ/LacZ embryos. Scale bars: (A–B) 0.5 mm; (C, D) 50 µm; (E, F) 10 µm. (TIF) [file pgen.1003550.s002.tif]

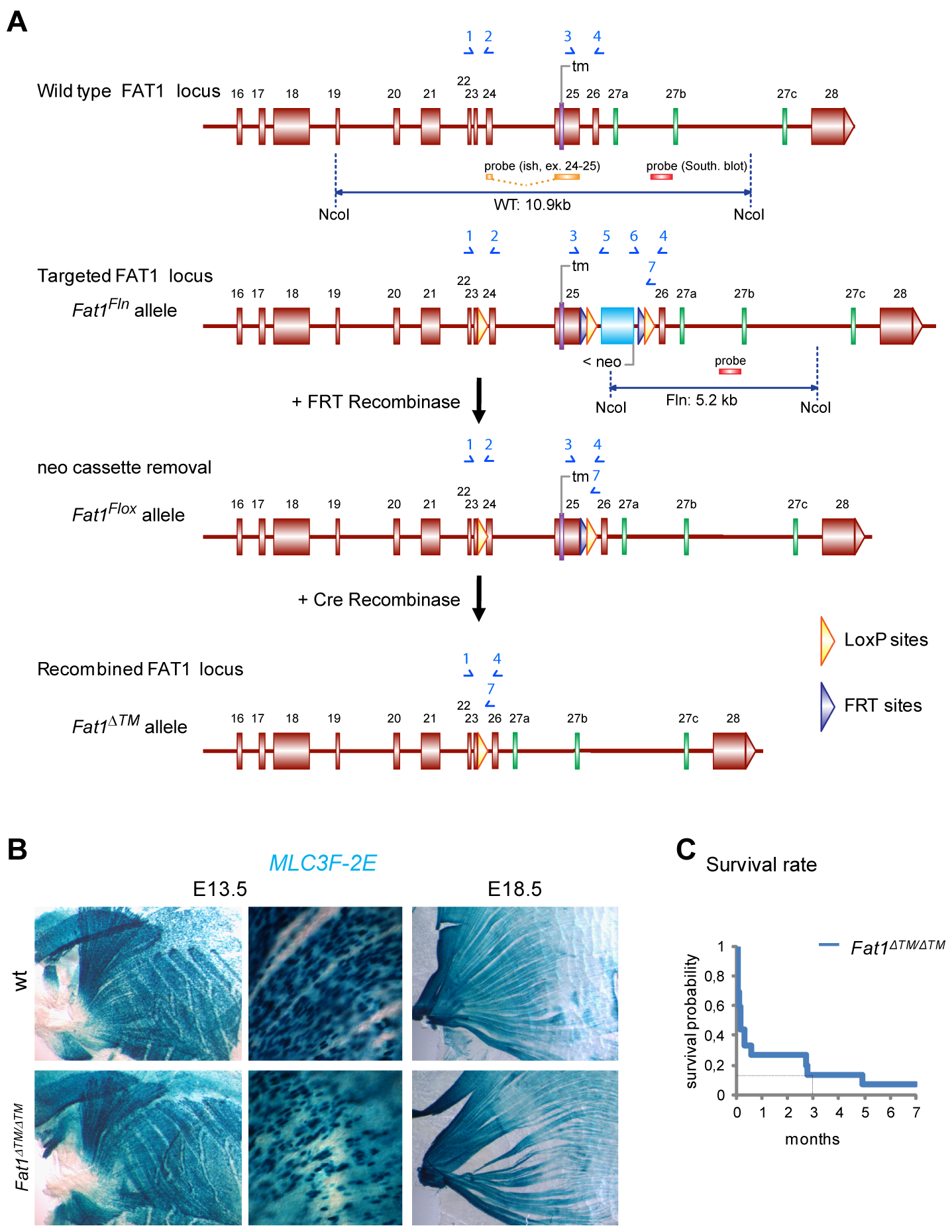

Supplement: Figure S3 — Targeted conditional deletion of FAT1 transmembrane domain. (A) Strategy used to generate the conditional allele. 1- Top: genomic organization of the Fat1 locus around the targeted area. 2- Targeted Fat1 locus, in which exons 24 and 25 (the latter containing the transmembrane domain) were flanked by LoxP sites (Fat1Fln allele). The locus also contains a pgk-neo selection cassette, itself flanked by LoxP sites (yellow triangles) and by FRT sites (blue triangles, for later removal of the pgk-neo cassette only). An external probe (red bar) was used to identify recombinant ES clones by Southern blotting. The sizes of the NcoI restriction fragments are indicated.3- Neo cassette removal is permitted by FRT-mediated excision of the neo cassette, which is flanked with both FRT (blue) and LoxP (yellow) sites. This generates a Fat1Flox allele, in which exons 24–25 are flanked on the 5′ side with one LoxP site, and on the 3′ side with one leftover FRT site, followed with a LoxP site. 4- CRE-Recombined Fat1 locus: Genomic organisation of the targeted Fat1 locus after cre-mediated excision of the entire fragment comprised between loxP sites, including exons 24–25 and the neo cassette. This new recombined allele is referred to as Fat1ΔTM allele. Primers indicated (1 to 7) are the ones used for genotyping by PCR. ES screening primers are given in the method section. (B) Abnormal shape of the Cutaneous Maximus in Fat1ΔTM/ΔTM embryos. Flat mounted preparations of dissected skeletal muscle groups from E13.5 and E18.5 control and Fat1ΔTM/ΔTM embryos, carrying the MLC3f-2E transgene, in which differentiated skeletal muscle cells are revealed by X-gal staining. Analysis of skeletal muscles confirms the reduced and misshaped CM at E13.5. The MLC3F transgene also reveals the presence of disoriented muscle cells in the forming CM at higher magnification. The shape of CM from E18.5 embryos is shown on the right. (C) Kaplan-Meier plot showing the probability of survival of Fat1ΔTM/ΔTM mice. [file pgen.1003550.s003.tif]

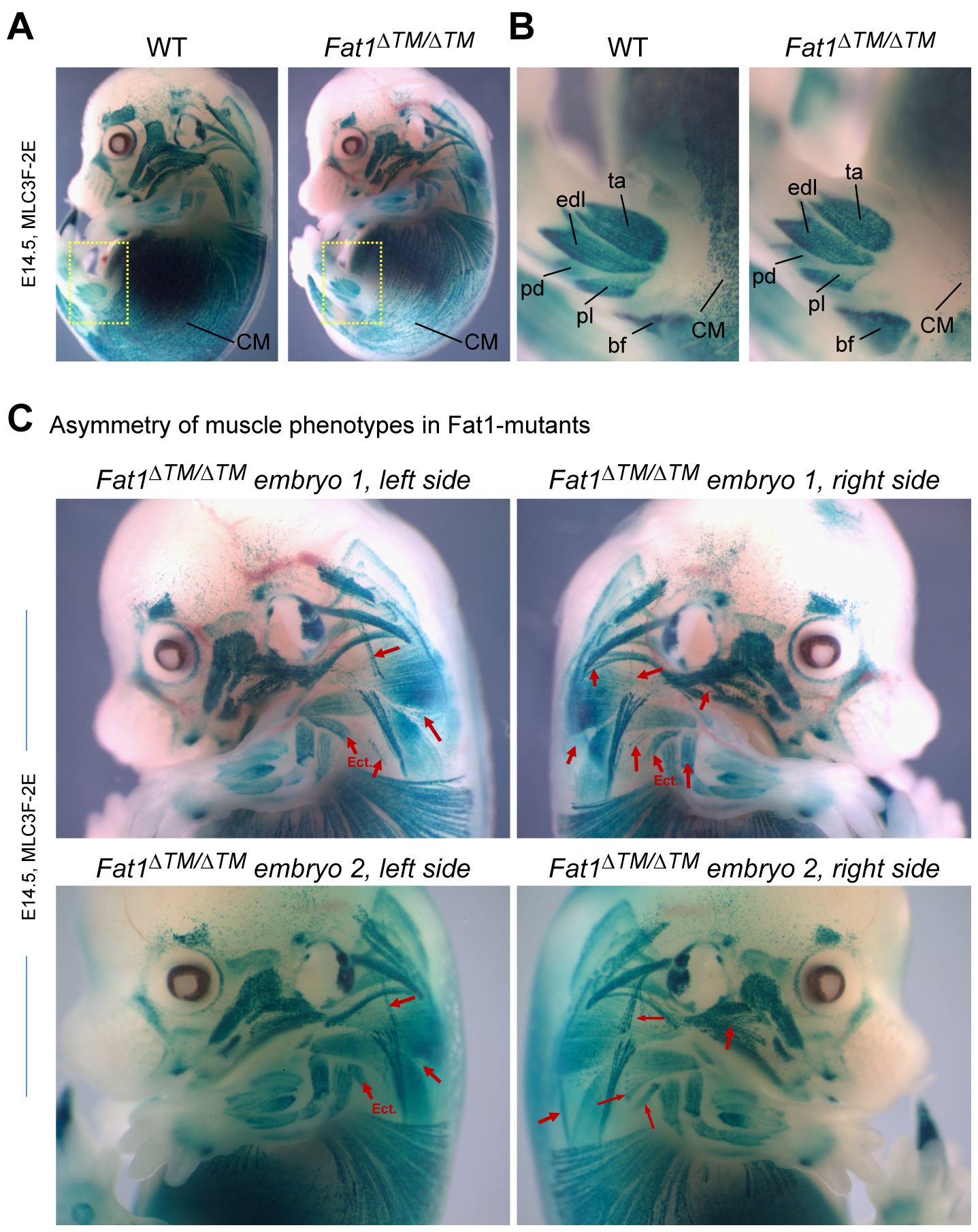

Supplement: Figure S4 — Selective and asymmetric muscle shape abnormalities in E14.5 Fat1ΔTM/ΔTM embryos. Skeletal muscle groups were visualized in E14.5, wild type and Fat1ΔTM/ΔTM embryos carrying the MLC3f-2E (LacZ) transgene, by X-gal staining. (A) Low magnification images showing the entire embryos. The drastically reduced length and density of the CM muscle is also visible at that stage. (B) high magnification views of hindlimb shank musculatures, showing no obvious shape differences, in particular in the tibialis anterior muscles, at that stage. Muscle name abbreviations: bf: biceps femoris; CM: cutaneous maximus; edl: extensor digitorum longus; pd: peroneus digitorum; pl: peroneus longus; ta: tibialis anterior. (C) Illustration of asymmetry of muscle shape abnormalities observed in two Fat1ΔTM/ΔTM embryos, by showing their respective left and right sides. All red arrows point to shape abnormalities that are different between the two sides. (TIF) [file pgen.1003550.s004.tif]

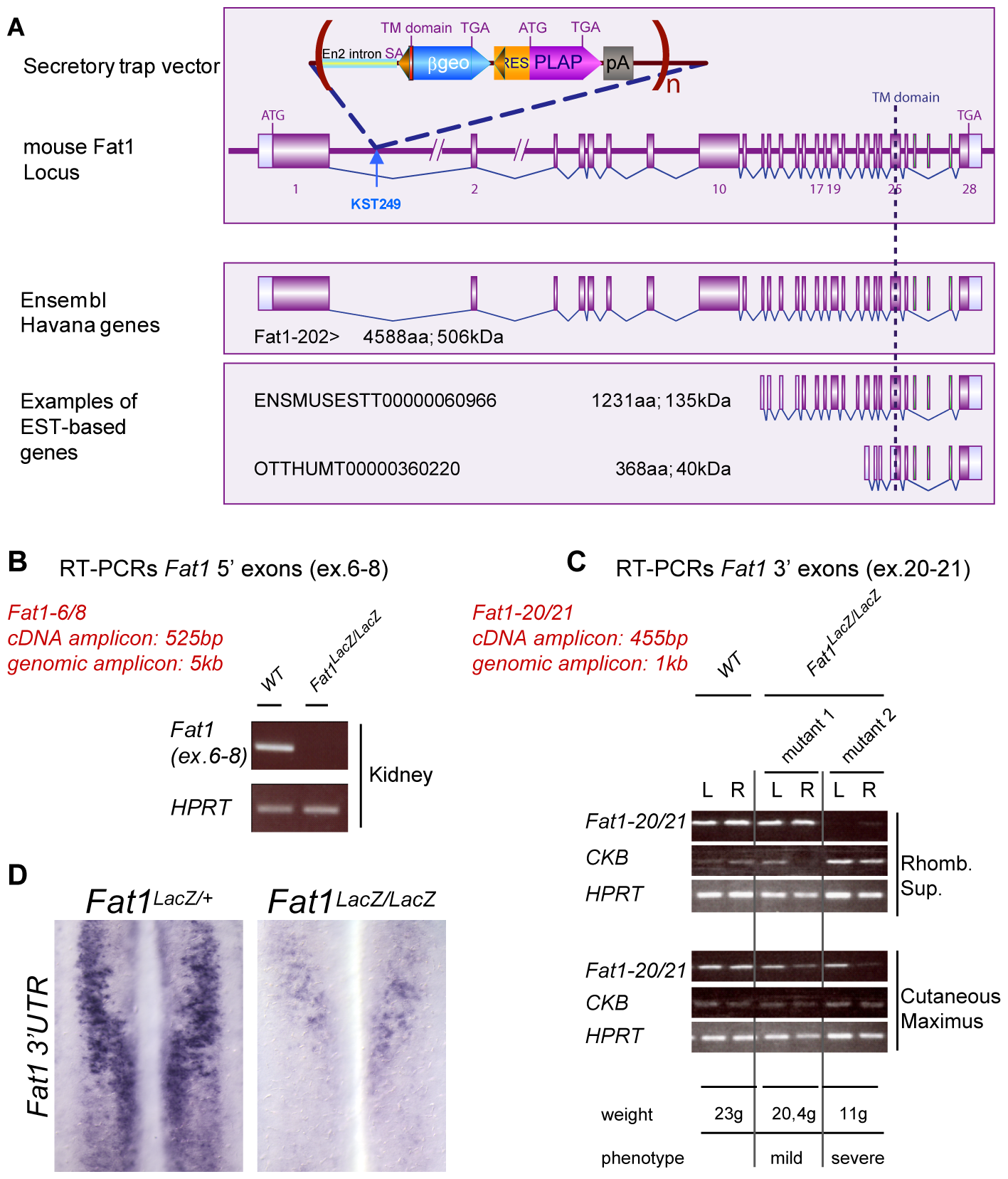

Supplement: Figure S5 — Residual expression of Fat1 RNAs in the Fat1LacZ/LacZ hypomorphic allele. In the Fat1LacZ insertion allele (ES line name Fat1KST249), multiple copies of a secretory-trap vector [41]–[42] were inserted in tandem downstream of the first exon of the Fat1 gene (Figure S3A). As a result, the main product of the Fat1 locus is a fusion protein of 291 kDa, including the first exon of Fat1 (the first 8 cadherin domains), in frame with the exogenous transmembrane and beta-geo fusion protein (Figure S2A). (A, top) Schematic representation of the gene trap vector and its insertion point in the mouse Fat1 locus. Precise content of the gene-trap vector has been described previously [41]–[42]. With its splice acceptor site, the depicted cassette behaves as an exon. Following (in blue), is an element encoding a transmembrane domain, and a beta-geo fusion reporter (in frame fusion of the β-galactosidase and the neomycin resistance gene). The ES selection procedure ensures that this reporter cassette is in frame with the preceding exon (exon 1 of mouse FAT1). The resulting FAT1-β-gal fusion is a transmembrane protein depicted in Figure 2A. This protein is recognized by antibodies raised against an epitope of FAT1 mapping in exon 1 (Fat1-1869; Figure 2B), but not by an antibody raised against a downstream epitope in the extracellular domain (Fat1-23882; Figure 2B). (A, bottom) Representation of several possible RNA products of the Fat1 gene in mouse as they appear proposed by Ensembl as Ensembl-Havana and as EST-based gene products, respectively. At least three alternative sites of transcription initiation were identified, two of which located downstream of KST249 integration site. (B) RT-PCR analysis of Fat1 transcripts in wild type and Fat1LacZ/LacZ mice, using primers matching exons 6–8 of mouse Fat1, and HPRT as control RNA. RT-PCRs were performed on RNA extracted from kidneys. mRNA containing Fat1 exons 6–8 in kidneys are absent in adult Fat1LacZ/LacZ mice compared to control. (C [file pgen.1003550.s005.tif]

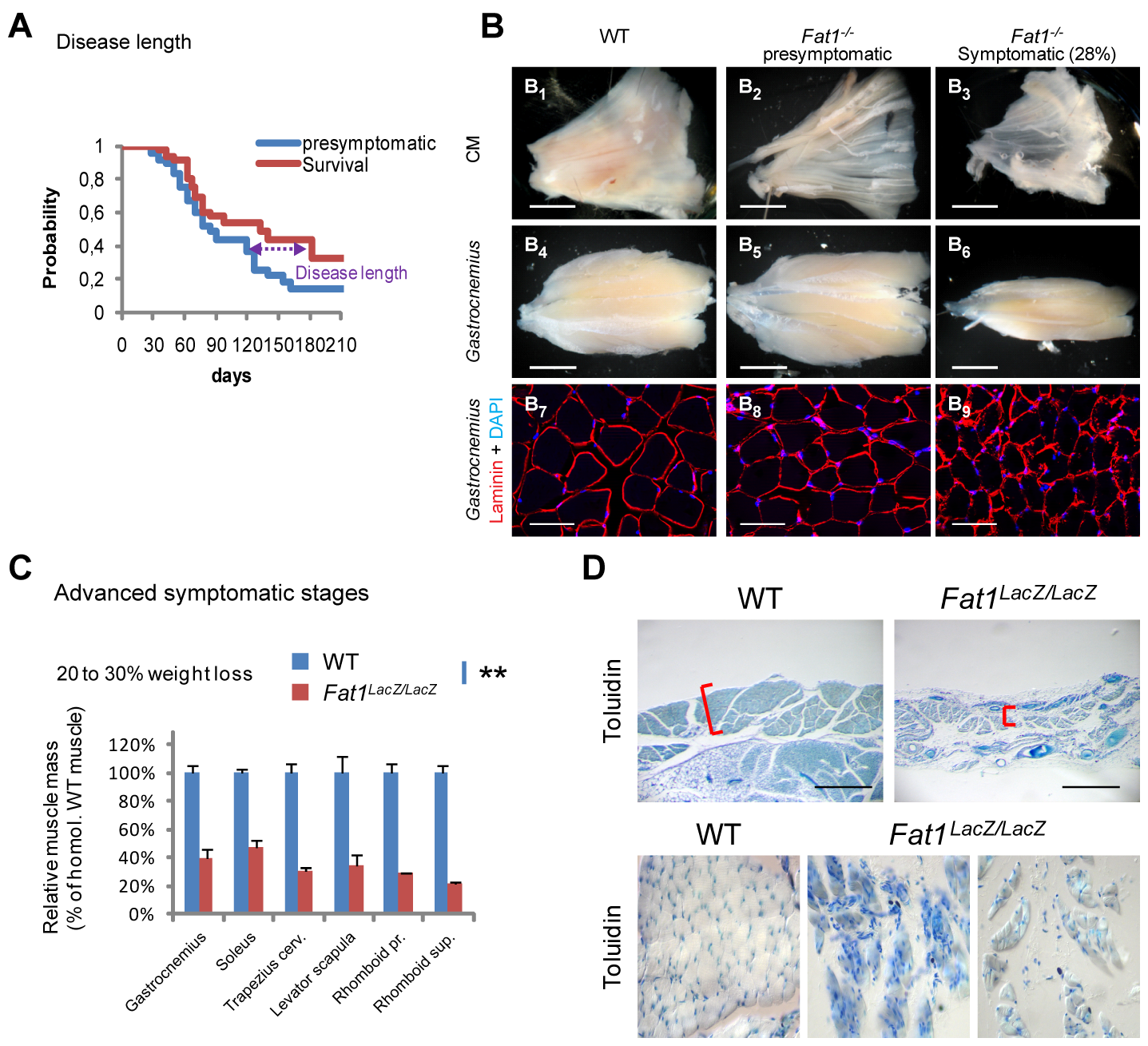

Supplement: Figure S6 — Specific muscle wasting in presymptomatic Fat1LacZ/LacZ mice turns to generalized muscle wasting in symptomatic Fat1LacZ/LacZ mice. (A) Kaplan-Meier plot showing the relationship between disease span and age of onset, as evidenced through the comparison of the probability of onset (blue curve: probability of being presymptomatic) with the survival curve (probability of survival), on the same set of 49 Fat1LacZ/LacZ mice. Fat1LacZ/LacZ mice with early onset show short disease span, while the disease span can be long for mice with late onset. (B) Extent of muscle wasting in adult Fat1LacZ/LacZ mice at presymptomatic and symptomatic stages of phenotype progression. Muscle dissection in adult wild type (B1,4) and Fat1LacZ/LacZ mice at presymptomatic (B2,5) or advanced symptomatic (B3,6; 28% weight loss) stages reveals a pronounced reduction in volume and thickness of the CM muscle at both stages, while Gastrocnemius muscle in the hindlimb only show pronounced wasting at later stages or phenotype progression. Because of the tight association with the skin of its caudal, the CM cannot easily be dissected for weight measurements. In pictures in B1,2,3, this skin-associated caudal part was therefore arbitrarily cut, preventing fair assessment of its true volume. The CM was consequently not included in the relative muscle mass measurements in Figure 3D and Figure S4 (C). (B7–9) Myofibre diameter within the Gastrocnemius muscle was visualized on transverse sections from wild type and Fat1LacZ/LacZ mice at presymptomatic stage (B8) or at advanced symptomatic stage (B9, 20% weight loss), using antibodies against laminin. (C) Masses of dissected muscles of Fat1LacZ/LacZ mice at advanced (20–30% weight loss, n = 3) disease stages relative to age-matched controls (n = 6; average wild type weight defined as 100%). (D) Histological section of Cutaneous maximus muscles from adult wild type and Fat1LacZ/LacZ mice at presymptomatic stage, lightly stained with Toluidin Blue, showing red [file pgen.1003550.s006.tif]

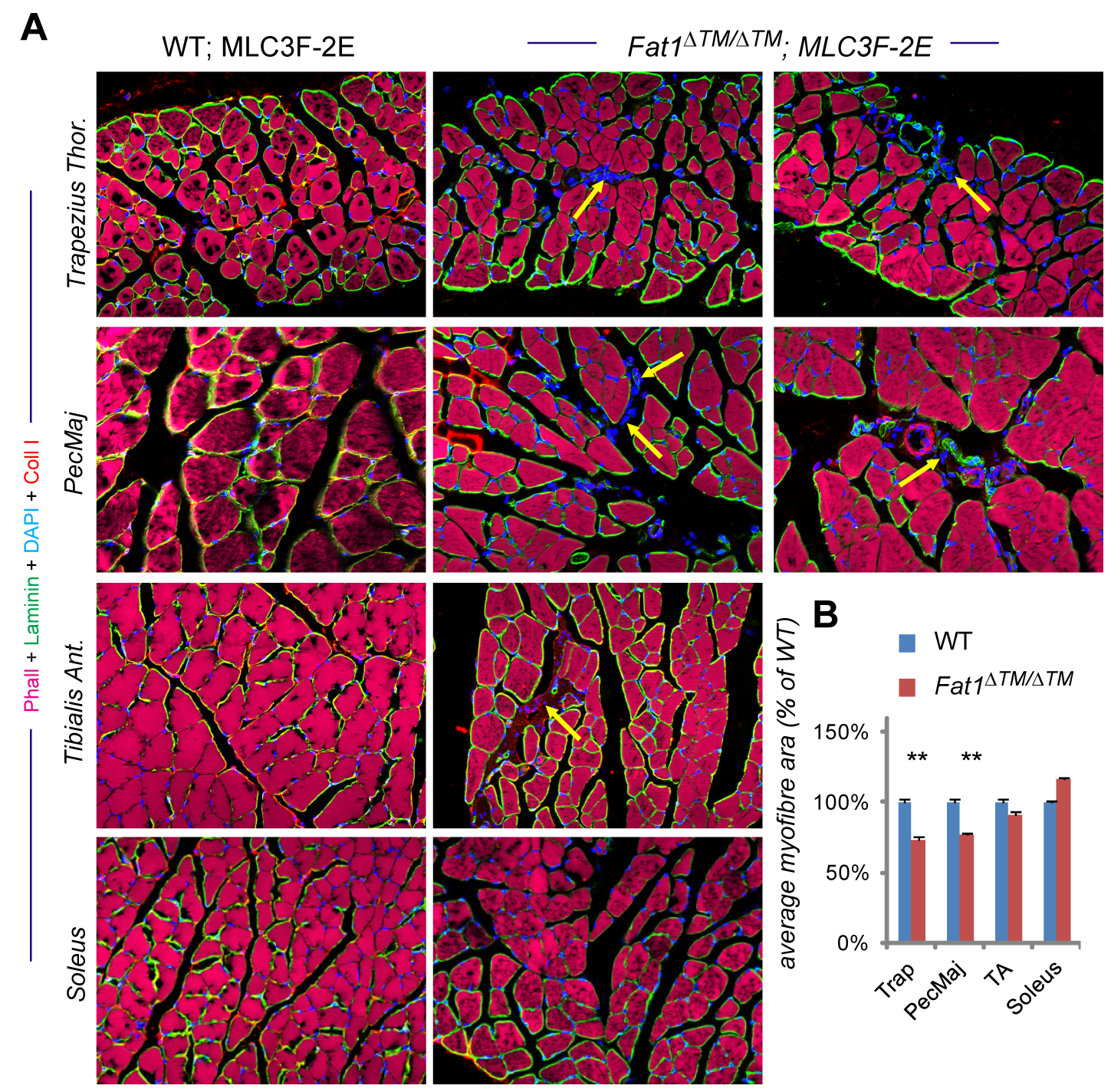

Supplement: Figure S7 — Reduced myofiber diameter and cellular infiltrations in affected muscles from adult Fat1ΔTM/ΔTM mice. (A) Cryosections of the Trapezius Thoracis, Pectoralis Major, Tibialis anterior, and Soleus muscles from adult wild type and presymptomatic Fat1ΔTM/ΔTM mice (4 months old) were stained with phalloidin-Alexa647 (purple), anti-Laminin (green), Collagen I (red), and DAPI (blue). The selected areas illustrate places in the trapezius and Pectoralis Major muscles with cellular infiltrations (yellow arrows) between myofibres, with two examples (right pictures) of perivascular infiltrations. In tibialis Anterior, the selected area contains nuclei of infiltrated cells surrounded with Collagen I-positive deposit (yellow arrow). (B) Average myofiber diameters were quantified in the analysed muscles, and presented as %age of the area of the corresponding Wild type muscle. Myofiber diameter is significantly smaller in Trapezius (** p<0.001), in Pectoralis Major (** p<0.001), and in Tibialis anterior (* p<0.01), but not in soleus. (TIF) [file pgen.1003550.s007.tif]

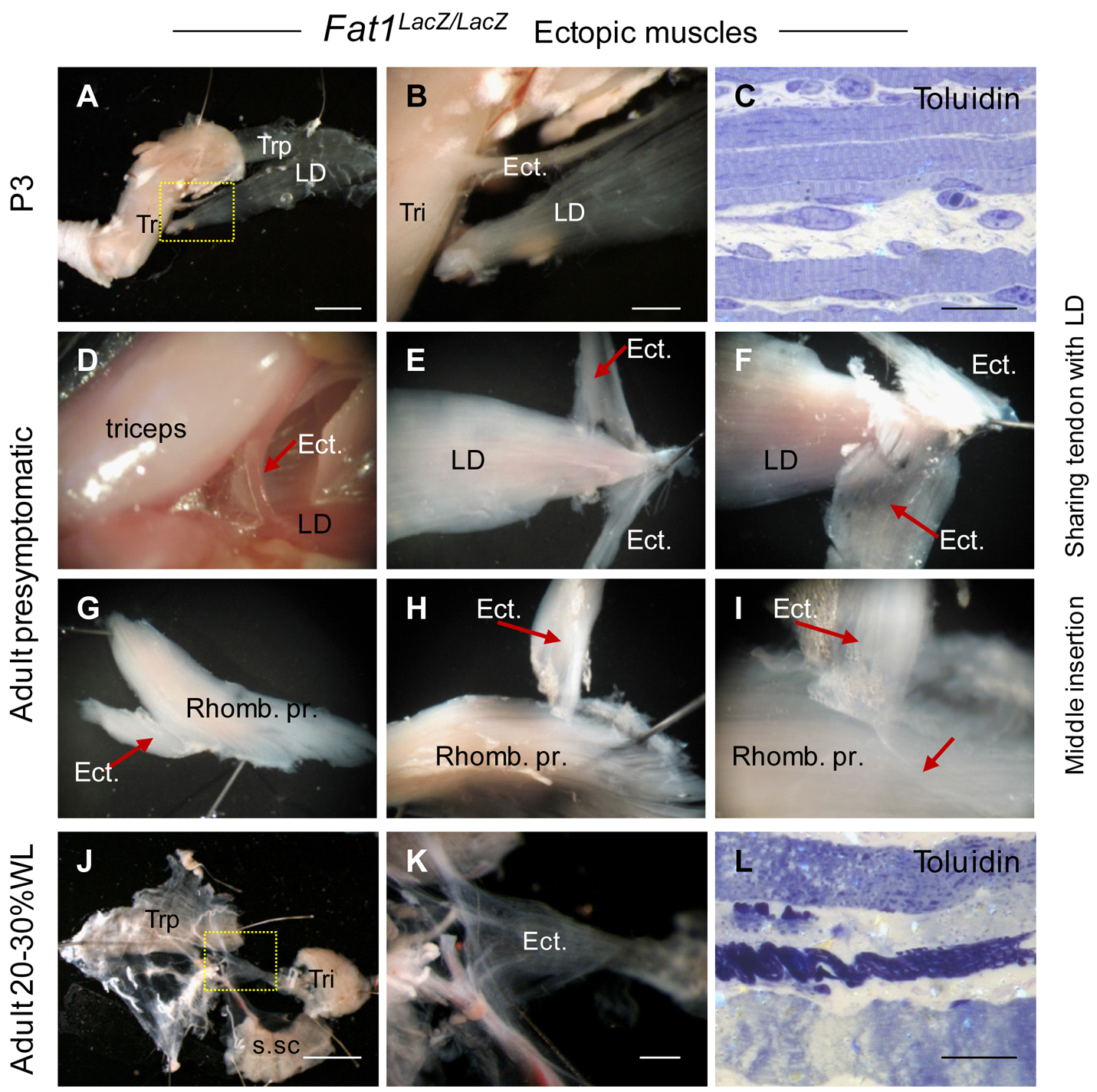

Supplement: Figure S8 — Ectopic muscles variably connecting shoulder or humeral muscles, which are subject to loss of integrity at adult stages. Ectopic muscles (Ect.) were identified during dissection of shoulder or limb musculature from P3 Fat1LacZ/LacZ pups (A–C) and several adult Fat1LacZ/LacZ mice at presymptomatic (D–I) or advanced (J–L) stages of disease progression. Each ectopic muscle was first photographed in its original context (except rhomboids, where the dissection procedure makes it impossible), to visualise original attachment sites with other muscles, and at higher magnification, after dissection pinned on sylgard, without disconnecting the ectopic muscles from the limb or shoulder muscles they were connected to. (C, L) Toluidin counterstained semithin sections of the ectopic muscles seen in (A and B) and (J and K), respectively. Images show that myofibers properly assemble the contractile apparatus (C, P3) but display severe alterations of their histology at adult stages (L). LD: latissimus dorsi; Trp: trapezius; Tri: triceps brachii; s. sc: subscapularis muscle. Scale bars: (A) 2 mm; (B) 0,5 mm; (D) 6 mm; (E) 1 mm; (C, F) 20 µm. (TIF) [file pgen.1003550.s008.tif]

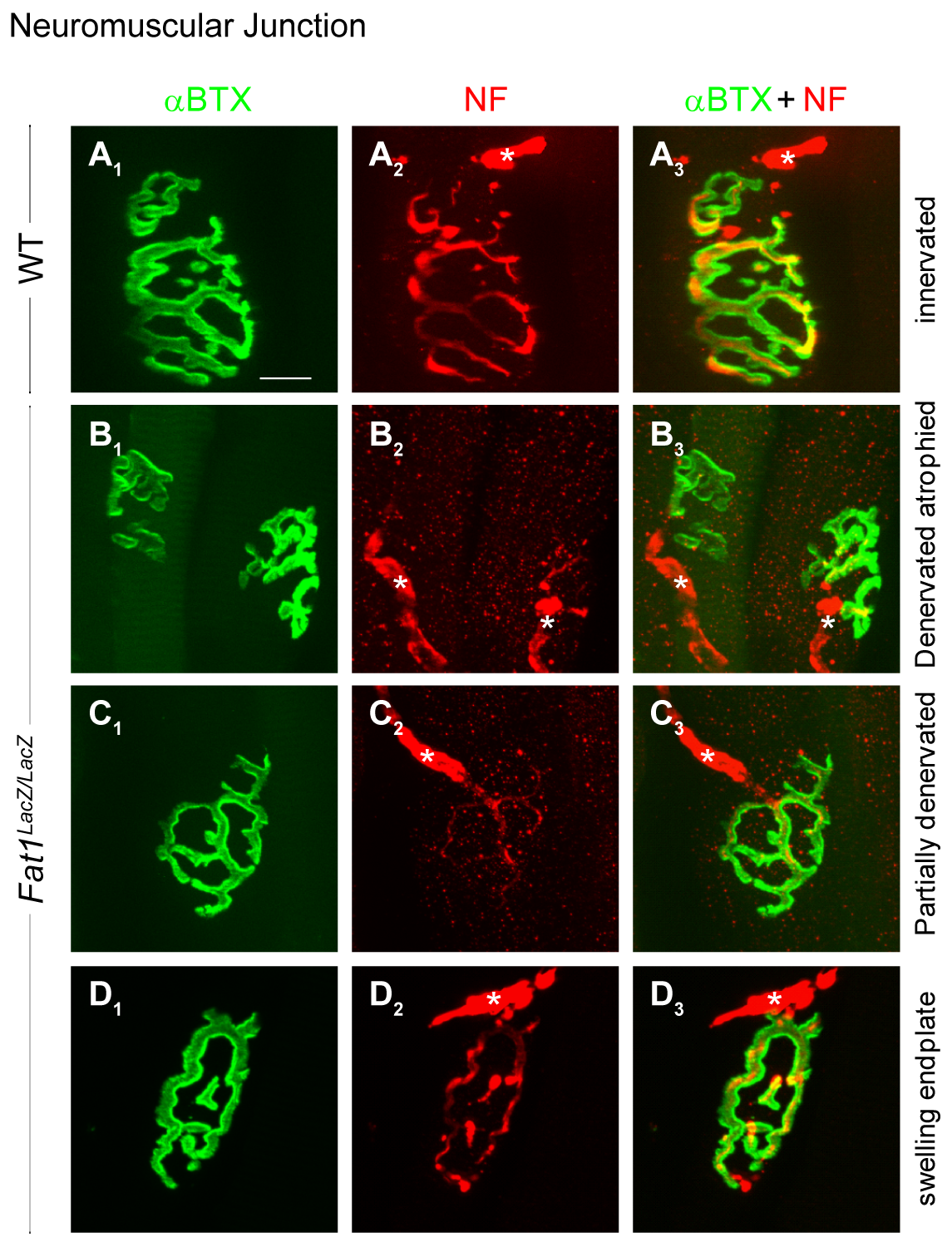

Supplement: Figure S9 — Examples of NMJ denervation and atrophy in Fat1LacZ/LacZ mice. NMJs were visualized in wild type (A1–A3) and Fat1LacZ/LacZ (B1–D3) rhomboid muscles by immunolabelling AChR clusters with α-bungarotoxin (α-BTX, green), and nerve endings with neurofilaments (NF, red). Each synapse is shown with separate α–BTX-green (1) and NF-red channels (2) separately, and with a merge image of both channels (3). In wild type synapses, the NF-positive axon endplate overlaps with all circumvolutions of the bretzel-like shaped α-BTX-positive postsynaptic area. A portion of axon proximal to the NMJ is intensely stained with NF (white stars in all panels). By contrast, Fat1LacZ/LacZ synapses display reduced α-BTX-positive surface, with fragmentation, interruptions, swelling, or absence of the axon endplate, although the NF-positive axon (white starts) is still detectable. (TIF) [file pgen.1003550.s009.tif]

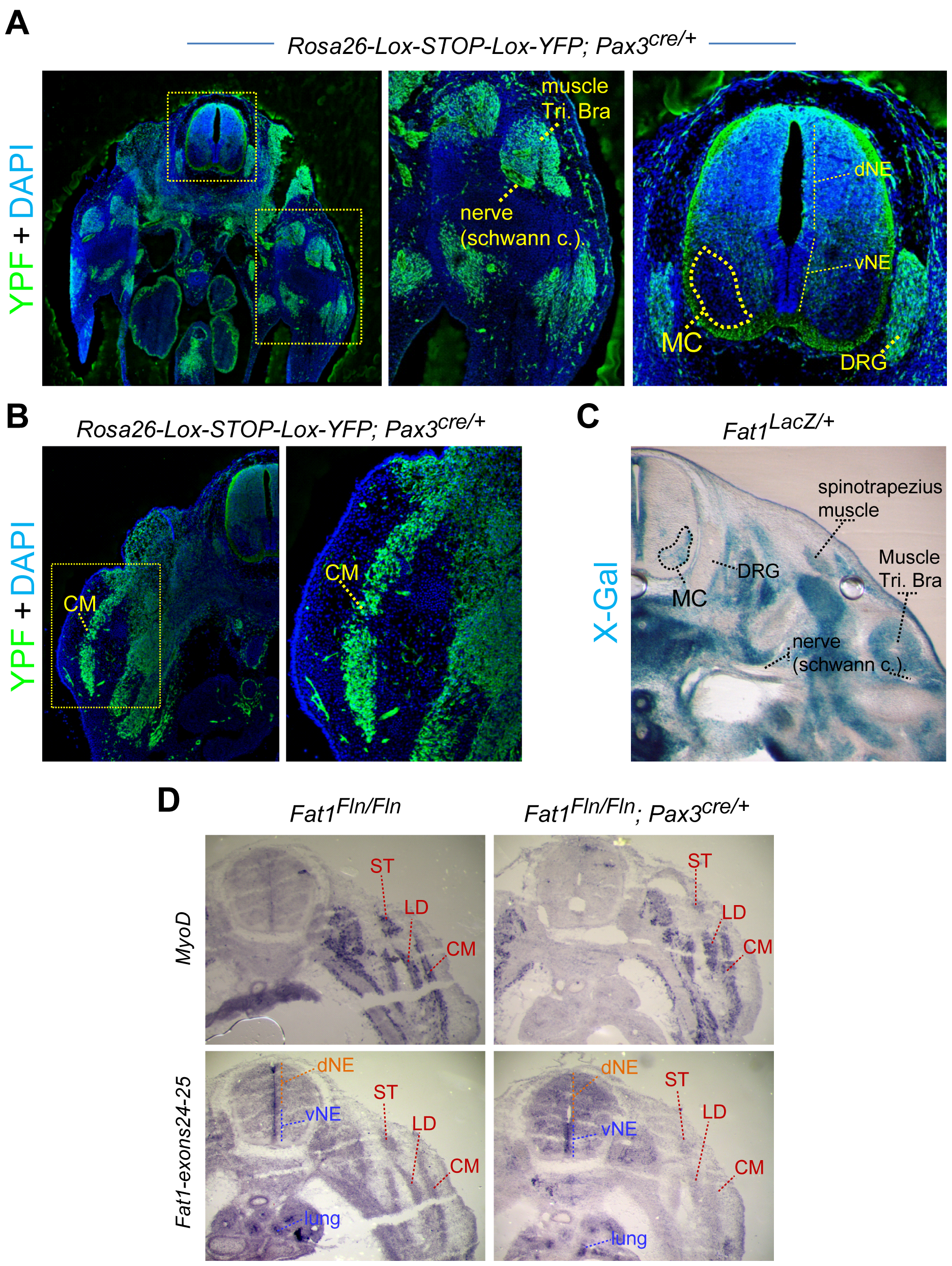

Supplement: Figure S10 — Tissue-Specificity of Pax3-cre recombinase activity. (A, B) Pax3-cre activity was assayed by performing anti-YFP immunohistochemistry (rabbit anti-GFP antibody, Invitrogen) on cryosections of an E12.5 Pax3cre/+; Rosa26Lox-STOP-Lox-YFP/+ embryo. (A) Section at Forelimb level, showing muscle masses in the limbs. The two magnified areas show the limb and spinal cord region. In the limb, besides its activity in muscles, Pax3-cre also leads to excision in neural crest derivatives including Schwann cells along the nerves. In the Spinal cord, Pax3-cre-derived lineage includes dorsal neural precursors and their derivatives mostly confined dorsally. Motor neurons in the motor columns do not express YFP, hence are not part of the Pax3-cre lineage. (B) a slightly posterior section shows efficiency of Pax3-cre activity in the Cutaneous maximus muscle. (C) Section of an E12.5 Fat1LacZ/+ embryo stained with X-gal at a forelimb level comparable to that shown in (A), illustrating Fat1 expression in the same muscle mass as that expressing YFP in the magnified area in (A), as well as in a cervical motor neuron pool, throughout the ventricular zone, and in multiple non muscle sites, including the vertebral bodies. (D) In situ hybridization was performed with antisense probes for myoD (top) and for Fat1-exon24/25 (the floxed exons) on alternate cryostat sections of a Fat1Fln/Fln and a Fat1Fln/Fln; Pax3cre/+ E12.5 embryos, at a level slightly posterior to that shown in (B). In contrast to Fat1 expression in the ventral neuroepithelium (vNE) or in the lung, which is preserved because it does these tissue-types do not derive from Pax3-expressing precursors, Fat1 expression is reduced or abolished in Pax3-derived cell types, such as the dorsal neuroepithelium (dNE) or muscles (CM, LD and spinotrapezius are indicated). (TIF) [file pgen.1003550.s010.tif]

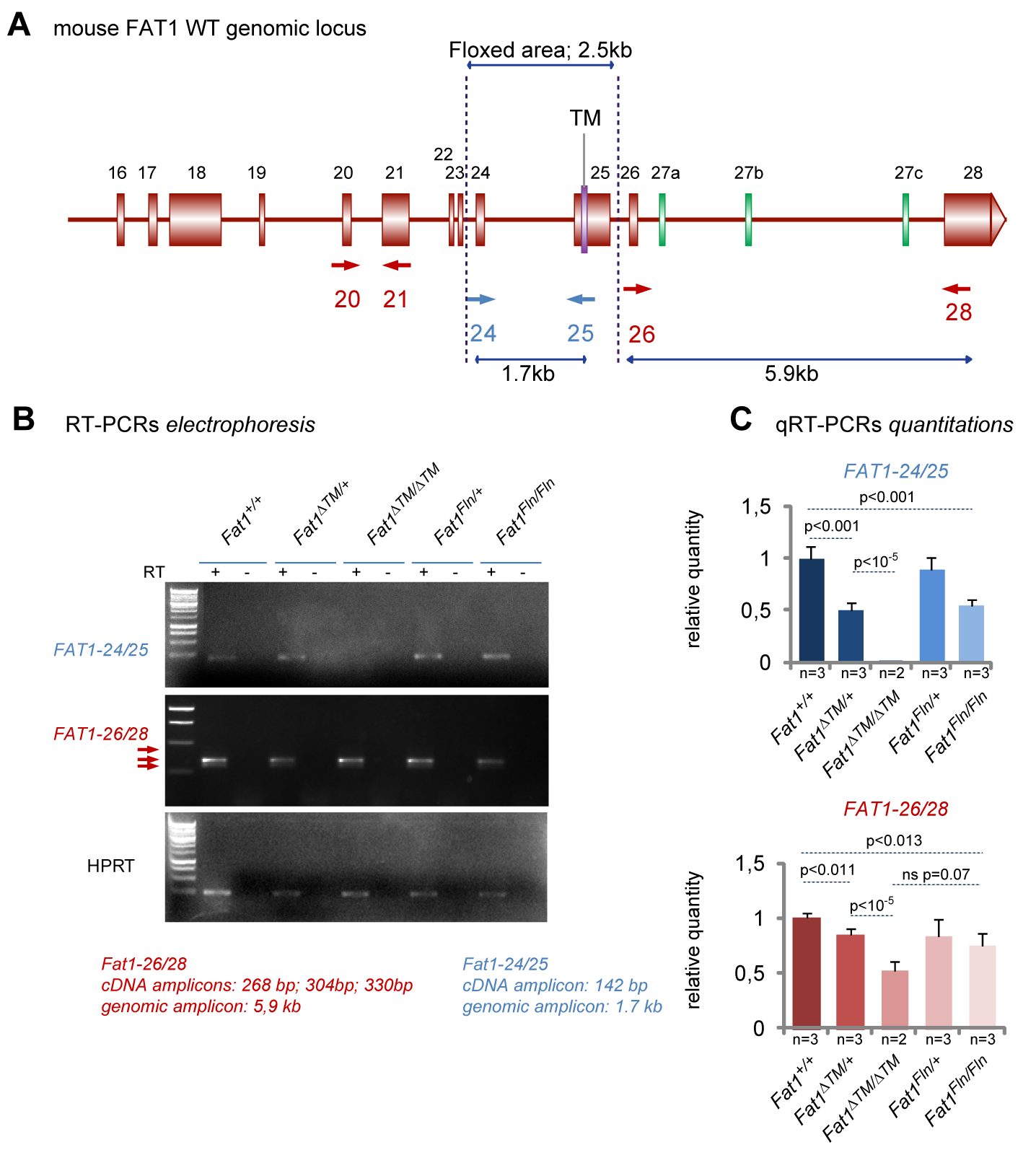

Supplement: Figure S11 — Fat1 RNA levels are mildly affected by the conditional strategy. (A) Scheme of the murine Fat1 genomic locus, showing exon/intron structure, highlighting the area that has been floxed in the conditional allele, as well as the positions of primers that have been used for quantitative RT-PCR studies on mouse tissues. (B,C) RT-PCR studies were performed on RNA preparations from embryos of the indicated genotypes, to evaluate the impact of Fat1 targeting on expression of Fat1 mRNAs containing either the floxed exons (24–25), or the last C-terminal exons (26 to 28). (B) All PCRs were performed on cDNA preparations with or without reverse-transcriptases (+ or − RT), and were loaded on agarose gels to validate that the observed amplicons were specifically obtained from cDNA. In all cases, since primers were chosen in two different exons, and the sizes expected from amplicons from cDNA and genomic DNA are indicated. (C) quantitative PCRs were performed on cDNAs from wild type, Fat1ΔTM/+; Fat1ΔTM/ΔTM; Fat1Fln/+ and Fat1Fln/Fln embryos, to measure the relative amount of RNA containing the floxed exons (24–25, top graph, blue), or the last C-terminal exons (26 to 28, bottom graph, red). Data for each genotype were averaged from 3 embryos, and HPRT was used as normalizing gene. (Top graph): As expected, expression of mRNAs containing exons 24–25 is abrogated in Fat1ΔTM/ΔTM embryos, and reduced by 50% Fat1ΔTM/+ in embryos. The presence of a neo cassette in the conditional Fat1Fln allele exerts a mild effect on Fat1 expression, visible through a 50% reduction of the exon 24–25 signal in Fat1Fln/Fln embryos. (Bottom graph): Expression of mRNAs containing the last exons is not abrogated by the constitutive deletion of exons 24–25, and respresents less than 50% in Fat1ΔTM/ΔTM embryos compared to wild-type. In contrast to upstream exons 24–25, expression of RNAs containing exons 26–28 is only moderately influenced by the neo cassette in Fat1Fln/Fln embryos. This suggests that the low [file pgen.1003550.s011.tif]

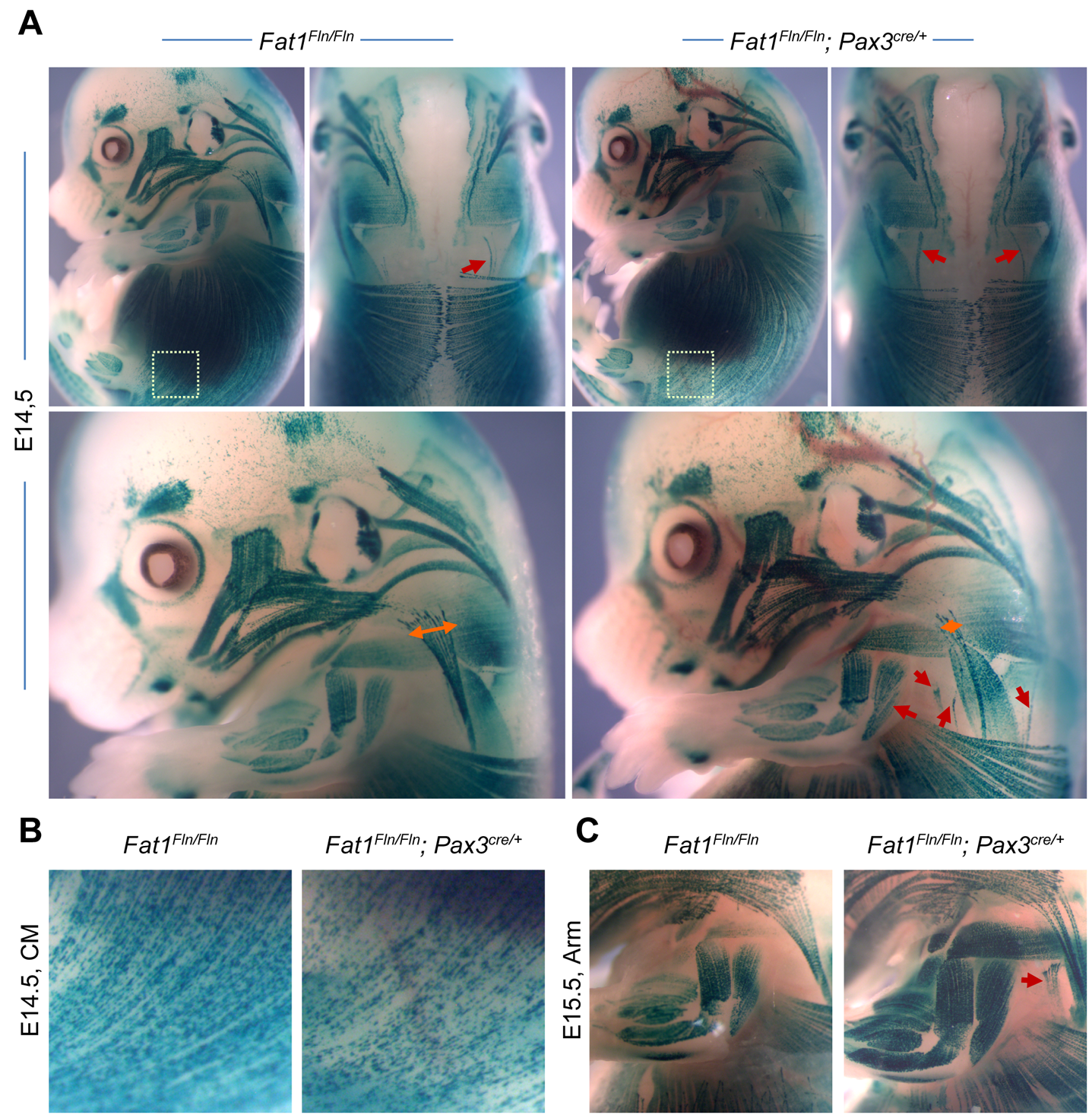

Supplement: Figure S12 — Fat1 ablation in trunk premigratory muscle precursors under Pax3-cre reproduces the scapulohumeral muscle shape phenotypes of the constitutive mutants. Muscle anatomy was visualized at E14.5 (A, B) and E15.5 (C), by X-Gal staining in Fat1Fln/Fln; MLC3F-2E and Fat1Fln/Fln; Pax3cre/+; MLC3F-2E embryos. Mild phenotypes can be detected in Fat1Fln/Fln embryons in the face (reduced occip. Frontalis muscle, and zygomatics), and through appearance of misplaced muscle fibres between Trapezius Cervicalis and Trapezius Thoracis, frequently unilateral or asymmetric (red arrow). While Pax3-cre driven recombination in Fat1Fln/Fln; Pax3cre/+; MLC3F-2E embryos does not cause any worsening in muscle shape and size in the face, abnormalities can be seen in the scapulohumeral region, such as the appearance of an additional muscle, in an ectopic position reminiscent of that seen in Fat1ΔTM/ΔTM embryos, without insertion of its extremity between the spinodeltoid and the Triceps brachii muscles. (TIF) [file pgen.1003550.s012.tif]

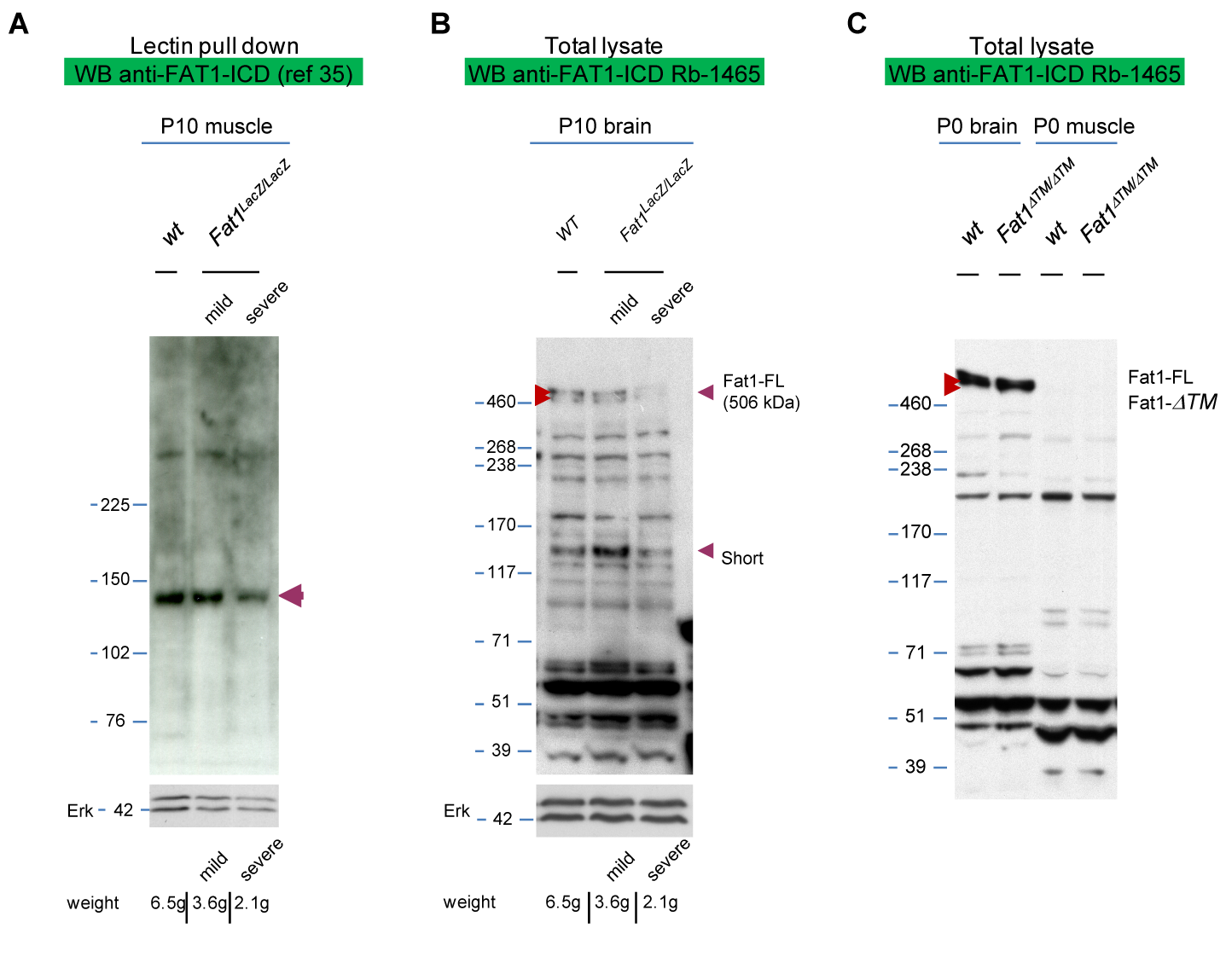

Supplement: Figure S13 — Residual FAT1 protein isoforms are produced in Fat1LacZ/LacZ and Fat1ΔTM/ΔTM mice. Residual FAT1 protein levels in Fat1LacZ/LacZ mice inversely correlate with phenotype severity. (A) Western blot analysis on Lectin-purified muscle protein extracts from 9 days old pups, comparing a wild type and two Fat1LacZ/LacZ cases with different phenotype severity, was performed using a previously characterized anti-FAT1-ICD antibody from ref [35]. (B, C) Western blot analysis was performed with: (B) total brain protein lysates from the same cases shown in (A), or with (C) brain and muscle protein lysates from wild type and Fat1ΔTM/ΔTM P0 pups. Membranes with were blotted with anti-FAT1 antibodies (Rb-1465). ERKs protein levels were used as loading controls (lower panels in A and B). Mutant Fat1LacZ/LacZ mice survive postnatally with variable phenotype severity (see Figure 3C). In the two examples shown here, both Fat1LacZ/LacZ mutants showed strong phenotypes since birth including impaired growth, with a milder case weighing 3.6 g and a severe case weighing 2.1 g (where reduced weight reflects phenotype) compared to their wild type littermates weighing 6.5 g in average. (TIF) [file pgen.1003550.s013.tif]

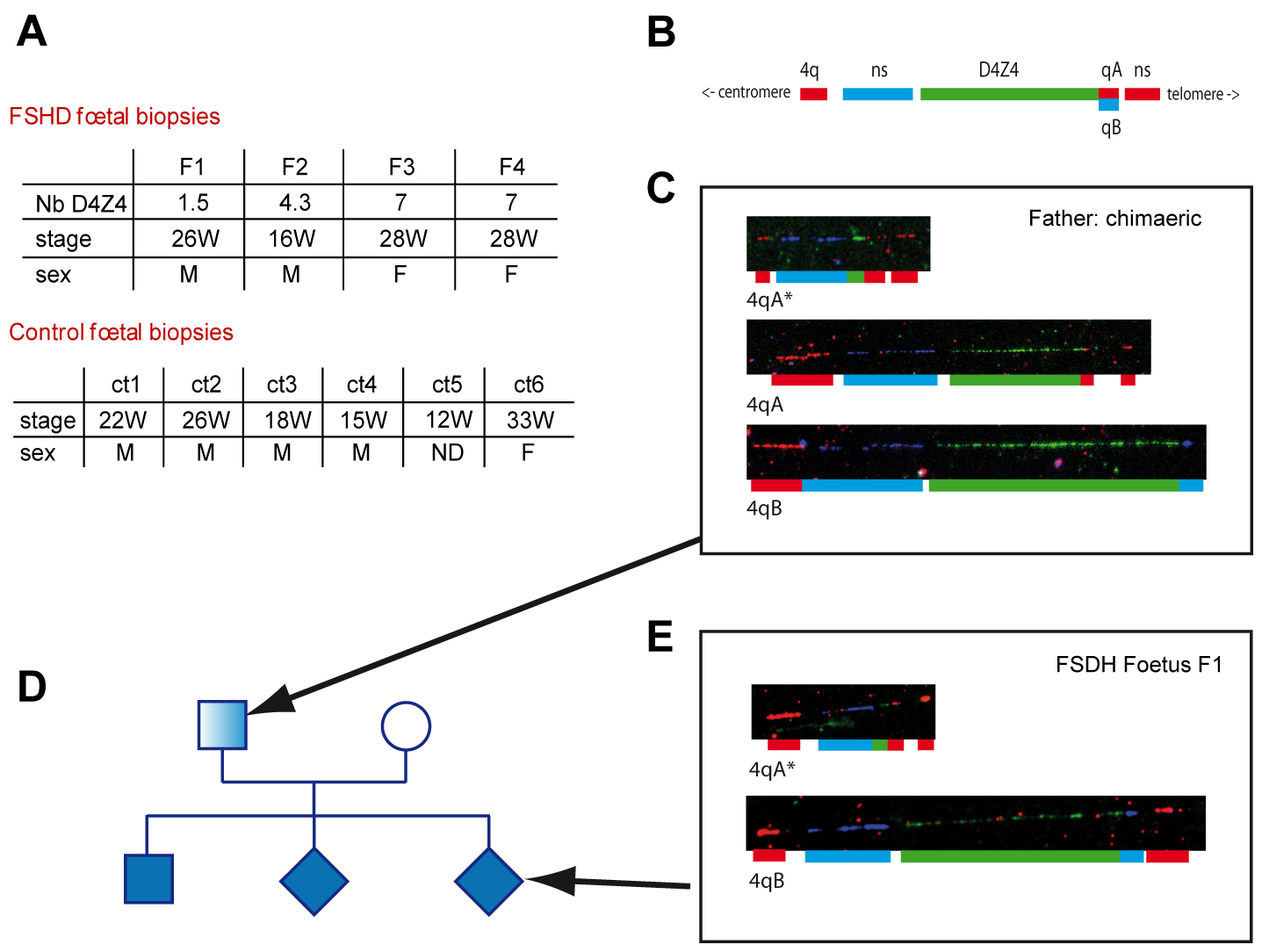

Supplement: Figure S14 — Characteristics of human foetal FSHD1 and control cases. (A) Tables listing the FSHD1 foetuses (top) and control fetuses (bottom) from which biopsies were used for the present study. These tables indicate for each case, the ID symbol, the stage at which termination of pregnancy was performed (in weeks of amenorrhea), their sex, and for FSHD1 cases, the number of D4Z4 microsatellite repeats. (B) Scheme representing the design of DNA probes and fluorophores used for genotyping FSHD patients by molecular combing, in combed genomic DNA as described in ref [94]. (C) Molecular combing genotyping results showing 3 different alleles of 4q35 were detected in the genome of one male individual, chimaeric carrier of an FSHD1 allele: One 4qB allele (blue, right side), one “normal” 4qA allele with a long D4Z4 strech (green), and one contracted 4qA allele (4qA*), qualifying as FSHD1. This individual was not diagnosed with FSHD, most likely owing to his degree of chimaerism, but was the father (family tree shown in (D) of one first child with severe, early onset FSHD, and one foetus diagnosed with FSHD as well through prenatal diagnosis (one of which was used in this study as F1;genotype shown in E). (D) Family tree showing the distribution of FSHD symptoms in this patient family. (E) Molecular combing genotyping of the F1 FSHD foetus, showing one 4qA* contracted FSHD1 allele inherited from the father, and one 4qB allele inherited from the mother (distinct from the father's 4qB). (TIF) [file pgen.1003550.s014.tif]

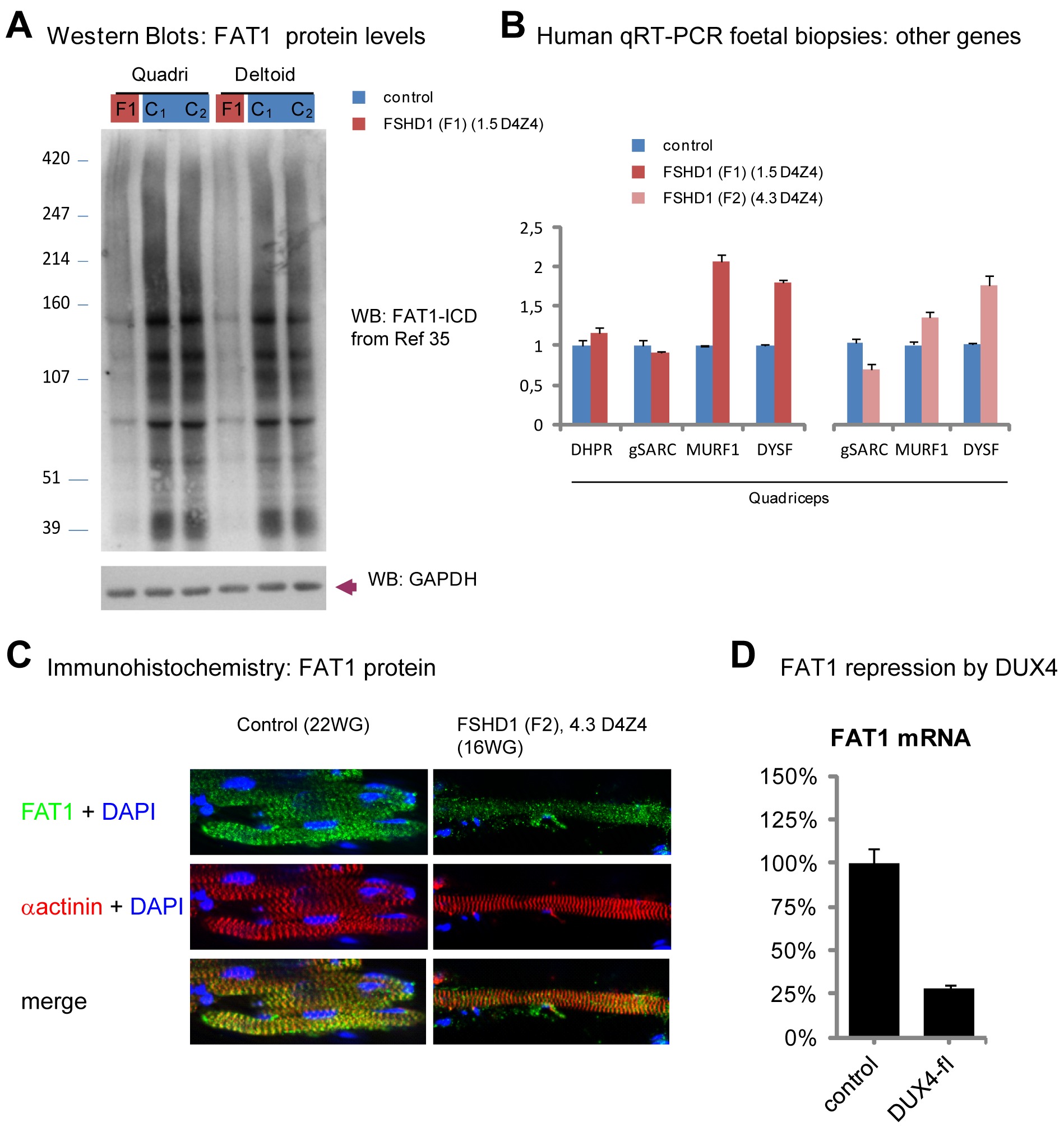

Supplement: Figure S15 — qRT-PCR and immunohistochemistry data from foetal FSHD muscle. (A) Western blot analysis of Fat1 levels in muscle protein extracts from the 26 weeks old FSHD Foetus with 1.5 D4Z4 repeats (F1) and 2 age-matched control foetuses (C1 and C2) with the anti-FAT1-ICD antibody from ref [35]. (B) qPCR analysis of mRNA levels of several genes other than FAT1 (shown in Figure 7) involved in muscle biology (DHPR, γ-sarcoglycan (γSARC), MURF1, DYSF) in quadriceps muscles of a 26 weeks old FSHD1 foetus (F1) harbouring 1.5 D4Z4 repeats in the 4q35 region (dark red bars), and a 16 weeks old FSHD1 foetus harbouring 4.3 D4Z4 repeats at 4q35 region (F2), respectively compared with age-matched control foetuses (blue bars). (C) Immunolocalization of FAT1 (Rb-1465 anti FAT1-ICD, green) and α-actinin (αact, red) in longitudinal sections from human quadriceps biopsies from a control (top) or and the FSHD1 (F2, bottom) foetus with 4.3 D4Z4 repeats. (D) Quantitative PCR shows that DUX4-fl downregulates expression of FAT1 in human primary myoblasts. DUX4-fl or GFP (control) were expressed in unaffected muscle cells by lentiviral delivery. Data were normalized to internal standard RPL13a and represented as mean +/−SD of triplicates with control set at 100%. (TIF) [file pgen.1003550.s015.tif]

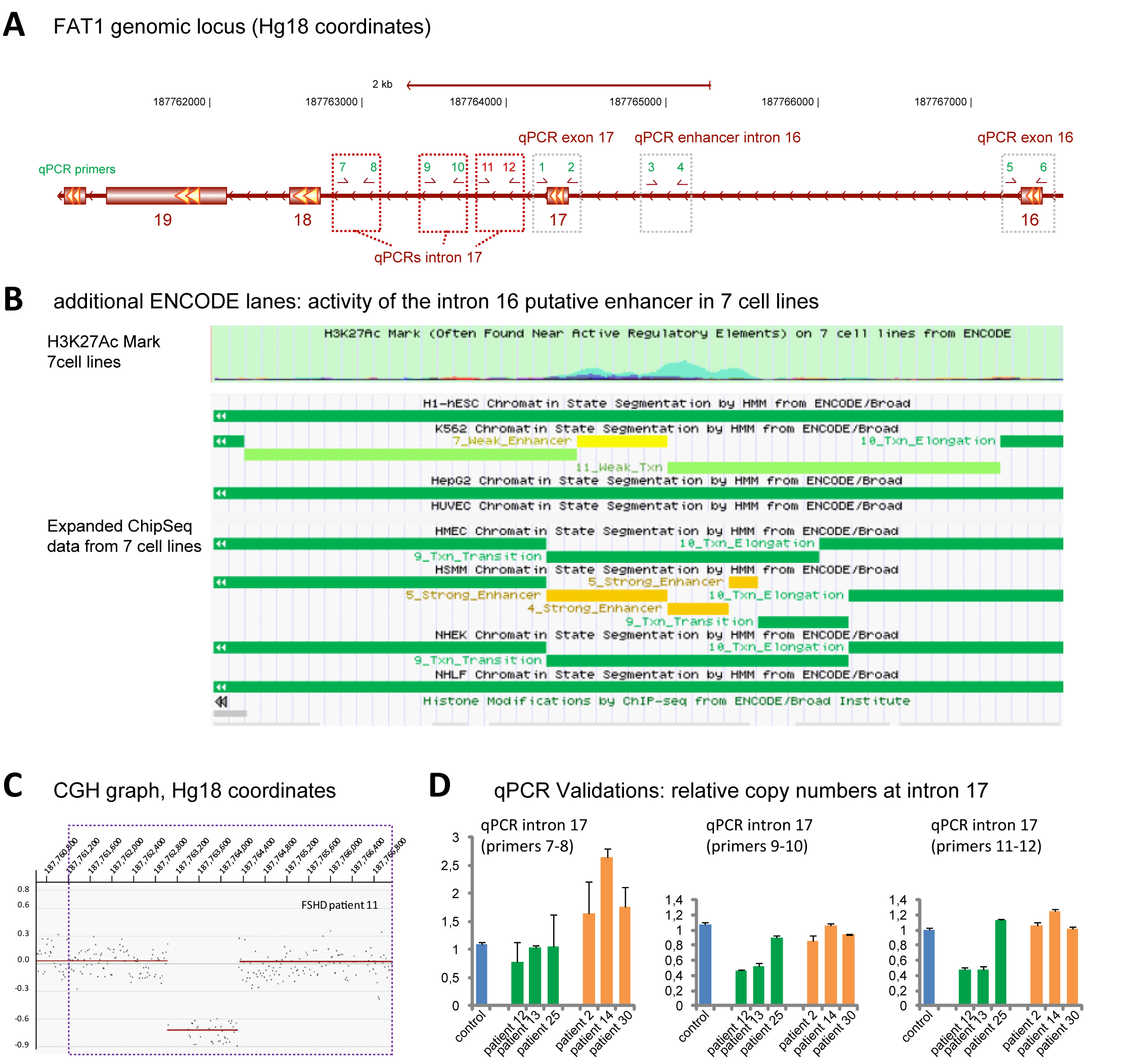

Supplement: Figure S16 — Custom array CGH analyses of genome copy number changes in the 4q35.2 region. (A) Scheme of the genomic region in which CNVs were identified. The region represented is identical to that shown in Figure 10. The dotted line boxes represent positions of the PCR primers used for qPCR validation of copy number variants. The boxes in grey are those shown in Figure 10, the brown ones represent position of the primers for qPCRs shown in (D). (B) Screen copies of USCC browser lanes matching the position shown in (A), representing ENCODE-derived data (available on http://genome.ucsc.edu). The “layered H3K27Ac track”, shows enrichment of the H3K27Ac histone mark across the genome as determined in 7 cell lines by a ChIP-seq assay (the H3K27Ac histone mark is the acetylation of lysine 27 of the H3 histone protein). The lower tracks represent expanded image showing a chromatin state segmentation [67] for each of 7 of the nine human cell types, computationally integrating ChIP-seq data for nine factors plus input. The intron 16 ehancer appears labelled as exhibiting strong enhancer activity in HSMM cells (human skeletal myoblast muscle cells). (C) Genome copy number variation frequencies are plotted as a function of position in the same region as that shown in (A). Chromosome locations (NCBI36hg18 build) are indicated by numbers above graph. Negative values (log2ratio<−0.3) indicate frequencies of probes showing copy number decreases between DNA of patient 11 respect to a control DNA. The extent of deletion is of around 1 kilobase. (D) Copy number validation of the deletion by qPCR. The relative amounts of a PCR fragments obtained using primers couples indicated in (A), which amplification corresponds to the control allele (blue bars), were compared between a control patient DNA (with 2 copies of the normal allele), and three C.I.FSHD patients carrying one copy of a deleted allele (green bars); but also three additional c.i.FSHD patients (orange bars) that did not show copy number [file pgen.1003550.s016.tif]
